# Supplementary material for: Assessing and predicting adolescent and early adulthood common mental disorders using electronic primary care data: analysis of a prospective cohort study (ALSPAC) in Southwest England
Source: BMJ Open. 2021 Oct 18;11(10):e053624. doi: 10.1136/bmjopen-2021-053624 (PMC8524296; doi:10.1136/bmjopen-2021-053624)
Supplement: Author's manuscript [file bmjopen-2021-053624.draft_revisions.pdf]

BMJ Open is committed to open peer review. As part of this commitment we make the peer review history of every article we publish publicly available.

When an article is published we post the peer reviewers' comments and the authors' responses online. We also post the versions of the paper that were used during peer review. These are the versions that the peer review comments apply to.

The versions of the paper that follow are the versions that were submitted during the peer review process. They are not the versions of record or the final published versions. They should not be cited or distributed as the published version of this manuscript.

BMJ Open is an open access journal and the full, final, typeset and author-corrected version of record of the manuscript is available on our site with no access controls, subscription charges or pay-per-view fees (http://bmjopen.bmj.com).

If you have any questions on BMJ Open's open peer review process please email info.bmjopen@bmj.com

BMJ Open

Assessing and predicting adolescent and early adulthood common mental disorders in the ALSPAC cohort using electronic primary care data

Journal:	*BMJ Open*
Manuscript ID	bmjopen-2021-053624
Article Type:	Original research
Date Submitted by the Author:	18-May-2021
Complete List of Authors:	Smith, Daniel; University of Bristol, MRC Integrative Epidemiology Unit; University of Bristol, Population Health Sciences, Bristol Medical School<br>Willan, Kathryn ; Bradford Teaching Hospitals NHS Foundation Trust, Born in Bradford<br>Prady, Stephanie; University of York, Department of Health Sciences<br>Dickerson, Josie; Bradford Teaching Hospitals NHS Foundation Trust, Born in Bradford<br>Santorelli, Gillian; Bradford Teaching Hospitals NHS Foundation Trust, Born in Bradford<br>Tilling, Kate; University of Bristol, MRC Integrative Epidemiology Unit; University of Bristol, Population Health Sciences, Bristol Medical School<br>Cornish, Rosie; University of Bristol, MRC Integrative Epidemiology Unit; University of Bristol, Population Health Sciences, Bristol Medical School
Keywords:	Depression & mood disorders < PSYCHIATRY, PRIMARY CARE, Anxiety disorders < PSYCHIATRY, EPIDEMIOLOGY, Child & adolescent psychiatry < PSYCHIATRY

SCHOLARONE™
Manuscripts

I, the Submitting Author has the right to grant and does grant on behalf of all authors of the Work (as defined in the below author licence), an exclusive licence and/or a non-exclusive licence for contributions from authors who are: i) UK Crown employees; ii) where BMJ has agreed a CC-BY licence shall apply, and/or iii) in accordance with the terms applicable for US Federal Government officers or employees acting as part of their official duties; on a worldwide, perpetual, irrevocable, royalty-free basis to BMJ Publishing Group Ltd ("BMJ") its licensees and where the relevant Journal is co-owned by BMJ to the co-owners of the Journal, to publish the Work in this journal and any other BMJ products and to exploit all rights, as set out in our licence.

The Submitting Author accepts and understands that any supply made under these terms is made by BMJ to the Submitting Author unless you are acting as an employee on behalf of your employer or a postgraduate student of an affiliated institution which is paying any applicable article publishing charge ("APC") for Open Access articles. Where the Submitting Author wishes to make the Work available on an Open Access basis (and intends to pay the relevant APC), the terms of reuse of such Open Access shall be governed by a Creative Commons licence – details of these licences and which Creative Commons licence will apply to this Work are set out in our licence referred to above.

Other than as permitted in any relevant BMJ Author's Self Archiving Policies, I confirm this Work has not been accepted for publication elsewhere, is not being considered for publication elsewhere and does not duplicate material already published. I confirm all authors consent to publication of this Work and authorise the granting of this licence.

For peer review only

Assessing and predicting adolescent and early adulthood common mental disorders in the ALSPAC cohort using electronic primary care data

Daniel Smith[1,2], Kathryn Willan[3], Stephanie L Prady[4], Josie Dickerson[3], Gillian Santorelli[3], Kate Tilling[1,2], Rosie P Cornish[1,2]

[1] MRC Integrative Epidemiology Unit at the University of Bristol, UK
[2] Population Health Sciences, Bristol Medical School, University of Bristol, UK
[3] Born in Bradford, Bradford Teaching Hospitals NHS Foundation Trust, Bradford, UK
[4] Department of Health Sciences, University of York, UK

Corresponding authors: Daniel Smith (dan.smith@bristol.ac.uk) and Rosie P Cornish (Rosie.Cornish@bristol.ac.uk)

ORCIDs:
DS: 0000-0001-6467-2023
GS: 0000-0003-0427-1783
SLP: 0000-0002-8933-8045
RC: 0000-0002-2874-7646

Word count: 5,395
Tables: 5
Figures: 2

For peer review only

Abstract

Objectives: This paper has three objectives: 1) examine agreement between common mental disorders (CMDs) derived from primary health care records and repeated CMD questionnaire data from ALSPAC (the Avon Longitudinal Study of Parents and Children); 2) explore the factors affecting CMD identification in primary care records; and 3) taking ALSPAC as the reference standard, to construct models predicting ALSPAC-derived CMDs using primary care data.

Design and Setting: Prospective cohort study (ALSPAC) with linkage to electronic primary care data.

Participants: Primary care records were extracted for 11,807 ALSPAC participants (80% of the 14,731 eligible participants). The number of participants with both linked primary care and ALSPAC CMD data varied between 3,633 (age 15/16) to 1,298 (age 21/22).

Outcome measures: Outcome measures from ALSPAC data were diagnoses of suspected depression and/or CMDs. For the primary care data, Read codes for diagnosis, symptoms and treatment were used to indicate the presence of depression and CMDs.

For each time point, sensitivities and specificities (using ALSPAC-derived CMDs as the reference standard) were calculated and the factors associated with identification of primary care-based CMDs in those with suspected ALSPAC-derived CMDs explored. Lasso models were then performed to predict ALSPAC CMDs from primary care data.

Results: Sensitivities were low for CMDs (range: 3.5 to 19.1%) and depression (range: 1.6 to 34.0%), while specificities were high (nearly all >95%). The strongest predictor of identification in the primary care data was symptom severity. The lasso models had relatively low prediction rates, especially for out-of-sample prediction (deviance ratio range: -1.3 to 12.6%), but improved with age.

Conclusions: Even with predictive modelling using all available information, primary care data underestimate CMD rates compared to estimates from population-based studies. Research into the use of free-text data or secondary care information is needed to improve the predictive accuracy of models using clinical data.

Keywords: ALSPAC, Common Mental Disorders, Depression, Primary Care Data, Data Linkage, Predictive Modelling

For peer review only

Strengths and limitations of this study

- We used a large prospective cohort (ALSPAC) and were able to link these data to individuals' electronic primary care records, with this linkage data covering ~80% of the cohort.
- We used validated mental health questionnaires to assess depression and common mental disorders among the ALSPAC cohort, which we treat as our 'reference standard'.
- We were able to assess agreement between ALSPAC data and electronic primary care data for common mental disorders across adolescence and into adulthood, a key life transition and period where mental health problems often emerge.
- There is a risk of selection bias, as many participants with primary care data did not have ALSPAC mental health measures, while primary care data coverage also decreased with age; continued participation in both cases is likely to be non-random.
- For this study we assumed that the common mental disorder data from ALSPAC are the 'reference standard' against which the primary care data should be compared; however, this data may also be subject to misclassification.
- The available linkage data consisted of primary care Read codes, which misses data from other clinical sources, such as secondary care or from primary care free-text data.

For peer review only

INTRODUCTION

Common mental disorders (CMDs; depression and anxiety) are a leading cause of morbidity, disability and premature death worldwide [1]. Rates of CMDs have increased over the past few decades [2], including in adolescence and early adulthood [3], where these conditions frequently first appear [4,5]. The prevalence of CMDs in childhood and adolescence (age 5-16) in the UK is estimated to be 4% [6], rising to 16% among 16-24 year-olds [7]; these can have significant long-term consequences, including on education, quality-of-life, employment and physical and mental health [5,8,9].

Assessing the prevalence of CMDs in the population, especially in adolescence, is essential for monitoring, research and planning of appropriate public health services. Estimates of prevalence could be from population studies (which are expensive and time-consuming to conduct), or using primary (General Practitioner; GP) and secondary (hospital and specialised healthcare services) care records [10–13]. However, CMDs are often under-diagnosed in routine primary care data (the so-called 'clinical iceberg' phenomenon), with over half of all depressed patients with clinical symptoms of depression not recognised as such [14,15]. Reasons for this include: individuals with CMDs not visiting their GP [16]; GPs misdiagnosing, or being reticent in diagnosing, CMDs [15]; and GPs increasingly recording symptoms, rather than specific diagnoses [17]. This 'clinical iceberg' may be particularly prevalent among children and adolescents, who may be less likely to visit their GP. Additionally, GPs may fail to identify, or be less willing to diagnose CMDs or prescribe anti-depressants to these groups [18–20]. Primary care physicians frequently refer to secondary care services, such as Child and Adolescent Mental Health Services (CAMHS; [21]), again contributing to the under-reporting of adolescent CMDs in primary care records.

To assess the accuracy of primary care-derived CMD rates, these must be compared against a reference standard [16]. A systematic review in adults found that, relative to a reference standard, specificity is generally high (few false positives) but sensitivity is rather low (many false negatives; [12]). Previous research from the Avon Longitudinal Study of Parents and Children (ALSPAC) compared linked primary care records at age 17/18 against CMDs measured on 1,562 participants via the revised Clinical Interview Schedule (CIS-R) [10]. Using CIS-R as the reference standard, this study found that – similar to findings in adults - sensitivities were low while specificities were high. Together, these findings suggest that primary care data may significantly underestimate the prevalence of CMDs in the population.

Previous UK research has shown that greater symptom severity is the strongest predictor of attending primary care regarding mental health [16]. Other factors, such as age, sex and employment status, also predicted accessing primary care, but their contributions were weaker [16]. In contrast, a smaller US study of individuals with depressive symptoms found no demographic differences between those who sought help and those who did not, although symptom severity again predicted help-seeking behaviour [22]. Sociodemographic factors may play a role in access to primary care, recognition of symptoms, and access to treatment, which contribute to continuing health inequalities [23,24]. For instance, a UK study found that both non-British ethnicity and low socioeconomic position predicted lower rates of CMD detection in primary care records during the maternal period [25]. Even if individuals with a CMD do contact a physician, the likelihood of receiving treatment is also dependent on symptom severity, as well as socio-demographic factors [26,27].

Models predicting 'true' CMD status from variables available in primary care records could help to identify the prevalence of individuals with 'missing' CMDs as well as the factors predicting these

For peer review only - http://bmjopen.bmj.com/site/about/guidelines.xhtml

cases. Previous work has predicted CMDs based on an Australian dataset [28], but did not use primary care records, so its utility may be limited as some relevant factors are unlikely to be present in routine health records (e.g., job satisfaction, social isolation, being a carer, having a partner, etc.). Research using only primary care record data to predict validated measures of CMDs from population-based studies are therefore required.

This study has three aims:

1) Replicate and expand the results of a previous ALSPAC study at age 17/18 (~2,800 participants [10]) by including additional participants with linkage data (~12,000 participants [29,30]), and explore agreement between primary care records and cohort data across multiple time points over adolescence and young adulthood (ages 15-23).
2) Assess the factors impacting rates of identification in primary care records.
3) Construct a prediction model, with ALSPAC-measured CMDs as the outcome, to predict CMD status using only primary care data.

METHODS

Study Design and Participants

ALSPAC is a pregnancy-based longitudinal birth cohort which recruited pregnant women in the Bristol area of southwest England with an expected delivery date between 1st April 1991 and 31st December 1992 [31,32]. AS total of 14,541 eligible pregnancies were initially recruited into the study, with a total of 14,676 foetuses, resulting in 14,062 live births, of which 13,988 were alive at one year of age. After further waves of post-natal recruitment , as of February 2019 there are a total of 14,901 study child participants enrolled in ALSPAC who were alive at one year [30]. These children and their parents have been followed since birth, with detailed data collected via questionnaires, in-person clinic assessments, and linkage to routine data sets. The study website contains details of all available data through a fully searchable data dictionary and variable search tool: http://www.bristol.ac.uk/alspac/researchers/our-data/. From 22 years onwards data were collected and managed using REDCap electronic data capture tools hosted at the University of Bristol [33]. We used the STROBE cohort reporting guidelines [34].

When the study children reached legal adulthood (age 18), ALSPAC initiated a postal fair processing campaign to formally re-enrol the children into the study (prior to this parent-based consent was mandatory, although from age 9 children assented to data collection as well) and to simultaneously seek opt-out permission for ALSPAC to link to their health and administrative records [35]. Linkage to primary care records was carried out following this campaign and electronic primary care records have been extracted for nearly 12,000 study children [30]. This linkage is described in more detail in the supplementary material (see also [29]).

In total, 14,731 ALSPAC participants were eligible for our study, comprising all enrolled singletons and twins who were alive at 1 year of age and had not withdrawn consent from the study. Of this total sample, 13,113 participants were sent fair processing materials, of which 368 (2.8%) dissented to linkage. Primary care records (although not necessarily for the entire time period) were extracted for 11,807 of these individuals (80% of the original 14,731 eligible participants; 90% of the 13,113 sent fair processing materials). Note that there are several dynamic factors that affect inclusion eligibility in these analyses (e.g., study enrolment status and linkage quality to the NHS Person

For peer review only - http://bmjopen.bmj.com/site/about/guidelines.xhtml

Demographics Service, PDS). Therefore, the numbers reported here may differ from the numbers reported in the ALSPAC primary care linkage data note (currently in preparation).

The current study includes ALSPAC data from multiple time points between the ages of 15 and 23 (table 1), from either clinic or questionnaire data collections. The age 15/16 and 17/18 clinics collected data on both depression and anxiety; at the other time points only depression was assessed. Linked primary care record data coverage decreases with age because the linkage data primarily covers the Bristol area; as many participants moved away as they reached adulthood (e.g. for university or work) they are lost from the linked dataset.

Table 1: Details of ALSPAC data used and coverage with primary care linkage data.

Age (time point)	Measure	# with ALSPAC CMD data	# of these with primary care data (%)
**Age 15/16 (TF3 clinic)**	DAWBA (Depression & anxiety)	5,332	3,663 (68.7%)
**Age 16/17 (CCS questionnaire)**	SMFQ (Depression only)	4,950	3,213 (64.9%)
**Age 17/18 (TF4 clinic)**	CIS-R (Depression & anxiety)	4,534	3,084 (68%)
**Age 18/19 (CCT questionnaire)**	SMFQ (Depression only)	3,302	1,982 (60%)
**Age 21/22 (YPA questionnaire)**	SMFQ (Depression only)	3,283	1,298 (39.5%)
**Age 22/23 (YPB questionnaire)**	SMFQ (Depression only)	3,896	1,325 (34%)

DAWBA: Development and Well-Being Assessment; *SMFQ*: Short Mood and Feelings Questionnaire; *CIS-R:* Clinical Interview Schedule – Revised.

Patient and Public Involvement Statement

ALSPAC has an advisory panel of >30 participants who meet bimonthly to advise on study design, methodology and acceptability. ALSPAC communicates with participants via regular newsletters and has an active website and social media presence.

ALSPAC data

At the age 15/16 clinic, depression and anxiety were assessed using the Development and Well-Being Assessment (DAWBA) interview [36], which estimates the probability of several psychiatric diagnoses in children and adolescents (based on International Classification of Diseases-10 (ICD-10) and Diagnostic and Statistical Manual of Mental Disorders fourth edition (DSM-IV) criteria). Here, we designated an estimated probability of depression of >50% as a diagnosis for depression, and defined CMDs as an estimated probability of >50% for depression and/or any anxiety disorder (generalised anxiety disorder, panic disorder, agoraphobia, social phobia and specific phobias).

At the 17/18 clinic, depression and anxiety were assessed using a self-administered computerised CIS-R questionnaire [37]. As with DAWBA, CIS-R can be used to assign ICD-10 diagnoses of depression and anxiety disorders [38]. Here, the criteria of mild depression (which included

For peer review only - http://bmjopen.bmj.com/site/about/guidelines.xhtml

moderate and severe depression) was used as a diagnosis of depression, while a diagnosis of CMD was defined as meeting the criteria for mild depression and/or an anxiety disorder (generalised anxiety disorder, mixed anxiety and depression, panic disorders and phobic disorders).

At the other ages (16/17, 18/19, 21/22 and 22/23 questionnaires), depression was assessed using a self-administered Short Mood and Feelings Questionnaire (SMFQ), a 13-item questionnaire assessing depressive symptoms over the past two weeks [39]. Total SMFQ scores range between 0-26, with a score of 12 or more frequently used as a diagnosis of depression [40]. Although there are problems of inaccuracy with using cut-offs from questionnaires as screening tools for depression [41], using ALSPAC data the validity of the SMFQ during childhood and adolescence was found to be high when compared against ICD-10-derived depression diagnoses from CIS-R at age 17/18 [42]. Only participants who answered all 13 SMFQ questions were included in the analyses.

To compare sociodemographic differences between those with and without linked primary care data and to explore whether demographic factors impact rates of identification in primary care records, several variables measured during pregnancy or at birth and known to be predictive of non-response in ALSPAC were utilised [31,32]. These include child sex; maternal age, home ownership status; marital status and parity; parental education levels; and child ethnicity. Additional variables used for aims 2 and 3 are discussed below.

Electronic primary care data

The linked primary care data comprised Read codes V.2 (5 byte), along with associated dates. Read codes relevant to diagnosis, symptoms or treatment (antidepressants, anxiolytics and hypnotics) of depression or anxiety (including phobic disorders) were extracted [10,11]. These were combined to produce three definitions of depression and CMDs (table 2). Based on previous research [10], these were chosen to include the definitions with the lowest sensitivity (‘current diagnosis, treated’), the highest sensitivity (‘current diagnosis or treatment or symptoms’), and an intermediate sensitivity which is also the most straightforward to extract from primary care records (‘current diagnosis’). ‘Current’ diagnoses, symptoms or treatment were defined as being 6 months either side of the age the study child attended the clinic or completed the questionnaire and ‘historical’ as having occurred at least 6 months prior to the age at the clinic/questionnaire. Note that treatment does not include psychological therapies, even though these are the recommended first line of treatment for adolescents, as these therapies are mainly given by specialist secondary mental health services and may not be noted in primary care records. Read codes were used to identify referrals to mental health services. A list of the Read codes used are provided in supplementary table S1.

Table 2: Details of the multiple definitions of ‘depression’ and ‘CMD’ derived from the primary care data.

Definition	Description
**Current diagnosis**	Current diagnosis of depression/CMD
**Current diagnosis, treated**	Current diagnosis of depression/CMD and currently receiving treatment
**Current diagnosis or treatment or symptoms**	Current diagnosis or symptoms or treatment for depression/CMD

For peer review only - http://bmjopen.bmj.com/site/about/guidelines.xhtml

Additional data were extracted to predict identification in primary care records and for the prediction models. These primary care variables may be associated with our outcomes of interest, and included: average annual number of GP consultations and prescriptions at the relevant time point; current and historical somatic and general symptoms (defined in supplementary table S1); referral to mental health services; common chronic health conditions (asthma and eczema); other mental health conditions (eating disorders, ADHD, conduct disorder, autistic spectrum disorder, alcohol and drug abuse, schizophrenia, bipolar disorder and psychosis); family and personal history of depression and mental health issues; self-harm; and smoking status (described in more detail below).

To ensure that only individuals with primary care data at the relevant time points were included, inclusion criteria were: i) having associated linkage data; ii) having primary care data for at least 6 months after their clinic visit or questionnaire completion (based on GP registration dates); and iii) first appearing in the primary care records a minimum of 18 months prior to their clinic visit or questionnaire completion (allowing a 6 month window for 'current' data, plus a whole year previous for 'historical' data).

Statistical Analysis

For each primary care definition and at each time point, sensitivity, specificity and positive and negative predictive values were calculated separately for depression and CMDs (if measured), using the ALSPAC questionnaire data as the reference standard. Exact 95% confidence intervals were derived using binomial probabilities.

We then explored factors associated with identification of CMDs/depression in primary care records for individuals diagnosed in the ALSPAC data. As primary care diagnosis numbers were low, we used the definition with the highest sensitivity ('current diagnosis or symptoms or treatment'). Univariate logistic regression was used to explore whether each covariate was associated with identification. The variables used to predict identification were a combination of ALSPAC and primary care data (for a full list see table S2). These identification analyses were repeated for each timepoint, separately for both depression and CMD (if measured).

Finally, lasso (Least Absolute Selection and Shrinkage Operator) models were used at each time point to assess the combination of variables from primary care data which best predicted depression/CMDs from the ALSPAC data. Lasso models apply a lambda weight which constrains weakly-predictive variables falling below this value to zero, while also shrinking remaining non-zero coefficients towards zero. This results in sparse models which minimise over-fitting, and can subsequently be used for out-of-sample prediction [43,44]. Ten-fold cross-validation was used for all lasso models and visual inspection of the cross-validation plots were performed to assess that the optimal lambda value had been selected.

We randomly split our sample into 60% training and 40% validation samples, and then compared the deviance ratios (a goodness-of-fit statistic comparable to R^2, but for non-linear models) for each to inspect how well the training model performed when predicting depression/CMDs in the validation sample. Logistic lasso models were used with ALSPAC-derived depression or CMDs at each time point as the outcome variable, and all variables in table S3 as predictors.

To assess whether these models, which utilise all the available information held in primary care records, increase model fit relative to just the primary care diagnosis/symptoms/treatment data, we

For peer review only - http://bmjopen.bmj.com/site/about/guidelines.xhtml

compared these models against: i) a prediction model which just contained 'current diagnosis' as a predictor variable; and ii) a prediction model which included 'current diagnosis', 'current symptoms' and 'current treatment' as predictor variables. In- and out-of-sample deviance ratios of these models were compared to assess model fit.

For each time point, from the models utilising all the available primary care data we also calculated the predicted probabilities of receiving a depression or CMD diagnosis (with a threshold of >50% probability to define diagnosis) in the 40% validation sample, and compared sensitivities and specificities derived from these prediction models against the three definitions using the raw primary care data (table 2). All analyses were conducted using Stata v.16.0.

RESULTS

Demographics and Linkage Data Coverage

Table 1 shows numbers with both linkage and ALSPAC data at each time point (the reasons individuals who have ALSPAC data, but do not have linkage data, are provided in table S4). The proportion of unlinked records increases with age, most likely because these individuals left the area as they became adults.

Comparisons between those with ALSPAC data who do and do not have primary care data are presented in tables 3 (for age 15/16 and 22/23 time points) and S5 (for all other time points). There are some differences, particularly in terms of socio-economic position (e.g., less likely to have primary care data if higher parental education levels), but little difference in terms of sex. At later time points, participants with more GCSEs or equivalents are less likely to have primary care data. Few differences in depression/CMD diagnosis are apparent between these two groups. With the exception of CMD/depression diagnoses (which increases with age) differences in demographics across the time points are minimal, although the proportion of females with ALSPAC data does increase over time.

Figure 1 gives the proportions with a current diagnosis of depression/CMD in the primary care data comparing those who did to those who did not complete the ALSPAC clinic or questionnaire. Those with ALSPAC data are more likely to have a current CMD diagnosis, particularly at the later time points. For depression, those with ALSPAC data are slightly more likely to have a primary care diagnosis at ages 21/22 and 22/23 but there are no differences at earlier time points.

For peer review only - http://bmjopen.bmj.com/site/about/guidelines.xhtml

Table 3: Demographics at the age 15/16 clinic and 22/23 questionnaire time points. Of those with ALSPAC data, the table compares those who have primary care data against those who do not. For categorical variables cells are counts and percentages. For continuous variables cells are means and standard deviations. Note also that the denominators vary as the variables come from different data sources, with different levels of completeness. As the demographics are broadly similar across all time points, only the first and last time point are presented here (see table S5 for all other time points).

	Age 15/16 (TF3 clinic) – DAWBA (CMDs and Depression)		Age 22/23 (YPB questionnaire) – SMFQ (Depression)	
	Primary care data (*n*=3663)	No primary care data (*n*=1669)	Primary care data (*n*=1325)	No primary care data (*n*=2571)
**Sex**				
Male	1748 (47.7%)	782 (46.9%)	474 (35.8%)	868 (33.8%)
Female	1915 (52.3%)	887 (53.2%)	851 (64.2%)	1703 (66.2%)
**Maternal age at child's birth**	29.2 (4.6)	29.3 (4.6)	29.2 (4.5)	29.6 (4.4)
**Mother's home ownership status**				
Owned/ Mortgaged	2878 (85.3%)	1292 (83.8%)	998 (84.2%)	2022 (85.5%)
Rented	137 (4.1%)	90 (5.8%)	58 (4.9%)	121 (5.1%)
Council/Housing Association	281 (8.3%)	111 (7.2%)	100 (8.4%)	151 (6.4%)
Other	79 (2.3%)	48 (3.1%)	30 (2.5%)	70 (3%)
**Mother's marital status**				
Never married	460 (13.5%)	211 (13.6%)	152 (12.6%)	262 (11%)
Single/Divorced	147 (4.3%)	83 (5.3%)	50 (4.2%)	105 (4.4%)
First marriage	2607 (76.5%)	1149 (74%)	918 (76.2%)	1851 (77.7%)
2nd/3rd marriage	194 (5.7%)	110 (7.1%)	85 (7.1%)	164 (6.9%)
**Mother's parity**				
0	1623 (48.1%)	787 (51.3%)	560 (47%)	1155 (49.1%)
1	1201 (35.6%)	507 (33.1%)	413 (34.7%)	815 (34.7%)
2 or more	554 (16.4%)	240 (15.7%)	218 (18.3%)	381 (16.2%)
**Mother's highest education level**				
O level/lower	1884 (55.2%)	765 (49.3%)	748 (62.8%)	1071 (45.5%)
A level	920 (27.3%)	476 (30.7%)	280 (23.5%)	770 (30.6%)
Degree	565 (16.8%)	312 (20.1%)	164 (13.8%)	562 (23.9%)
**Father's highest education level**				
O level/lower	1428 (46.2%)	559 (39.3%)	550 (50.5%)	795 (35.9%)
A level	947 (30.6%)	437 (30.6%)	348 (32%)	653 (29.5%)

For peer review only - http://bmjopen.bmj.com/site/about/guidelines.xhtml

**Degree**	716 (23.2%)	429 (30.1%)	191 (17.5%)	767 (34.6%)
**Child ethnicity**				
**White**	3179 (95.9%)	1466 (95.8%)	1138 (96.8%)	2237 (96.2%)
**Non-white**	137 (4.1%)	65 (4.3%)	38 (3.2%)	88 (3.8%)
**# GCSEs (or equivalents)**	7.3 (3.6)	7.5 (3.6)	7.3 (3.5)	8.3 (3.2)
**ALSPAC depression diagnosis**				
**No**	3606 (98.4%)	1638 (98.1%)	1103 (83.2%)	2180 (84.6%)
**Yes**	57 (1.6%)	31 (1.9%)	223 (16.8%)	398 (14.5%)
**ALSPAC common mental disorder (CMD) diagnosis**				
**No**	3540 (96.6%)	1622 (97.2%)	-	-
**Yes**	123 (3.4%)	47 (2.8%)	-	-

DAWBA: Development and Well-Being Assessment; *CMDs:* Common mental disorders; *SMFQ*: Short Mood and Feelings Questionnaire.

For peer review only - http://bmjopen.bmj.com/site/about/guidelines.xhtml

Sensitivity, Specificity and Predictive Values

We focused first on the age 17/18 clinic data (table 4), the results of which were broadly consistent with previous ALSPAC analyses [10]. At this age, 243/3,084 participants (7.9%) were diagnosed as depressed using the CIS-R data, while 455 (14.8%) met the criteria for diagnosis of CMD. Using the various primary care definitions, the number of individuals diagnosed as depressed ranged from a minimum of 20 (0.7%) using a definition of 'current diagnosis, treated', to a maximum of 122 (4%) using a definition of 'current diagnosis or symptoms or treatment'. Thus, the sensitivity of each primary care definition was low, ranging from 3.7% to 24.3%. Specificity was higher (all > 97.8%), as was the negative predictive values (NPVs; all > 92%), while positive predictive values (PPVs) ranged between 45% and 58.4%. For the primary care CMD data, numbers diagnosed ranged from a minimum of 29 (0.9%) to a maximum of 171 (5.5%). CMD sensitivity (range: 3.5-19.1%) and specificity (all > 96.8%) were marginally lower compared to depression at this age, while each PPV was slightly higher (range: 47.4-55.2%) and NPV lower (range: 85.6-87.4%).

Similar results were found for the age 15/16 clinic using the DAWBA measure (table S7), albeit with fewer depression and CMD diagnoses and lower sensitivities. The comparison between SMFQ questionnaire data and primary care data at ages 16/17, 18/19, 21/22 and 22/23 are displayed in supplementary tables S8 to S11, and were similar to those using DAWBA (age 15/16 clinic) and CIS-R (age 17/18 clinic), with relatively high specificity but low sensitivity for all primary care definitions of depression. Sensitivity increased with age, while specificity decreased (figure 2).

Identification of CMDs/Depression Cases in Primary Care Records

The results of the primary care records identification analyses are presented in full in table S12 (giving odds ratios and 95% confidence intervals for all analyses) and figure S1 (providing a graphical summary of key results over each time point). There are few consistent associations of sociodemographic factors (parental education, child sex, child education, etc.) with being identified as a case in the primary care records. Primary care case identification was more likely in participants with greater symptom severity. Some primary care covariates (e.g., smoking status, eating disorder and other mental health issues) were associated with higher rates of primary care case identification at younger ages, but had weaker associations at later ages. Others (somatic and general symptoms, higher consultation/prescription rates, referrals to mental health services and self-harm status) were consistently associated with higher rates of primary care case identification. Due to the low numbers diagnosed as having CMD or depression at the age 15/16 clinic, both from the DAWBA assessment and from primary care records, results from this time point should be treated with caution.

For peer review only

For peer review only - http://bmjopen.bmj.com/site/about/guidelines.xhtml

Table 4: Depression and CMD diagnoses based on the CIS-R (clinical interview schedule – revised) data from the age 17/18 TF4 clinic against various definitions derived from the primary care data at this age (*n*=3,084). This table also includes sensitivities, specificities, positive predictive values (PPV) and negative predictive values (NPV) for the depression and common mental disorder (CMD) diagnoses based on the CIS-R data from this clinic. In these analyses we are treating the ALSPAC data as the reference standard.

		CIS-R Depression			CIS-R CMD		
**Primary care definition**		**No**	**Yes**	**Total**	**No**	**Yes**	**Total**
**Current diagnosis**	No	2,825	229	3,054	2,599	428	3,027
	Yes	16	14	30	30	27	57
**Sensitivity**		5.8% (3.2; 9.5)			5.9% (3.9; 8.5)		
**Specificity**		99.4% (99.1; 99.7)			98.9% (98.4; 99.2)		
**Positive Predictive Value**		46.7% (28.3; 65.7)			47.4% (34; 61)		
**Negative Predictive Value**		92.5% (91.5; 93.4)			85.9% (84.6; 87.1)		
		**No**	**Yes**	**Total**	**No**	**Yes**	**Total**
**Current diagnosis, treated**	No	2,830	234	3,064	2,616	439	3,055
	Yes	11	9	20	13	16	29
**Sensitivity**		3.7% (1.7; 6.9)			3.5% (2; 5.6)		
**Specificity**		99.6% (99.3; 99.8)			99.5% (99.2; 99.7)		
**Positive Predictive Value**		45% (23.1; 68.5)			55.2% (35.7; 73.6)		
**Negative Predictive Value**		92.4% (91.4; 93.3)			85.6% (84.3; 86.9)		
		**No**	**Yes**	**Total**	**No**	**Yes**	**Total**
**Current diagnosis or symptoms or treatment**	No	2,778	184	2,962	2,545	368	2,913
	Yes	63	59	122	84	87	171
**Sensitivity**		24.3% (19; 30.2)			19.1% (15.6; 23)		
**Specificity**		97.8% (97.2; 98.3)			96.8% (96.1; 97.4)		
**Positive Predictive Value**		48.4% (39.2; 57.6)			50.9% (43.1; 58.6)		
**Negative Predictive Value**		93.8% (92.9; 94.6)			87.4% (86.1; 88.6)		

Predicting ALSPAC CMDs/Depression from Primary Care Records

The in-sample deviance ratios, fitted on the 60% training sample, and the out-of-sample deviance ratios, fitted on the 40% validation sample, for each time point are displayed in table 5. In general, in-sample deviance ratios are quite low (8.3 to 14.6%). Out-of-sample deviance ratios are lower (-1.3 to 12.6%) but do increase with age. The penalised coefficients from these prediction models are presented in table S13, with full models to estimate predicted probabilities given in table S14. Many factors from the primary care data consistently predicted ALSPAC CMD/depression diagnoses across many time points, including: being female, a history of self-harm, number of GP consultations, referral to mental health services and historical and/or current depression diagnoses/symptoms/treatment. Several associations were time point specific, occurring in only one or two models (e.g., smoking at TF4 depression and CCS, eczema for TF4 depression, conduct disorder at CCS, etc.). These coefficients should not be interpreted causally, especially is there is high collinearity between variables (as is likely to be present here given that many variables measure similar constructs).

For peer review only - http://bmjopen.bmj.com/site/about/guidelines.xhtml

Table 5: In-sample and out-of-sample deviance ratios predicting depression and common mental disorders (CMDs) for each time point using all the available primary care data. Deviance ratios are taken from logistic cross-validation lasso prediction models.

	**Age 15/16 TF3 clinic (*n* = 3,663)**		**Age 16/17 CCS quest. (*n* = 3,213)**	**Age 17/18 TF4 clinic (*n* = 3,084)**		**Age 18/19 CCT quest. (*n* = 1,982)**	**Age 21/22 YPA quest. (*n* = 1,298)**	**Age 22/23 YPB quest. (*n* = 1,325)**
	Dep.	CMD	Dep.	Dep.	CMD	Dep.	Dep.	Dep.
**In-sample deviance ratio (60% training sample)**	9.9%	8.4%	8.3%	14.6%	9.2%	9%	11.7%	13.4%
**Out-of-sample deviance ratio (40% validation sample)**	-1.3%	2.9%	4.3%	7.8%	7.8%	8%	12.6%	9.1%

For all time points other than age 15/16 clinic depression, the ‘full’ prediction model (based on the set of all primary care variables; table S3) performed better than both the ‘diagnosis only’ and ‘diagnosis/symptoms/ treatment’ models for both in-sample and out-of-sample prediction (table S15).

Sensitivities from these prediction models were marginally higher than for definitions of ‘current diagnosis’ and ‘current diagnosis with treatment’, but lower than the ‘current diagnosis or treatment or symptoms’ sensitivities. However, the specificities of the prediction models were greater than the ‘current diagnosis or treatment or symptoms’ definition, and on par with the stricter definitions based on ‘current diagnosis’ or ‘current diagnosis with treatment’ (table S16). These prediction models therefore appear to more accurately detect cases of depression/CMD compared to these more stringent definitions from the primary care records, while also avoiding many of the false negatives associated with broader definitions (such as ‘current diagnosis or treatment of symptoms’). However, sensitivities from these prediction models are still rather low, ranging between 3.5% and 16.3% (all specificities are >98%).

DISCUSSION

This study compared primary care data against validated measures of CMDs at multiple time points during adolescence and young adulthood. Taking ALSPAC data as the reference standard, our results demonstrate that, regardless of how CMDs are defined from primary care records, sensitivities are low across all ages (range: 1.6 to 34%). However, detection of CMDs in primary care records does improve with age. Specificities were high, with most above 95%. This suggests that the primary care data is likely to contain many ‘false negatives’ but few ‘false positives’, as documented previously [12].

This study also explored the factors predicting identification of “cases” (as identified in ALSPAC data) in primary care data. Consistent with previous research [16,22], the strongest predictor was symptom severity, with individuals displaying more severe symptoms increasingly likely to be correctly classified. A history of CMDs, as well as increased rates of other mental health issues, somatic or general symptoms and engagement with primary care services (consultation and

For peer review only - http://bmjopen.bmj.com/site/about/guidelines.xhtml

prescription rates), also predicted greater primary care identification rates. Many adolescents receive mental health care via specialised secondary care services, rather than through their GP, and this is reflected in referrals to secondary mental health services also being associated with higher identification rates. Unlike for the wider adult population, we found little evidence that sociodemographic factors were consistently associated with case identification in primary care records for adolescents and young adults [25,45].

Finally, this paper also presented a series of prediction models, which can be used by epidemiologists with access only to primary care data to predict CMDs in individuals who may not be formally diagnosed by a GP. Although the variance explained by these models is quite low, these models demonstrate that the inclusion of additional covariates from primary care records improved model fit, relative to models that contained only current diagnosis, symptoms or treatment. Out-of-sample prediction rates increased with age, suggesting that these models better predict depression/CMDs in young adulthood compared to adolescence. This is perhaps not surprising, given that rates of diagnoses from primary care data are low in adolescence and increase with age. However, comparison of the predicted sensitivities and specificities from these prediction models indicates that the improvement in detection of depression/CMDs relative to the primary care record data based on diagnosis, symptoms and treatment is minimal.

Strengths and Limitations

A strength of this study is that it uses established methods to systematically define CMDs from primary care records [10,11], allowing cross-study comparisons. This study uses a larger sample than a previous study using ALSPAC adolescent data [10], and extends the age range assessed to adolescence through to early adulthood. This permits a broader view of how both ALSPAC-derived and primary care-derived CMD rates change with age, how sensitivities and specificities vary over the transition to adulthood, and how prediction models alter over this developmental stage. A further strength is that this study also uses a large, deeply-phenotyped cohort, with depression and CMD measured at multiple time points using validated instruments.

This study has several limitations. The primary care data coding used may miss crucial information: possible diagnoses and symptoms may be noted within the 'free text' of routine electronic records [12], which are generally not available for research purposes [46]. The primary care data only records pharmacological treatments prescribed by the GP, rather than psychological treatments provided by secondary care services. As the first line of treatment for adolescents is often psychological therapies, especially for mild depression [47]), this may partially explain the lower sensitivities at younger ages. Although we included CAMHS referrals in our identification analyses and prediction models, this is still likely to underestimate the true prevalence of adolescent CMDs as only around one-third of children with a mental health disorder are referred to CAMHS [21]. Further, fewer than half of referrals to CAMHS in the UK are from a GP [48], with school nurses, self-referrals and other routes to CAMHS possible.

A second limitation is that the linkage is primarily Bristol-based. As the cohort reaches adulthood they are more likely to move away from Bristol; as such, the proportion with linkage data drops from approximately two-thirds before age 18 to roughly one-third after this age. In addition to the resulting loss of statistical power and precision, there is also the potential for selection bias if those with linkage data systematically differ from those without [49,50]. At each time point, of those with ALSPAC data there are differences between those with and without linked primary care data in

For peer review only - http://bmjopen.bmj.com/site/about/guidelines.xhtml

terms of socioeconomic position (e.g., higher maternal education levels are associated with lower probability of having linkage data). Although differences in ALSPAC-derived CMDs appear minimal comparing those with vs without primary care data (table 3), it is possible that primary care data may differ between these groups. This may limit the generalisability of our prediction models; for example, compared to the whole ALSPAC cohort our sample with primary care data is biased towards those with a lower socioeconomic position, who may be less likely to attend GP appointments [51]. However, as in the wider ALSPAC cohort respondents tend to be from higher socioeconomic strata [32], the impact of linkage data biased towards lower-SEP (socioeconomic position) individuals on generalisability is uncertain. Comparing the primary care-derived CMD status of those with and without ALSPAC data we observe few differences in terms of depression or CMDs at younger ages but, in adulthood, CMDs (although less so for depression) appear more prevalent among those with ALSPAC data (figure 1). One possible interpretation of this is that it reflects the demographics of ALSPAC respondents, as being female is associated with continued ALSPAC participation [32], and females are at greater risk of CMDs [52,53]. When adjusting for sex these effects were somewhat attenuated, although participants with ALSPAC data at the 21/22 and 22/23 questionnaire time points were still more likely to have a primary care-derived CMD (table S17). Inclusion of parental education (a proxy for SEP), which may also predict both continued ALSPAC participation and mental health, did not further diminish this effect. The selection pressures associated with having continued primary care linkage data in ALSPAC are likely to be complex and require further investigation to assess the potential for selection bias when using this data.

A related limitation is that as the research is specifically Bristol-based, generalisability to other populations, both in the UK and elsewhere, should be made with caution. For instance, the ALSPAC cohort is not representative of the UK national population, as ALSPAC contains a greater proportion of white and higher SEP individuals [32], which is likely to shape health-seeking behaviour and GP engagement rates [24,25]. A further issue regarding generalisability is that the data in adolescence was collected between 2006 and 2011. Given the large shift in societal values towards increased visibility, awareness and understanding of mental health issues over the past few years, this may impact both GP decision-making and adolescents' health-seeking behaviour, potentially affecting diagnosis rates in this age group. Additional research is necessary to explore this among existing adolescents. As such, these models should be calibrated before use in other areas or calendar times.

A third limitation is the small numbers of individuals with CMD/depression in ALSPAC, especially at younger ages (and particularly the age 15/16 clinic data). This may explain why we failed to detect consistent sociodemographic differences in case identification, contrary to previous research with larger samples [16,25,45]. Larger studies are required to explore sociodemographic associations with identification in primary care records in greater detail, which – if present – are likely to be weaker than the effects of symptom severity [16,22]. In addition to the relatively small sample size, one potential reason for the lack of SEP gradient could be that SEP is based on parental SEP at the time of the study child's birth. Although parental SEP and child health outcomes are frequently correlated, this association is strongest in early childhood and tends to weaken with age [24]. Assessing the individual's SEP directly, particularly in early adulthood, may reveal these health inequalities.

A further limitation is that we have taken the ALSPAC data as the 'reference standard'. These measures may over-diagnose the presence of CMDs, especially in 'borderline' cases with less severe symptoms who may not visit their GP, thus increasing the number of false positives in the ALSPAC data. Although all of the instruments used in ALSPAC have been validated and are routinely used to screen for depression and CMDs [36,37,39,42], previous studies have demonstrated that these

For peer review only - http://bmjopen.bmj.com/site/about/guidelines.xhtml

questionnaire-based tools can provide quite divergent diagnoses of mental health conditions compared to standard clinical interviews (e.g., CIS-R; [38]). Additionally, apparent false negatives may also appear in the ALSPAC data if individuals are successfully receiving treatment to alleviate their CMD symptoms; in these cases, individuals would be diagnosed as having CMD via primary care records, but not via ALSPAC data.

Implications and Recommendations

Consistent with previous research [12], this study has demonstrated that the rate of false negatives for CMDs in adolescents and young adults in routine primary care data is high. Thus, additional sources of information need to be utilised when working with routine health data. As fewer than half of referrals to CAMHS are from GPs [48], using linkage data from CAMHS and other secondary mental health care services would likely increase detection rates. This would appear particularly important for adolescents, as the sensitivities at this age are much lower than in early adulthood. However, as CAMHS is over-subscribed, often only severe cases are accepted, potentially biasing these sources towards those with more severe CMD symptoms. Additionally, even in early adulthood sensitivities are still rather low (maximum 34% at age 21/22), suggesting that additional information is required to correctly identify CMDs in linkage data. One potential source of information is from the free-text fields in primary care records, which are not usually made available for research purposes [12]. However, although evidence suggests that using free text data can improve detection of medical conditions more generally [54], the current evidence for CMDs – albeit limited to a small number of studies – suggests their inclusion only marginally improves detection rates [12].

Conclusion

We have demonstrated how routine electronic primary care data can be used with cohort study data to estimate the size of the 'clinical iceberg' of undetected CMDs in primary care data throughout adolescence and early adulthood, and to describe the characteristics of those less likely to be identified as cases in primary care records. Although overall sensitivities were low, both sources of data accurately predicted individuals with more severe CMD symptoms. The number of individuals diagnosed as having a CMD, and the correspondence between ALSPAC and primary care data, increased with age. Additional sources of data – e.g., from secondary care services such as CAMHS, or from free text fields – might be required to determine CMD prevalence more accurately, particularly in adolescence. Development of further prediction models may improve estimation of prevalence of CMDs from primary care records and help target interventions to individuals with CMDs who would otherwise not be identified as cases in primary care records.

For peer review only - http://bmjopen.bmj.com/site/about/guidelines.xhtml

Acknowledgements

We are extremely grateful to all the families who took part in this study, the midwives for their help in recruiting them, and the whole ALSPAC team, which includes interviewers, computer and laboratory technicians, clerical workers, research scientists, volunteers, managers, receptionists and nurses.

Author Contributions

RPC, KT, SLP, KW, GS and JD conceived and designed the study. RPC processed the primary care data. DS performed the statistical analyses and drafted the manuscript. RPC, KT, SLP, KW, GS and JD contributed to the interpretation of the results. All authors commented on the draft, and have read and approved the final version of the manuscript.

Funding

This work was funded by the Medical Research Council (MRC grant number: MC_PC_17210). The UK Medical Research Council and Wellcome (Grant ref: 217065/Z/19/Z) and the University of Bristol provide core support for ALSPAC. A comprehensive list of grants funding is available on the ALSPAC website http://www.bristol.ac.uk/alspac/external/documents/grant-acknowledgements.pdf); collection of ALSPAC CMD data was funded by the NIH (grant references 5R01MH073842-04 and PD301198-SC101645; for the TF3 DAWBA and YPB MFQ data), Wellcome Trust (grant reference 08426812/Z/07/Z; for the TF4 CIS-R data), and a joint Wellcome Trust and MRC grant (grant reference 092731; for the CCS, CCT and YPA SMFQ data). This publication is the work of the authors and Daniel Smith and Rosie Cornish will serve as guarantors for the contents of this paper.

Competing interests

None declared.

Data availability statement

Information about access to ALSPAC data is given on the ALSPAC website (http://www.bristol.ac.uk/alspac/researchers/access/).

Ethics approval

Ethical approval for the study was obtained from the ALSPAC Ethics and Law Committee and the Local Research Ethics Committees (NHS Haydock REC: 10/H1010/70). Informed consent for the use of data collected via questionnaires and clinics was obtained from participants following the recommendations of the ALSPAC Ethics and Law Committee at the time.

Supplementary material

Supplementary material supporting this manuscript is available as an online supplement.

For peer review only

References

1 Ferrari AJ, Charlson FJ, Norman RE, *et al.* Burden of Depressive Disorders by Country, Sex, Age, and Year: Findings from the Global Burden of Disease Study 2010. *PLoS Med* 2013;**10**. doi:10.1371/journal.pmed.1001547

2 Pearson RM, Carnegie RE, Cree C, *et al.* Prevalence of Prenatal Depression Symptoms Among 2 Generations of Pregnant Mothers: The Avon Longitudinal Study of Parents and Children. *JAMA Netw open* 2018;**1**:e180725. doi:10.1001/jamanetworkopen.2018.0725

3 Collishaw S, Maughan B, Natarajan L, *et al.* Trends in adolescent emotional problems in England: A comparison of two national cohorts twenty years apart. *J Child Psychol Psychiatry Allied Discip* 2010;**51**:885–94. doi:10.1111/j.1469-7610.2010.02252.x

4 Lewinsohn PM, Clarke GN, Seeley JR, *et al.* Major Depression in Community Adolescents: Age at Onset, Episode Duration, and Time to Recurrence. J. Am. Acad. Child Adolesc. Psychiatry. 1994;**33**:809–18. doi:10.1097/00004583-199407000-00006

5 Patel V, Flisher AJ, Hetrick S, *et al.* Mental health of young people: a global public-health challenge. *Lancet* 2007;**369**:1302–13. doi:10.1016/S0140-6736(07)60368-7

6 Green H, McGinnity A, Meltzer H, *et al.* Mental health of children and young people in Great Britain, 2004. 2005. doi:10.1037/e557702010-001

7 McManus S, Meltzer H, Brugha T, *et al.* Adult psychiatric morbidity in England, 2007: Results of a household survey. 2009. doi:10.13140/2.1.1563.5205

8 Bhatia SK, Bhatia SC. Childhood and adolescent depression. *Am Fam Physician* 2007;**75**:73–80. doi:10.1192/bjp.153.4.476

9 Harvey SB, Henderson M, Lelliott P, *et al.* Mental health and employment: Much work still to be done. *Br J Psychiatry* 2009;**194**:201–3. doi:10.1192/bjp.bp.108.055111

10 Cornish R, John A, Boyd A, *et al.* Defining adolescent common mental disorders using electronic primary care data: A comparison with outcomes measured using the CIS-R. *BMJ Open* 2016;**6**. doi:10.1136/bmjopen-2016-013167

11 John A, McGregor J, Fone D, *et al.* Case-finding for common mental disorders of anxiety and depression in primary care: An external validation of routinely collected data. *BMC Med Inform Decis Mak* 2016;**16**:1–10. doi:10.1186/s12911-016-0274-7

12 Larvin H, Peckham E, Prady SL. Case-finding for common mental disorders in primary care using routinely collected data: a systematic review. *Soc Psychiatry Psychiatr Epidemiol* 2019;**54**:1161–75. doi:10.1007/s00127-019-01744-4

13 Davis KAS, Sudlow CLM, Hotopf M. Can mental health diagnoses in administrative data be used for research? A systematic review of the accuracy of routinely collected diagnoses. *BMC Psychiatry* 2016;**16**:1–11. doi:10.1186/s12888-016-0963-x

14 Coyne JC, Schwenk TL, Fechner-Bates S. Nondetection of depression by primary care physicians reconsidered. *Gen Hosp Psychiatry* 1995;**17**:3–12. doi:10.1016/0163-8343(94)00056-J

15 Cepoiu M, McCusker J, Cole MG, *et al.* Recognition of depression by non-psychiatric physicians - A systematic literature review and meta-analysis. *J Gen Intern Med* 2008;**23**:25–36. doi:10.1007/s11606-007-0428-5

16 Bebbington P, Meltzer H, Brugha T, *et al.* Unequal access and unmet need: Neurotic disorders

For peer review only - http://bmjopen.bmj.com/site/about/guidelines.xhtml

and the use of primary care services. *Psychol Med* 2000;**30**:1359–67. doi:10.1080/0954026021000046029

17 Rait G, Walters K, Griffin M, *et al.* Recent trends in the incidence of recorded depression in primary care. *Br J Psychiatry* 2009;**195**:520–4. doi:10.1192/bjp.bp.108.058636

18 Wijlaars LPMM, Nazareth I, Petersen I. Trends in depression and antidepressant prescribing in children and adolescents: A cohort study in the health improvement network (THIN). *PLoS One* 2012;**7**. doi:10.1371/journal.pone.0033181

19 Iliffe S, Williams G, Fernandez V, *et al.* General practitioners' understanding of depression in young people: Qualitative study. *Prim Heal Care Res Dev* 2008;**9**:269–79. doi:10.1017/S1463423608000868

20 O'Brien D, Harvey K, Howse J, *et al.* Barriers to managing child and adolescent mental health problems: A systematic review of primary care practitioners' perceptions. *Br J Gen Pract* 2016;**66**:e693–707. doi:10.3399/bjgp16X687061

21 Sayal K. Annotation: Pathways to care for children with mental health problems. *J Child Psychol Psychiatry Allied Discip* 2006;**47**:649–59. doi:10.1111/j.1469-7610.2005.01543.x

22 Henderson JG, Pollard CA, Jacobi KA, *et al.* Help-seeking patterns of community residents with depressive symptoms. *J Affect Disord* 1992;**26**:157–62.

23 Fryers T, Melzer D, Jenkins R. Social inequalities and the common mental disorders - A systematic review of the evidence. *Soc Psychiatry Psychiatr Epidemiol* 2003;**38**:229–37. doi:10.1007/s00127-003-0627-2

24 Adler NE, Stewart J. Health disparities across the lifespan: Meaning, methods, and mechanisms. *Ann N Y Acad Sci* 2010;**1186**:5–23. doi:10.1111/j.1749-6632.2009.05337.x

25 Prady SL, Pickett KE, Petherick ES, *et al.* Evaluation of ethnic disparities in detection of depression and anxiety in primary care during the maternal period: Combined analysis of routine and cohort data. *Br J Psychiatry* 2016;**208**:453–61. doi:10.1192/bjp.bp.114.158832

26 Bebbington P, Brugha T, Meltzer H, *et al.* Neurotic disorders and the receipt of psychiatric treatment. *Psychol Med* 2000;**30**:1369–76. doi:10.1080/0954026021000046010

27 Prady SL, Pickett KE, Gilbody S, *et al.* Variation and ethnic inequalities in treatment of common mental disorders before, during and after pregnancy: Combined analysis of routine and research data in the Born in Bradford cohort. *BMC Psychiatry* 2016;**16**:1–13. doi:10.1186/s12888-016-0805-x

28 Fernandez A, Salvador-Carulla L, Choi I, *et al.* Development and validation of a prediction algorithm for the onset of common mental disorders in a working population. *Aust N Z J Psychiatry* 2018;**52**:47–58. doi:10.1177/0004867417704506

29 Cornish RP, Macleod J, Boyd A, *et al.* Factors associated with participation over time in the Avon Longitudinal Study of Parents and Children: a study using linked education and primary care data. *Int J Epidemiol* 2020;:1–10. doi:10.1093/ije/dyaa192

30 Northstone K, Lewcock M, Groom A, *et al.* The Avon Longitudinal Study of Parents and Children (ALSPAC): an update on the enrolled sample of index children in 2019 [version 1; peer review: 2 approved]. *Wellcome Open Res* 2019;**4**:1–10. doi:10.12688/wellcomeopenres.15132.1

31 Fraser A, Macdonald-wallis C, Tilling K, *et al.* Cohort profile: The avon longitudinal study of parents and children: ALSPAC mothers cohort. *Int J Epidemiol* 2013;**42**:97–110.

For peer review only - http://bmjopen.bmj.com/site/about/guidelines.xhtml

doi:10.1093/ije/dys066

32 Boyd A, Golding J, Macleod J, *et al.* Cohort profile: The 'Children of the 90s'-The index offspring of the avon longitudinal study of parents and children. *Int J Epidemiol* 2013;**42**:111–27. doi:10.1093/ije/dys064

33 Harris PA, Taylor R, Thielke R, *et al.* Research electronic data capture (REDCap)-A metadata-driven methodology and workflow process for providing translational research informatics support. *J Biomed Inform* 2009;**42**:377–81. doi:10.1016/j.jbi.2008.08.010

34 von Elm E, Altman DG, Egger M, *et al.* The Strengthening the Reporting of Observational Studies in Epidemiology (STROBE) Statement: guidelines for reporting observational studies. doi:10.4324/9781315883595-19

35 Boyd A, Tilling K, Cornish R, *et al.* Professionally designed information materials and telephone reminders improved consent response rates: Evidence from an RCT nested within a cohort study. *J Clin Epidemiol* 2015;**68**:877–87. doi:10.1016/j.jclinepi.2015.03.014

36 Goodman R, Ford T, Richards H, *et al.* The Development and Well-Being Assessment: Description and Initial Validation of an Integrated Assessment of Child and Adolescent Psychopathology. *J Child Psychol Psychiatry* 2000;**41**:645–55. doi:10.1111/j.1469-7610.2000.tb02345.x

37 Lewis G, Pelosi AJ, Araya R, *et al.* Measuring psychiatric disorder in the community: A standardized assessment for use by lay interviewers. *Psychol Med* 1992;**22**:465–86. doi:10.1017/S0033291700030415

38 Brugha TS, Bebbington PE, Jenkins R, *et al.* Cross validation of a general population survey diagnostic interview: A comparison of CIS-R with SCAN ICD-10 diagnostic categories. *Psychol Med* 1999;**29**:1029–42. doi:10.1017/S0033291799008892

39 Angold A, Costello EJ, Messer SC, *et al.* Development of a short questionnaire for use in epidemiological studies of depression in children and adolescents. *Int J Methods Psychiatr Res* 1995;**5**:237–49.

40 Thabrew H, Stasiak K, Bavin LM, *et al.* Validation of the Mood and Feelings Questionnaire (MFQ) and Short Mood and Feelings Questionnaire (SMFQ) in New Zealand help-seeking adolescents. *Int J Methods Psychiatr Res* 2018;**27**:1–9. doi:10.1002/mpr.1610

41 Roseman M, Kloda LA, Saadat N, *et al.* Accuracy of Depression Screening Tools to Detect Major Depression in Children and Adolescents: A Systematic Review. *Can J Psychiatry* 2016;**61**:746–57. doi:10.1177/0706743716651833

42 Turner N, Joinson C, Peters TJ, *et al.* Validity of the Short Mood and feelings questionnaire in late adolescence. *Psychol Assess* 2014;**26**:752–62. doi:10.1037/a0036572

43 Hastie T, Tibshirani R, Wainwright M. *Statistical Learning with Sparsity: The Lasso and Generalizations*. CRC Press 2015.

44 Tibshirani R. Regression Shrinkage and Selection via the Lasso. *J R Stat Soc Ser B Stat Methodol* 1996;**58**:267–88.

45 McManus S, Bebbington P, Jenkins R, *et al.* Mental health and wellbeing in England: Adult Psychiatric Morbidity Survey 2014. Leeds: 2016. doi:10.1079/9781786393401.0000

46 Price SJ, Stapley SA, Shephard E, *et al.* Is omission of free text records a possible source of data loss and bias in Clinical Practice Research Datalink studies? A case-control study. *BMJ Open* 2016;**6**:1–7. doi:10.1136/bmjopen-2016-011664

For peer review only - http://bmjopen.bmj.com/site/about/guidelines.xhtml

47 NICE. Depression in children and young people: Identification and management. 2019. doi:10.1211/CP.2018.20204575

48 Children's Commissioner for England. Lightning Review: Access to Child and Adolescent Mental Health Services, May 2016. London, UK: 2016. https://www.childrenscommissioner.gov.uk/publication/lightning-review-access-to-child-and-adolescent-mental-health-services/ (accessed 3/3/2020)

49 Hernán M, Robins J. *Causal Inference: What If*. Boca Raton: : Chapman & Hall/CRC Press 2020.

50 Munafò MR, Tilling K, Taylor AE, *et al.* Collider scope: When selection bias can substantially influence observed associations. *Int J Epidemiol* 2018;**47**:226–35. doi:10.1093/ije/dyx206

51 Ellis DA, McQueenie R, McConnachie A, *et al.* Demographic and practice factors predicting repeated non-attendance in primary care: a national retrospective cohort analysis. *Lancet Public Heal* 2017;**2**:e551–9. doi:10.1016/S2468-2667(17)30217-7

52 Leach LS, Christensen H, Mackinnon AJ, *et al.* Gender differences in depression and anxiety across the adult lifespan: The role of psychosocial mediators. *Soc Psychiatry Psychiatr Epidemiol* 2008;**43**:983–98. doi:10.1007/s00127-008-0388-z

53 McLean CP, Asnaani A, Litz BT, *et al.* Gender differences in anxiety disorders: Prevalence, course of illness, comorbidity and burden of illness. *J Psychiatr Res* 2011;**45**:1027–35. doi:10.1016/j.jpsychires.2011.03.006

54 Ford E, Carroll JA, Smith HE, *et al.* Extracting information from the text of electronic medical records to improve case detection: A systematic review. *J Am Med Informatics Assoc* 2016;**23**:1007–15. doi:10.1093/jamia/ocv180

For peer review only

For peer review only - http://bmjopen.bmj.com/site/about/guidelines.xhtml

Figure 1: Comparing primary care common mental disorder (CMD) and depression rates in participants with vs without ALSPAC data at each time point. For participants who did not attend the clinic/complete the questionnaire, the age to define a 'current' diagnosis was based on +/- 6 months from the average age each clinic/questionnaire was completed. Individuals who have primary care data and attended/completed the clinic/questionnaire, but do not have ALSPAC-derived depression/CMD data (as this session was not completed for whatever reason), are not included in the figure below. Full details of these numbers, and the data for this figure, are provided in table S6.

Figure 2: Sensitivity, specificity and positive/negative predicted values for depression (black) and common mental disorders (CMDs; red) over each of the time points studies. Results are based on the definition 'current diagnosis, symptoms or treatment' to determine cases in primary care records, treating the ALSPAC data as the reference standard. Note that CMDs were only assessed at the age 15/16 and 17/18 clinics.

For peer review only

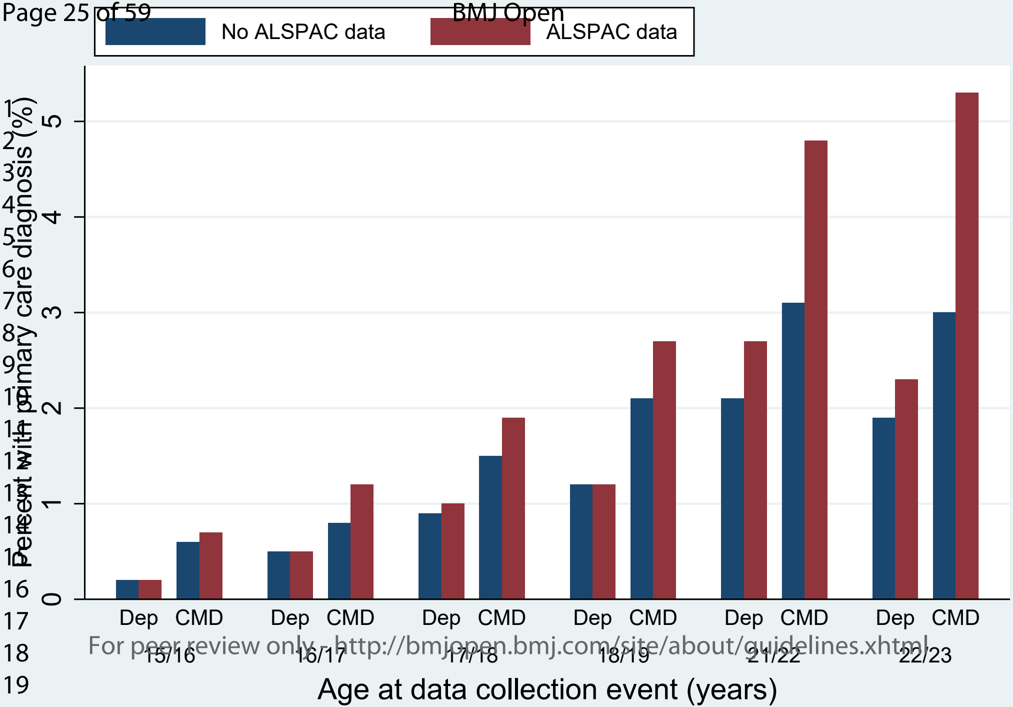

For peer review only - http://bmjopen.bmj.com/site/about/guidelines.xhtml

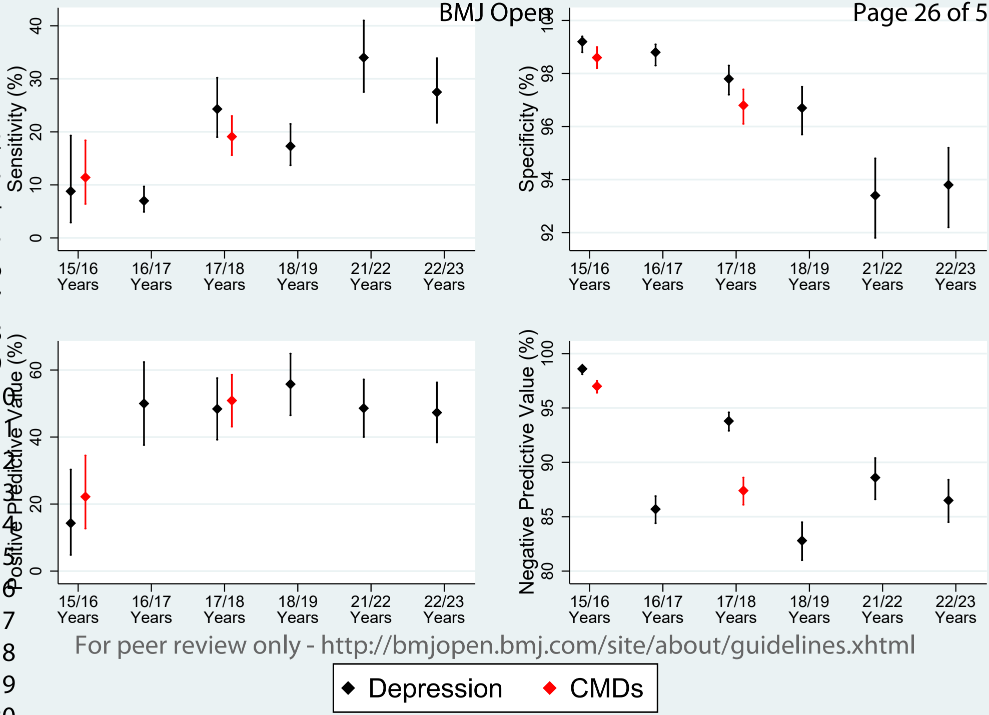
Sensitivity (%)
0
10
20
30
40
Specificity (%)
92
94
96
98
100
Positive Predictive Value (%)
0
20
40
60
Negative Predictive Value (%)
80
85
90
95
100
15/16 Years
16/17 Years
17/18 Years
18/19 Years
21/22 Years
22/23 Years
Depression
CMDs

Supplementary information for 'Assessing and predicting adolescent and early adulthood common mental disorders in the ALSPAC cohort using electronic primary care data'

Linkage to primary care data

The Secure Anonymised Information Linkage (SAIL) project has established a system which allows individual-level data from multiple sources – including electronic primary care data – to be linked together and analysed securely [1]. In collaboration with SAIL and GP software system providers, the Project to Enhance ALSPAC through Record Linkage (PEARL) developed two methods to extract primary care data. The first was a pilot extraction in 2012 using a sample of study participants based on an 'opt-in' consent principle, from which 2,806 participants consented to having their primary care records accessed (see [2] for further details). The second method was the main data extraction, where all ALSPAC participants with known addresses were sent fair processing materials and allowed to 'opt-out' of having their primary care records extracted, if they did not consent to this. Extraction of the electronic primary care data occurred in partnership with EMIS, which provides the software systems to most of the practices in the Bristol area. Records were extracted from all EMIS practices in the Bristol, North Somerset and South Gloucestershire clinical commissioning group which had consented to the NHS South West Commissioning Support Unit (SWCSU) governance framework and data extraction mechanism. Approval for this ALSPAC project was obtained from the SWCSU Security and Informatics Group and the SWCSU informed all participating practices and offered the option to opt-out. For more details, see [3].

Data processing methods after this extraction were identical for both the pilot and main extraction phases. The extracted electronic primary care records were pseudonymised and then securely transferred to a SAIL secure setting (a UK Secure eResearch Platform; UKSeRP) using SAIL's 'split file' method. NHS standards of encryption and security were maintained throughout. These protocols have been described in more detail in previous publications [2,3].

References cited in supplementary information:

1 Ford D V., Jones KH, Verplancke JP, *et al.* The SAIL Databank: Building a national architecture for e-health research and evaluation. *BMC Health Serv Res* 2009;**9**:1–12. doi:10.1186/1472-6963-9-157

2 Cornish R, John A, Boyd A, *et al.* Defining adolescent common mental disorders using electronic primary care data: A comparison with outcomes measured using the CIS-R. *BMJ Open* 2016;**6**. doi:10.1136/bmjopen-2016-013167

3 Cornish RP, Macleod J, Boyd A, *et al.* Factors associated with participation over time in the Avon Longitudinal Study of Parents and Children: a study using linked education and primary care data. *Int J Epidemiol* 2020;:1–10. doi:10.1093/ije/dyaa192

For peer review only

Table S1: Read code lists

Condition	Reference for Read code list (doi) / list of codes
**Anxiety – diagnosis, symptoms, treatment**	Cornish *et al*., 2016: http://dx.doi.org/10.1136/bmjopen-2016-013167
**Depression – diagnosis, symptoms, treatment**	Cornish *et al*., 2016: http://dx.doi.org/10.1136/bmjopen-2016-013167
**ADHD**	E2E.. (Childhood hyperkinetic syndrome)<br>E2E0. (Child attention deficit disorder)<br>E2E00 (Attention deficit without hyperactivity)<br>E2E01 (Attention deficit without hyperactivity)<br>E2E0z (Child attention deficit disorder NOS)<br>E2E1. (Hyperkinesis with developmental delay)<br>E2E2. (Hyperkinetic conduct disorder)<br>E2Ey. (Other hyperkinetic manifestation)<br>E2Ez. (Hyperkinetic syndrome NOS)<br>Eu90. ([X]Hyperkinetic disorders)<br>Eu900 ([X]Attention deficit hyperactivity disorder)<br>Eu901 ([X]Hyperkinetic conduct disorder)<br>Eu902 ([X]Deficits in attention motor control and perception)<br>Eu90y ([X]Other hyperkinetic disorders)<br>Eu90z ([X]Hyperkinetic disorder unspecified)<br>Eu9y7 ([X]Attention deficit disorder)<br>9Ngp. (On drug therapy ADHD)<br>8BPT. (Drug therapy for ADHD)
**Asthma**	Cornish *et al*., 2014: http://dx.doi.org/10.1136/bmjopen-2014-005345
**Autism Spectrum Disorder (ASD)**	Hagberg & Jick, 2017: https://doi.org/10.2147/CLEP.S139107
**Bipolar disorder: diagnosis**	Carr *et al*., 2017: https://dx.doi.org/10.1370%2Fafm.2054 (used diagnosis codes only from their list)
**Conduct disorder**	E2C4. (Mixed disturbance of conduct and emotion)<br>E2C4z (Mixed disturbance of conduct and emotion NOS)<br>Eu912 ([X]Socialized conduct disorder)<br>Eu911 ([X]Conduct disorder, solitary aggressive type)<br>E2C2z (Socialised conduct disorder NOS)<br>E2C1. (Nonaggressive unsocial conduct disorder)<br>Eu911 ([X]Unsocialized conduct disorder)<br>E2C.. (Disturbance of conduct NEC)<br>Eu91. ([X]Conduct disorders)<br>Eu92z ([X]Mixed disorder of conduct and emotions, unspecified)<br>Eu92. ([X]Mixed disorders of conduct and emotions)<br>E2C2. (Socialised conduct disorder)<br>E2C0. (Aggressive unsocial conduct disorder)<br>E2C1z (Nonaggressive unsocial conduct disorder NOS)<br>E2C0z (Aggressive unsocial conduct disorder NOS)<br>Eu901 ([X]Hyperkinetic disorder associated with conduct disorder)<br>E2Czz (Disturbance of conduct NOS)<br>Eu91z ([X]Childhood conduct disorder NOS)<br>Eu92y ([X]Conduct disorder associated with emotional disorder)<br>Eu91y ([X]Other conduct disorders)<br>E2Cy. (Other conduct disturbances)<br>E2Cz. (Unspecified disturbance of conduct)

	E2Cyz (Other conduct disturbances NOS)<br>Eu910 ([X]Conduct disorder confined to the family context)<br>Eu91z ([X]Conduct disorder, unspecified)<br>Eu920 ([X]Depressive conduct disorder)<br>Eu92y ([X]Other mixed disorders of conduct and emotions)<br>Eu913 ([X]Oppositional defiant disorder)<br>E2Dy0 (Childhood and adolescent oppositional disorder)
**Eating disorder**	Carr *et al.*, 2017: https://dx.doi.org/10.1370%2Fafm.2054
**Eczema**	Mukherjee *et al.*, 2014: https://dx.doi.org/10.1136/bmjopen-2014-006647 (used diagnosis codes only from their list)
**Family history depression**	Read codes 1285.
**Family history mental illness**	128% except 1281. (Read codes starting 128 - % used as sort of wild card for Read codes. 1281. is family history senile dementia)
**General symptoms**	1954. (Indigestion)<br>1968. (Abdominal discomfort)<br>1969. (Abdominal pain)<br>1981. (c/o Nausea)<br>1982. (Nausea present)<br>198Z. (Nausea NOS)<br>1B14. (Tenseness)<br>1B18. (Inadequate)<br>1B1G. (Headache)<br>1B1G1 (c/o Headache)<br>1B1N. (Poor self-esteem)<br>1B1O. (Restless)<br>1B51 (Dizziness symptoms)<br>E2781 (Tension headache)<br>R0040 ([D] Dizziness)<br>R00Z2 ([D] Pain, generalised)<br>R040. ([D] Headache)<br>R0700 ([D] Nausea)<br>R090. ([D] Abdominal pain)<br>R090z ([D] Abdominal pain NOS)<br>R090E ([D] Recurrent abdominal pain)<br>R090N ([D] Non-specific abdominal pain)
**History of depression**	1465. (H/O: depression)
**History of mental illness**	146%. (H/O: psychiatric disorder)
**Number of consultations and prescriptions**	Cornish *et al.*, 2020: https://doi.org/10.1093/ije/dyaa192
**Psychosis**	Carr *et al.*, 2017: https://dx.doi.org/10.1370%2Fafm.2054 (used diagnosis codes only from their list)
**Referral to mental health services**	8H49. (Psychiatric referral)<br>8H4P. (Referral to child psychiatrist)<br>8H78. (Referral to counsellor)<br>8H7A. (Referral to mental health worker)<br>8H7B. (Referral to community psychiatric nurse)<br>8H7T. (Referral to psychotherapist)<br>8H7W. (Referral to TOP counselling)<br>8Hc.. (Referral to mental health team)<br>8Hc0. (Referral to community mental health team)<br>8Hc1. (Referral to mental health crisis team)

For peer review only

	8Hc2. (Referral to primary care mental health team)<br>8HHn. (Referral to non-NHS mental health community service)<br>8HHp. (Referral for guided self-help for anxiety)<br>8HHq. (Referral for guided self-help for depression)<br>8HHr. (Referral for mental health self-help literature)<br>8HHu. (Referral to primary care mental health gateway worker)<br>8HHR. (Referral to child and adolescent psychiatry service)<br>8HHT. (Refer to psychotherapist)<br>8HkK. (Referral to improving access to psychological therapies programme)<br>8HVO. (Private referral to psychiatrist)
**Schizophrenia**	Carr *et al*., 2017: https://dx.doi.org/10.1370%2Fafm.2054 (used diagnosis codes only from their list)
**Self-harm**	Thomas *et al*., 2012 : https://dx.doi.org/10.1111%2Fbcp.12059
**Smoking**	Cornish *et al*., 2020: https://doi.org/10.1093/ije/dyaa192
**Somatic symptoms**	16122 (Loss of appetite – symptom)<br>1613. (Increased appetite)<br>1615. (Reduced appetite)<br>168% (Tired all the time, fatigue, tiredness, tearful, malaise/lethargy)<br>1B15. (Irritable)<br>1B16. (Agitated)<br>1B19. (Suicidal)<br>1B1B. (Cannot sleep – insomnia)<br>1B1D. (Nightmares)<br>1B1I. (Crying, excessive)<br>1B1I1 (C/O tearfulness)<br>1BD1. (Suicidal ideation)<br>1BD2. (Morbid thoughts)<br>1BD3. (Suicidal plans)<br>1BD4. (Suicide risk)<br>1BD5. (High suicide risk)<br>1BD6. (Moderate suicide risk)<br>1BD8. (At risk of deliberate self harm)<br>1BDA. (Thoughts of deliberate self harm)<br>1BJ.. (Loss of confidence)<br>1BR.. (Reduced concentration)<br>1BW.. (Poor concentration)<br>E2052 (Tired all the time)<br>E2741 (Insomnia NOS)
**Substance abuse (alc/drugs)**	Eu1% ([X] Mental and behavioural disorders due to psychoactive substance abuse)<br>E23% (Alcohol dependence)<br>E24% (Drug dependence)

Table S2: Details of variables used in the misclassification analyses.

Variable	Origin	Type	Notes
**Sex**	ALSPAC	Binary	0 = Male; 1 = Female
**Age at data collection event**	ALSPAC	Cont.	Measured in months
**Mother's age at birth**	ALSPAC	Cont.	Measured in years
**Mother's home ownership status**	ALSPAC	Binary	0 = Owned/Mortgaged; 1 = Rented/Housing Association/Council House/Other
**Mother's marital status**	ALSPAC	Binary	0 = Married; 1 = Not married
**Mother's age at first pregnancy**	ALSPAC	Cont.	Measured in years
**Mother's parity at child's birth**	ALSPAC	Binary	0 = First child; 1 = Second or later child
**Mother's highest education level**	ALSPAC	Binary	0 = CSE/Vocational/O Level; 1 = A Level/Degree
**Father's highest education level**	ALSPAC	Binary	0 = CSE/Vocational/O Level; 1 = A Level/Degree<br>Assumes Mother's partner is child's Father.
**Mother's social class**	ALSPAC	Binary	0 = I/II – Professional/Managerial; 1 – III/IV/V – Skilled/Partly skilled/Unskilled
**Father's social class**	ALSPAC	Binary	0 = I/II – Professional/Managerial; 1 – III/IV/V – Skilled/Partly skilled/Unskilled<br>Assumes Mother's partner is child's Father.
**Child's ethnic background**	ALSPAC	Binary	1 = White; 2 = Non-white
**Child's education (# A*-C GCSE equivalents)**	ALSPAC	Cont.	From education linkage data
**CIS-R depression score**	ALSPAC	Cat.	Specific to TF4 analyses
**CIS-R diagnosis of moderate depressive episode**	ALSPAC	Binary	Specific to TF4 analysis
**CIS-R diagnosis of severe depressive episode**	ALSPAC	Binary	Specific to TF4 analysis
**CIS-R Generalised Anxiety Disorder (GAD) symptoms**	ALSPAC	Binary	Specific to TF4 analysis
**CIS-R total score**	ALSPAC	Cont.	Specific to TF4 analysis
**DAWBA predicted depression**	ALSPAC	Cat.	Specific to TF3 analyses
**DAWBA predicted anxiety**	ALSPAC	Cat.	Specific to TF3 analyses
**Total MFQ score**	ALSPAC	Cont.	Specific to CCS, CCT, YPA and YPB analyses
**Historical depression diagnosis**	Primary care	Binary	0 = No; 1 = Yes
**Historical anxiety diagnosis**	Primary care	Binary	0 = No; 1 = Yes
**Current anxiety diagnosis**	Primary care	Binary	0 = No; 1 = Yes
**Historical phobia diagnosis**	Primary care	Binary	0 = No; 1 = Yes
**Current phobia diagnosis**	Primary care	Binary	0 = No; 1 = Yes
**Historical depression symptoms**	Primary care	Binary	0 = No; 1 = Yes
**Current depression symptoms**	Primary care	Binary	0 = No; 1 = Yes
**Historical anxiety symptoms**	Primary care	Binary	0 = No; 1 = Yes
**Current anxiety symptoms**	Primary care	Binary	0 = No; 1 = Yes

**Historical somatic symptoms**	Primary care	Binary	0 = No; 1 = Yes
**Current somatic symptoms**	Primary care	Binary	0 = No; 1 = Yes
**Historical general symptoms**	Primary care	Binary	0 = No; 1 = Yes
**Current general symptoms**	Primary care	Binary	0 = No; 1 = Yes
**Historical anti-depressant treatment**	Primary care	Binary	0 = No; 1 = Yes
**Current anti-depressant treatment**	Primary care	Binary	0 = No; 1 = Yes
**Historical anti-anxiety treatment**	Primary care	Binary	0 = No; 1 = Yes
**Current anti-anxiety treatment**	Primary care	Binary	0 = No; 1 = Yes
**# of GP consultations per year**	Primary care	Cont.	Average over 2-year period at time of data collection
**# of GP prescriptions per year**	Primary care	Cont.	Average over 2-year period at time of data collection
**Asthma diagnosis**	Primary care	Binary	0 = No; 1 = Yes (Includes any previous read codes, up to 6 months after age at event)
**Eczema diagnosis**	Primary care	Binary	0 = No; 1 = Yes (Includes any previous read codes, up to 6 months after age at event)
**Eating disorder**	Primary care	Binary	0 = No; 1 = Yes (Includes any previous read codes, up to 6 months after age at event)
**ADHD**	Primary care	Binary	0 = No; 1 = Yes (Includes any previous read codes, up to 6 months after age at event)
**Conduct disorder**	Primary care	Binary	0 = No; 1 = Yes (Includes any previous read codes, up to 6 months after age at event)
**Autism spectrum disorder**	Primary care	Binary	0 = No; 1 = Yes (Includes any previous read codes, up to 6 months after age at event)
**Substance abuse (alcohol/drugs)**	Primary care	Binary	0 = No; 1 = Yes (Includes any previous read codes, up to 6 months after age at event)
**Family history of mental illness**	Primary care	Binary	0 = No; 1 = Yes (Includes any previous read codes, up to 6 months after age at event)
**Family history of depression**	Primary care	Binary	0 = No; 1 = Yes (Includes any previous read codes, up to 6 months after age at event)
**History of mental health issues**	Primary care	Binary	0 = No; 1 = Yes (Includes any previous read codes, up to 6 months after age at event)
**History of depression**	Primary care	Binary	0 = No; 1 = Yes (Includes any previous read codes, up to 6 months after age at event)
**Self-harm**	Primary care	Binary	0 = No; 1 = Yes (Includes any previous read codes, up to 6 months after age at event)
**Other psychological illness (schizophrenia, bipolar disorder and psychosis)**	Primary care	Binary	0 = No; 1 = Yes (Includes any previous read codes, up to 6 months after age at event)
**Any other mental health issues**	Primary care	Binary	0 = No; 1 = Yes (Combining eating disorder, ADHD, conduct disorder, ASD, substance abuse and other psychological illness, as low cell counts for these individual variables)
**Referral to mental health services**	Primary care	Binary	0 = No; 1 = Yes (Includes any previous read codes, up to 6 months after age at event)
**Smoking (or ex-smoker)**	Primary care	Binary	0 = No; 1 = Yes (Includes any previous read codes, up to 6 months after age at event)

Table S3: Details of variables used in the lasso analyses.

Variable	Type	Notes
**Sex**	Binary	0 = Male; 1 = Female
**Age at ALSPAC data collection**	Cont.	Measured in months
**Historical depression diagnosis**	Binary	0 = No; 1 = Yes
**Historical anxiety diagnosis**	Binary	0 = No; 1 = Yes
**Current anxiety diagnosis**	Binary	0 = No; 1 = Yes
**Historical phobia diagnosis**	Binary	0 = No; 1 = Yes
**Current phobia diagnosis**	Binary	0 = No; 1 = Yes
**Historical depression symptoms**	Binary	0 = No; 1 = Yes
**Current depression symptoms**	Binary	0 = No; 1 = Yes
**Historical anxiety symptoms**	Binary	0 = No; 1 = Yes
**Current anxiety symptoms**	Binary	0 = No; 1 = Yes
**Historical somatic symptoms**	Binary	0 = No; 1 = Yes
**Current somatic symptoms**	Binary	0 = No; 1 = Yes
**Historical general symptoms**	Binary	0 = No; 1 = Yes
**Current general symptoms**	Binary	0 = No; 1 = Yes
**Historical anti-depressant treatment**	Binary	0 = No; 1 = Yes
**Current anti-depressant treatment**	Binary	0 = No; 1 = Yes
**Historical anti-anxiety treatment**	Binary	0 = No; 1 = Yes
**Current anti-anxiety treatment**	Binary	0 = No; 1 = Yes
**# of GP consultations per year**	Cont.	Average over 2-year period at time of data collection
**# of GP prescriptions per year**	Cont.	Average over 2-year period at time of data collection
**Asthma diagnosis**	Binary	0 = No; 1 = Yes (Includes any previous read codes, up to 6 months after age at event)
**Eczema diagnosis**	Binary	0 = No; 1 = Yes (Includes any previous read codes, up to 6 months after age at event)
**Eating disorder**	Binary	0 = No; 1 = Yes (Includes any previous read codes, up to 6 months after age at event)
**ADHD**	Binary	0 = No; 1 = Yes (Includes any previous read codes, up to 6 months after age at event)
**Conduct disorder**	Binary	0 = No; 1 = Yes (Includes any previous read codes, up to 6 months after age at event)
**Autism spectrum disorder**	Binary	0 = No; 1 = Yes (Includes any previous read codes, up to 6 months after age at event)
**Substance abuse (alcohol/drugs)**	Binary	0 = No; 1 = Yes (Includes any previous read codes, up to 6 months after age at event)
**Family history of mental illness**	Binary	0 = No; 1 = Yes (Includes any previous read codes, up to 6 months after age at event)
**Family history of depression**	Binary	0 = No; 1 = Yes (Includes any previous read codes, up to 6 months after age at event)

**History of mental health issues**	Binary	0 = No; 1 = Yes (Includes any previous read codes, up to 6 months after age at event)
**History of depression**	Binary	0 = No; 1 = Yes (Includes any previous read codes, up to 6 months after age at event)
**Self-harm**	Binary	0 = No; 1 = Yes (Includes any previous read codes, up to 6 months after age at event)
**Other psychological illness (schizophrenia, bipolar disorder and psychosis)**	Binary	0 = No; 1 = Yes (Includes any previous read codes, up to 6 months after age at event)
**Any other mental health issues**	Binary	0 = No; 1 = Yes (Combining eating disorder, ADHD, conduct disorder, ASD, substance abuse and other psychological illness, as low cell counts for these individual variables)
**Referral to mental health services**	Binary	0 = No; 1 = Yes (Includes any previous read codes, up to 6 months after age at event)
**Smoking (or ex-smoker)**	Binary	0 = No; 1 = Yes (Includes any previous read codes, up to 6 months after age at event)

For peer review only

Table S4: Reasons for having ALSPAC clinic/questionnaire common mental disorder (CMD) data, but not primary care linkage data. Note also that the numbers excluded do not always correspond to the numbers with ALSPAC, but not primary care, data; this is because some individuals may have been counted both in the 'lost from records' and 'first entered records' columns (i.e., they first have primary care data within 18 months of the time-point, then were lost from primary care records again within 6 months after the time-point), or have missing data. Numbers in brackets denote the percentage of individuals in each exclusion category of the total participants without primary care data but with ALSPAC data. Most individuals are likely to be lost from primary care records because they moved out of the Bristol area, but it is also possible that they stayed within the Bristol area but moved to a GP practice using a different software system from which it was not possible to extract primary care data. Similarly, individuals may first appear in the primary care data after the time-point because they either moved into the Bristol area at this time, or stayed in the Bristol area but moved to a GP practice from which it was possible to extract primary care data.

**Age (time point)**	**# with ALSPAC CMD data**	**# with ALSPAC, but not primary care, CMD data**	**# Excluded as no primary care data (no consent or no records)**	**# Excluded as lost from primary care records before time-point**	**# Excluded as first primary care data is after time-point**
**Age 15/16 (TF3 clinic)**	5,332	1,669	737 (44.2%)	614 (36.8%)	313 (18.8%)
**Age 16/17 (CCS quest)**	4,950	1,737	762 (43.9%)	690 (39.7%)	283 (16.3%)
**Age 17/18 (TF4 clinic)**	4,534	1,450	583 (40.2%)	616 (42.5%)	254 (17.5%)
**Age 18/19 (CCT quest)**	3,302	1,320	510 (38.6%)	601 (45.5%)	214 (16.2%)
**Age 21/22 (YPA quest)**	3,283	1,985	471 (23.7%)	1,462 (73.7%)	64 (3.2%)
**Age 22/23 (YPB quest)**	3,896	2,571	563 (21.9%)	1,969 (76.6%)	43 (1.7%)

For peer review only

Table S5: Demographics at age 16/17 CCS questionnaire, 17/18 TF4 clinic, 18/19 CCT questionnaire and 21/22 YPA questionnaire time points. Of those with ALSPAC data, the table compares those who have primary data against those who do not. For categorical variables cells are counts and percentages. For continuous variables cells are means and standard deviations. Note also that the denominators vary as the variables come from different data sources, with different levels of completeness. See table 3 for age 15/16 TF3 and 22/23 YPB questionnaire time points.

	**Age 16/17 (CCS questionnaire) – SMFQ (Depression)**		**Age 17/18 (TF4 clinic) – CIS-R (CMDs and Depression)**		**Age 18/19 (CCT questionnaire) – SMFQ (Depression)**		**Age 21/22 (YPA questionnaire) – SMFQ (Depression)**	
	**Primary care data (*n*=3213)**	**No primary care data (*n*=1737)**	**Primary care data (*n*=3084)**	**No primary care data (*n*=1450)**	**Primary care data (*n*=1982)**	**No primary care data (*n*=1320)**	**Primary care data (*n*=1228)**	**No primary care data (*n*=1985)**
**Sex**								
**Male**	1302 (40.5%)	706 (40.6%)	1358 (44.0%)	624 (43.0%)	703 (35.5%)	470 (35.6%)	456 (35.1%)	710 (35.8%)
**Female**	1911 (59.5%)	1031 (59.4%)	1726 (56.0%)	826 (57.0%)	1279 (64.5%)	850 (64.4%)	842 (64.9%)	1275 (64.2%)
**Maternal age at child's birth**	29.4 (4.6)	29.4 (4.5)	29.3 (4.6)	29.2 (4.6)	29.4 (4.6)	29.8 (4.6)	29.2 (4.6)	29.8 (4.4)
**Mother's home ownership status**								
**Owned/ Mortgaged**	2549 (85.8%)	1359 (83.6%)	2405 (85.4%)	1115 (83.7%)	1567 (85.2%)	1052 (85.1%)	1015 (85%)	1632 (87.6%)
**Rented**	138 (4.6%)	104 (6.4%)	113 (4%)	69 (5.2%)	83 (4.5%)	70 (5.7%)	53 (4.4%)	94 (5%)
**Council/Housing Association**	220 (7.4%)	101 (6.2%)	229 (8.1%)	101 (7.6%)	148 (8.1%)	72 (5.8%)	98 (8.2%)	89 (4.8%)
**Other**	65 (2.2%)	62 (3.8%)	69 (2.5%)	48 (3.6%)	41 (2.2%)	42 (3.4%)	28 (2.4%)	49 (2.6%)
**Mother's marital status**								
**Never married**	380 (12.7%)	214 (13%)	386 (13.6%)	190 (14.1%)	242 (13%)	130 (10.4%)	146 (12.1%)	206 (11%)
**Single/Divorced**	132 (4.4%)	86 (5.2%)	122 (4.3%)	70 (5.2%)	78 (4.2%)	56 (4.5%)	48 (4%)	80 (4.3%)
**First marriage**	2305 (76.9%)	1225 (74.6%)	2165 (76.3%)	998 (74%)	1435 (77%)	979 (78.2%)	924 (76.6%)	1470 (78.4%)
**2nd/3rd marriage**	182 (6.1%)	118 (7.2%)	164 (5.8%)	91 (6.8%)	108 (5.8%)	87 (7%)	88 (7.3%)	120 (6.4%)
**Mother's parity**								
**0**	1410 (47.3%)	840 (51.5%)	1326 (47.2%)	670 (50.6%)	876 (47.4%)	629 (50.8%)	556 (46.5%)	933 (50.2%)
**1**	1058 (35.5%)	553 (33.9%)	999 (35.6%)	444 (33.6%)	666 (36%)	413 (33.4%)	433 (36.2%)	631 (34%)
**2 or more**	511 (17.5%)	238 (14.6%)	482 (17.2%)	209 (15.8%)	307 (16.6%)	196 (15.8%)	207 (17.3%)	294 (15.8%)
**Mother's highest education level**								
**O level/lower**	1612 (54.1%)	777 (47.7%)	1541 (55.2%)	642 (48.2%)	993 (53.9%)	542 (43.6%)	726 (60.8%)	826 (44.3%)
**A level**	825 (27.7%)	468 (28.7%)	756 (27.1%)	400 (30%)	496 (26.9%)	367 (29.6%)	300 (25.1%)	555 (29.7%)
**Degree**	544 (18.3%)	385 (23.6%)	495 (17.7%)	291 (21.8%)	355 (19.3%)	333 (26.8%)	168 (14.1%)	485

								(26%)
**Father's highest education level**								
**O level/lower**	1217 (44.4%)	556 (36.6%)	1143 (44.5%)	502 (40.7%)	737 (43.7%)	391 (33.2%)	538 (49.3%)	604 (34.2%)
**A level**	828 (30.2%)	465 (30.6%)	795 (30.9%)	363 (29.4%)	510 (30.3%)	365 (31%)	344 (31.5%)	528 (29.9%)
**Degree**	699 (25.5%)	498 (32.8%)	632 (24.6%)	368 (29.9%)	439 (26%)	423 (35.9%)	209 (19.2%)	634 (35.9%)
**Child ethnicity**								
**White**	2836 (96.4%)	1535 (95.6%)	2639 (95.8%)	1255 (95.4%)	1752 (96%)	1186 (96.3%)	1138 (96.2%)	1769 (95.9%)
**Non-white**	105 (3.6%)	70 (4.4%)	116 (4.2%)	60 (4.6%)	74 (4%)	46 (3.7%)	45 (3.8%)	75 (4.1%)
**# GCSEs (or equivalents)**	7.7 (3.4)	7.8 (3.4)	7.5 (3.5)	7.6 (3.4)	7.8 (3.3)	8.4 (3.1)	7.5 (3.5)	8.5 (3)
**ALSPAC depression diagnosis**								
**No**	2728 (84.9%)	1452 (83.6%)	2841 (92.1%)	1334 (92%)	1595 (80.5%)	1095 (83%)	1098 (84.6%)	1685 (84.9%)
**Yes**	485 (15.1%)	285 (16.4%)	243 (7.9%)	116 (8%)	387 (19.5%)	225 (17.1%)	200 (15.4%)	300 (15.1%)
**ALSPAC common mental disorder (CMD) diagnosis**								
**No**	-	-	2629 (85.3%)	1231 (84.9%)	-	-	-	-
**Yes**	-	-	455 (14.8%)	219 (15.1%)	-	-	-	-

SMFQ: Short Mood and Feelings Questionnaire; *CMDs:* Common mental disorders; *CIS-R:* Clinical Interview Schedule – Revised.

Table S6: Comparing ALSPAC participants who possess primary care data at each time-point, split by whether they attended/completed each specific data collection event. The numbers with and without ALSPAC data are provided, as are differences in rates of current depression or common mental disorder (CMD) diagnoses from the primary care records. For participants who did not attend the clinic/complete the questionnaire, the age to define a 'current' diagnosis was based on +/- 6 months from the average age each clinic/questionnaire was completed. Individuals who have GP data and completed the clinic/questionnaire, but do not have ALSPAC-derived depression/CMD data (as this session was not completed for whatever reason), are not included in the table below. The number of these individuals at each time point are: 104 at the age 15/16 TF3 clinic (2.8% of those with both ALSPAC and primary care data); 82 at the age 16/17 CCS questionnaire (2.5% of those with both ALSPAC and primary care data); 437 at the age 17/18 TF4 clinic (12.4% of those with both ALSPAC and primary care data); 20 at the age 18/19 CCT questionnaire (1% of those with both ALSPAC and primary care data); 63 at the age 21/22 YPA questionnaire (4.6% of those with both ALSPAC and primary care data); 43 at the age 22/23 YPB questionnaire (3.1% of those with both ALSPAC and primary care data). For a graphical summary of these results, see figure 1.

**Age (time point)**	**Ave. age at clinic/quest (years (months))**	**ALSPAC measure completed**	**# with and without ALSPAC CMD/ depression data (%)**	**# with current depression diagnosis in GP records (%)**	**# with current CMD diagnosis in GP records (%)**
**Age 15/16 (TF3 clinic)**	15.5 years (186 mths)	Yes	3,663 (42.8%)	7 (0.2%)	26 (0.7%)
		No	4,902 (57.2%)	8 (0.2%)	29 (0.6%)
**Age 16/17 (CCS quest)**	16.67 years (200 mths)	Yes	3,213 (37.6%)	17 (0.5%)	37 (1.2%)
		No	5,342 (62.4%)	25 (0.5%)	43 (0.8%)
**Age 17/18 (TF4 clinic)**	17.83 years (214 mths)	Yes	3,084 (38.3%)	30 (1%)	57 (1.9%)
		No	4,961 (61.7%)	45 (0.9%)	76 (1.5%)
**Age 18/19 (CCT quest)**	18.67 years (224 mths)	Yes	1,982 (23.6%)	24 (1.2%)	53 (2.7%)
		No	6,402 (76.4%)	78 (1.2%)	131 (2.1%)
**Age 21/22 (YPA quest)**	21.92 years (263 mths)	Yes	1,298 (20.4%)	35 (2.7%)	62 (4.8%)
		No	5,055 (79.6%)	106 (2.1%)	155 (3.1%)
**Age 22/23 (YPB quest)**	22/92 years (275 mths)	Yes	1,325 (23.4%)	30 (2.3%)	70 (5.3%)
		no	4,335 (76.6%)	81 (1.9%)	128 (3%)

For peer review only

Table S7: Raw data comparing depression and common mental disorder (CMD) diagnoses based on the Development and Well-Being Assessment (DAWBA) data from the age 15/16 TF3 clinic against various definitions derived from the primary care data at this age (*n*=3,663). Note that values with an asterisk have been suppressed for disclosure control purposes as at least one cell has a value < 5. This table also includes sensitivities, specificities, positive predictive values (PPV) and negative predictive values (NPV) for the depression and CMD diagnoses based on the DAWBA data from this clinic. In these analyses we are treating the ALSPAC data as the reference standard.

		DAWBA Depression			DAWBA CMD		
**Primary care definition**		**No**	**Yes**	**Total**	**No**	**Yes**	**Total**
**Current diagnosis**	No	*	*	*	3,521	116	3,637
	Yes	*	*	*	19	7	26
**Sensitivity**		*			5.7% (2.3; 11.4)		
**Specificity**		*			99.5% (99.2; 99.7)		
**Positive Predictive Value**		*			26.9% (11.6; 47.8)		
**Negative Predictive Value**		*			96.8% (96.2; 97.4)		
		**No**	**Yes**	**Total**	**No**	**Yes**	**Total**
**Current diagnosis, treated**	No	*	*	*	*	*	*
	Yes	*	*	*	*	*	*
**Sensitivity**		*			*		
**Specificity**		*			*		
**Positive Predictive Value**		*			*		
**Negative Predictive Value**		*			*		
		**No**	**Yes**	**Total**	**No**	**Yes**	**Total**
**Current diagnosis or symptoms or treatment**	No	3,576	52	3,628	3,491	109	3,600
	Yes	30	5	35	49	14	63
**Sensitivity**		8.8% (2.9; 19.3)			11.4% (6.4; 18.4)		
**Specificity**		99.2% (98.8; 99.4)			98.6% (98.2; 99)		
**Positive Predictive Value**		14.3% (4.8; 30.3)			22.2% (12.7; 34.5)		
**Negative Predictive Value**		98.6% (98.1; 98.9)			97 (96.4; 97.5)		

For peer review only

Table S8: Raw data comparing depression diagnoses based on the Short Mood and Feelings Questionnaire (SMFQ) data from the age 16/17 CCS questionnaire against various definitions derived from the primary care data at this age (*n*=3,213). Note that values with an asterisk have been suppressed for disclosure control purposes as at least one cell has a value < 5. This table also includes sensitivities, specificities, positive predictive values (PPV) and negative predictive values (NPV) for the depression diagnoses based on the SMFQ data from this questionnaire. In these analyses we are treating the ALSPAC data as the reference standard.

		**SMFQ Depression**		
**Primary care definition**		**No**	**Yes**	**Total**
**Current diagnosis**	No	2,719	477	3,196
	Yes	9	8	17
**Sensitivity**		1.6% (0.7; 3.2)		
**Specificity**		99.7% (99.4; 99.8)		
**Positive Predictive Value**		47.1% (23; 72.2)		
**Negative Predictive Value**		85.1% (83.8; 86.3)		
		**No**	**Yes**	**Total**
**Current diagnosis, treated**	No	*	*	*
	Yes	*	*	*
**Sensitivity**		*		
**Specificity**		*		
**Positive Predictive Value**		*		
**Negative Predictive Value**		*		
		**No**	**Yes**	**Total**
**Current diagnosis or symptoms or treatment**	No	2,694	451	3,145
	Yes	34	34	68
**Sensitivity**		7% (4.9; 9.7)		
**Specificity**		98.8% (98.3; 99.1)		
**Positive Predictive Value**		50% (37.6; 62.4)		
**Negative Predictive Value**		85.7% (84.4; 86.9)		

For peer review only

For peer review only - http://bmjopen.bmj.com/site/about/guidelines.xhtml

Table S9: Raw data comparing depression diagnoses based on the Short Mood and Feelings Questionnaire (SMFQ) data from the age 18/19 CCT questionnaire against various definitions derived from the primary care data at this age (*n*=1,982). This table also includes sensitivities, specificities, positive predictive values (PPV) and negative predictive values (NPV) for the depression diagnoses based on the SMFQ data from this questionnaire. In these analyses we are treating the ALSPAC data as the reference standard.

		**SMFQ Depression**		
**Primary care definition**		**No**	**Yes**	**Total**
**Current diagnosis**	No	1,586	372	1,958
	Yes	9	15	24
**Sensitivity**		3.9% (2.2; 6.3)		
**Specificity**		99.4% (98.9; 99.7)		
**Positive Predictive Value**		62.5% (40.6; 81.2)		
**Negative Predictive Value**		81% (79.2; 82.7)		
		**No**	**Yes**	**Total**
**Current diagnosis, treated**	No	1,590	374	1,964
	Yes	5	13	18
**Sensitivity**		3.4% (1.8; 5.7)		
**Specificity**		99.7% (99.3; 99.9)		
**Positive Predictive Value**		72.2% (46.5; 90.3)		
**Negative Predictive Value**		81% (79.1; 82.7)		
		**No**	**Yes**	**Total**
**Current diagnosis or symptoms or treatment**	No	1,542	320	1,862
	Yes	53	67	120
**Sensitivity**		17.3% (13.7; 21.5)		
**Specificity**		96.7% (95.7; 97.5)		
**Positive Predictive Value**		55.8% (46.5; 64.9)		
**Negative Predictive Value**		82.8% (81; 84.5)		

For peer review only

Table S10: Raw data comparing depression diagnoses based on the Short Mood and Feelings Questionnaire (SMFQ) data from the age 21/22 YPA questionnaire against various definitions derived from the primary care data at this age (*n*=1,298). This table also includes sensitivities, specificities, positive predictive values (PPV) and negative predictive values (NPV) for the depression diagnoses based on the SMFQ data from this questionnaire. In these analyses we are treating the ALSPAC data as the reference standard.

		SMFQ Depression		
**Primary care definition**		**No**	**Yes**	**Total**
**Current diagnosis**	No	1,082	181	1,263
	Yes	16	19	35
**Sensitivity**		9.5% (5.8; 14.4)		
**Specificity**		98.5% (97.6; 99.2)		
**Positive Predictive Value**		54.3% (36.6; 71.2)		
**Negative Predictive Value**		85.7% (83.6; 87.6)		
		**No**	**Yes**	**Total**
**Current diagnosis, treated**	No	1,086	182	1,268
	Yes	12	18	30
**Sensitivity**		9% (5.4; 13.9)		
**Specificity**		98.9% (98.1; 99.4)		
**Positive Predictive Value**		60% (40.6; 77.3)		
**Negative Predictive Value**		85.6% (83.6; 87.5)		
		**No**	**Yes**	**Total**
**Current diagnosis or symptoms or treatment**	No	1,026	132	1,158
	Yes	72	68	140
**Sensitivity**		34% (27.5; 41)		
**Specificity**		93.4% (91.8; 94.8)		
**Positive Predictive Value**		48.6% (40; 57.2)		
**Negative Predictive Value**		88.6% (86.6; 90.4)		

For peer review only

Table S11: Raw data comparing depression diagnoses based on the Short Mood and Feelings Questionnaire (SMFQ) data from the age 22/23 YPB questionnaire against various definitions derived from the primary care data at this age (*n*=1,326). This table also includes sensitivities, specificities, positive predictive values (PPV) and negative predictive values (NPV) for the depression diagnoses based on the SMFQ data from this questionnaire. In these analyses we are treating the ALSPAC data as the reference standard.

		**SMFQ Depression**		
**Primary care definition**		**No**	**Yes**	**Total**
**Current diagnosis**	No	1,093	202	1,295
	Yes	10	20	30
**Sensitivity**		9% (5.6; 13.6)		
**Specificity**		99.1% (98.3; 99.6)		
**Positive Predictive Value**		66.7% (47.2; 82.7)		
**Negative Predictive Value**		84.4% (82.3; 86.3)		
		**No**	**Yes**	**Total**
**Current diagnosis, treated**	No	1,095	205	1,300
	Yes	8	17	25
**Sensitivity**		7.7% (4.5; 12)		
**Specificity**		99.3% (98.6; 99.7)		
**Positive Predictive Value**		68% (46.5; 85.1)		
**Negative Predictive Value**		84.2% (82.1; 86.2)		
		**No**	**Yes**	**Total**
**Current diagnosis or symptoms or treatment**	No	1,035	161	1,196
	Yes	68	61	129
**Sensitivity**		27.5% (21.7; 33.9)		
**Specificity**		93.8% (92.2; 95.2)		
**Positive Predictive Value**		47.3% (38.4; 56.3)		
**Negative Predictive Value**		86.5% (84.5; 88.4)		

For peer review only

Table S12: Results of the identification in primary care records analysis, based on whether individuals who were diagnosed have having depression or common mental disorders (CMDs) in ALSPAC were also diagnosed based on primary care record data (with primary care diagnosis defined as 'current diagnosis or treatment or symptoms'). Odds ratios are displayed (with 95% confidence intervals). Due to the small sample sizes in some analyses these estimates are rather imprecise, especially regarding the age 15/16 TF3 clinic as very few individuals were classified correctly/diagnosed as depressed/CMD in primary care records. Coefficients are odds ratios derived from univariable logistic regressions and denote the odds of identification relative to the baseline (e.g., for age 17/18 TF4 clinic depression, females have three times greater odds of being identified than males). Note also that when comparing against ALSPAC data (e.g., mother's marital status, parental education, etc.) the sample size for each analysis will vary as the variables come from different data sources, with different levels of completeness. For a graphical illustration of key results, see figure S1.

Variable	*Age 15/16 TF3 clinic*		*Age 16/17 CCS questionnaire*	*Age 17/18 TF4 clinic*		*Age 18/19 CCT questionnaire*	*Age 21/22 YPA questionnaire*	*Age 22/23 YPB questionnaire*
	Dep. (n = 57)	*CMD (n = 123)*	*Dep. (n = 485)*	*Dep. (n = 243)*	*CMD (n = 455)*	*Dep. (n = 387)*	*Dep. (n = 200)*	*Dep. (n = 222)*
Sex (ref = Male)	0.55 (0.08; 3.66)	0.71 (0.2; 2.45)	1.38 (0.56; 3.43)	3.30 (1.33; 8.14)	3.14 (1.61; 6.13)	0.83 (0.44; 1.55)	1.54 (0.78; 3.04)	1.34 (0.68; 2.63)
Age at data collection event (months)	0.81 (0.55; 1.19)	0.89 (0.73; 1.08)	0.93 (0.81; 1.08)	0.98 (0.92; 1.06)	0.98 (0.92; 1.03)	0.98 (0.94; 1.03)	1.02 (0.97; 1.07)	0.98 (0.94; 1.03)
Mother's age at birth (years)	0.89 (0.72; 1.11)	0.91 (0.8; 1.04)	0.96 (0.89; 1.04)	0.95 (0.89; 1.01)	0.96 (0.91; 1.01)	1.01 (0.96; 1.07)	0.9 (0.84; 0.97)	1 (0.93; 1.06)
Mother's home ownership status (ref = owned/mort.)	0.63 (0.06; 6.09)	2.23 (0.65; 7.68)	0.91 (0.34; 2.44)	1.65 (0.81; 3.35)	1.31 (0.73; 2.34)	1.41 (0.71; 2.8)	0.92 (0.43; 1.99)	0.8 (0.32; 1.98)
Mother's marital status (ref = married)	1.38 (0.21; 9.07)	2.61 (0.8; 8.48)	0.77 (0.29; 2.06)	1.48 (0.76; 2.87)	0.98 (0.57; 1.72)	1.25 (0.67; 2.36)	1.43 (0.68; 3.01)	1.02 (0.45; 2.28)
Mother's age at first pregnancy (years)	1.16 (0.88; 1.53)	0.89 (0.77; 1.03)	1 (0.93; 1.08)	0.93 (0.87; 0.99)	0.96 (0.91; 1)	1 (0.95; 1.05)	0.97 (0.91; 1.02)	0.99 (0.93; 1.05)
Mother's parity at child's birth (ref = first child)	0.38 (0.04; 3.91)	1.49 (0.41; 5.4)	0.68 (0.32; 1.43)	0.92 (0.5; 1.7)	1.2 (0.73; 1.96)	1.26 (0.72; 2.21)	0.84 (0.45; 1.56)	1.04 (0.56; 1.92)
Mother's highest education level (ref = CSE/Voc./O level)	NC	0.35 (0.07; 1.66)	0.83 (0.38; 1.81)	0.78 (0.41; 1.47)	1.09 (0.66; 1.8)	0.9 (0.51; 1.57)	1.1 (0.58; 2.11)	1.26 (0.66; 2.4)
Father's highest education level (ref = CSE/Voc./O level)	4.06 (0.39; 42.49)	2.32 (0.63; 8.51)	0.77 (0.35; 1.7)	1.16 (0.61; 2.21)	1.18 (0.71; 1.98)	1.2 (0.68; 2.14)	0.85 (0.45; 1.61)	0.67 (0.36; 1.26)
Mother's social class (ref = I/II)	NC	NC	0.96 (0.44; 2.1)	1.33 (0.67; 2.65)	0.86 (0.5; 1.47)	0.9 (0.5; 1.64)	0.77 (0.39; 1.54)	0.43 (0.22; 0.85)
Father's social class (ref = I/II)	0.56 (0.08; 3.66)	1.04 (0.33; 3.35)	1.74 (0.76; 3.99)	1.57 (0.81; 3.05)	1 (0.59; 1.68)	1.24 (0.7; 2.21)	1.31 (0.69; 2.48)	0.77 (0.4; 1.47)
Child's ethnic background (ref = white)	NC	0.91 (0.1; 7.9)	0.87 (0.11; 6.8)	0.37 (0.04; 2.99)	0.67 (0.19; 2.32)	0.75 (0.16; 3.42)	1.23 (0.2; 7.59)	1.22 (0.3; 5.06)
Child's education (# A-C GCSE equivalents)*	0.93 (0.72; 1.22)	0.92 (0.79; 1.06)	0.89 (0.8; 0.98)	0.97 (0.9; 1.05)	0.99 (0.92; 1.05)	0.94 (0.88; 1.01)	1.02 (0.94; 1.1)	1 (0.92; 1.08)
CIS-R depression score (ref = 1 for depression model; ref = 0 for CMD model)	NA	NA	NA	2: 1.44 (0.54; 3.83)<br>3: 2.21 (0.88; 5.54)<br>4: 3.78 (1.5; 9.53)	1: 1.97 (0.83; 4.7)<br>2: 2.85 (1.21; 6.67)<br>3: 5.73 (2.57; 12.8)	NA	NA	NA

					4: 10.08 (4.4; 23.09)			
CIS-R diagnosis of moderate depressive episode (ref = no)	NA	NA	NA	2.49 (1.26; 4.92)	3.23 (2; 5.22)	NA	NA	NA
CIS-R diagnosis of severe depressive episode (ref = no)	NA	NA	NA	2.96 (1.39; 6.29)	5.01 (2.44; 10.3)	NA	NA	NA
CIS-R GAD symptoms (ref = no)	NA	NA	NA	2.11 (1.15; 3.88)	1.49 (0.93; 2.38)	NA	NA	NA
CIS-R total score	NA	NA	NA	1.09 (1.04; 1.15)	1.11 (1.07; 1.15)	NA	NA	NA
DAWBA predicted depression (ref = ~50%)	>70%: 1.38 (0.14; 13.95)	~0.5%: 0.29 (0.03; 2.58)<br>~15%: 2.32 (0.67; 8.05)<br>>70%: 1.08 (0.11; 10.47)	NA	NA	NA	NA	NA	NA
DAWBA predicted anxiety (ref = ~50%)	~3%: 2.53 (0.25; 25.39)	~3%: 1.45 (0.41; 5.15)	NA	NA	NA	NA	NA	NA
Total SMFQ score	NA	NA	1.11 (1.02; 1.21)	NA	NA	1.09 (1.03; 1.16)	1.14 (1.06; 1.22)	1.08 (1.01; 1.16)
Historical depression diagnosis	NC	NC	14.03 (1.91; 102.9)	8.12 (2.03; 32.52)	14.56 (4.57; 46.38)	13.86 (4.2; 45.71)	3.27 (1.52; 7.03)	3.07 (1.46; 6.46)
Historical anxiety diagnosis	34 (2.36; 489.96)	8.92 (1.15; 69.18)	7.22 (2.55; 20.43)	2.34 (0.71; 7.68)	2.24 (0.97; 5.18)	6.09 (2.47; 15.01)	2.81 (1.31; 6.01)	1.95 (0.87; 4.33)
Current anxiety diagnosis	NC	NA	13.59 (4.28; 43.16)	16.94 (1.94; 148.16)	NA	9.76 (3.68; 25.87)	10.16 (2.79; 37.09)	5.07 (1.75; 14.63)
Historical depression symptoms	NC	NC	4.69 (1.6; 13.7)	5.5 (2.21; 13.65)	5.34 (2.58; 11.08)	4.71 (2.29; 9.68)	5.6 (2.86; 10.97)	4.75 (2.52; 8.97)
Historical somatic symptoms	4.08 (0.34; 48.86)	8.32 (1.92; 36.05)	3.77 (1.51; 9.4)	1.72 (0.82; 3.61)	1.66 (0.92; 2.98)	2.53 (1.38; 4.66)	3.77 (2.01; 7.07)	2.59 (1.37; 4.9)
Current somatic symptoms	NC	1.32 (0.15; 11.85)	2.47 (0.69; 8.89)	3.5 (1.32; 9.29)	6.18 (2.78; 13.76)	3.73 (1.65; 8.46)	1.78 (0.62; 5.13)	12.9 (3.5; 47.57)
Historical general symptoms	0.68 (0.07; 6.6)	1.78 (0.51; 6.26)	2.63 (1.27; 5.45)	3.35 (1.79; 6.27)	3.46 (2.07; 5.75)	4.04 (2.32; 7.06)	3.62 (1.91; 6.88)	1.79 (0.97; 3.31)
Current general symptoms	3 (0.27; 33.64)	1.12 (0.13; 9.85)	2.2 (0.62; 7.84)	3.99 (1.29; 12.4)	2.38 (1.02; 5.54)	2.15 (0.9; 5.14)	3.67 (1.04; 13.02)	3.35 (1.08; 10.4)
Historical anti-depressant treatment	NC	NC	11.89 (3.04; 46.61)	3.97 (1.53; 10.31)	7.46 (3.22; 17.26)	23.37 (9.47; 57.66)	9.87 (5; 19.44)	9.59 (4.83; 19.04)
Historical anti-anxiety treatment	NC	8.31 (0.49; 140.9)	3.46 (0.71; 26.98)	1.8 (0.58; 5.6)	3.74 (1.5; 9.34)	6.24 (2.78; 14.01)	4.03 (1.9; 8.55)	5.49 (2.68; 11.27)
Current anti-anxiety treatment	NC	NA	6.98 (1.23; 39.59)	6.8 (2.39; 19.32)	NA	12.9 (5.02; 33.15)	10.16 (2.79; 37.08)	5.4 (1.73; 16.84)
# of GP consultations per year	1.07 (0.86; 1.34)	1.06 (0.93; 1.2)	1.17 (1.11; 1.23)	1.25 (1.16; 1.34)	1.24 (1.17; 1.31)	1.13 (1.07; 1.18)	1.25 (1.15; 1.35)	1.23 (1.15; 1.32)
# of GP prescriptions per year	0.93 (0.64; 1.37)	0.88 (0.69; 1.11)	1.22 (1.11; 1.35)	1.25 (1.14; 1.38)	1.29 (1.19; 1.39)	1.25 (1.14; 1.36)	1.44 (1.26; 1.64)	1.37 (1.22; 1.54)
Asthma diagnosis	1.64 (0.25; 10.85)	1.28 (0.37; 4.41)	1.77 (0.83; 3.76)	0.74 (0.37; 1.48)	0.77 (0.44; 1.36)	1.44 (0.79; 2.61)	1 (0.51; 1.94)	1.82 (0.95; 3.51)
Eczema diagnosis	1.07 (0.16; 6.95)	0.57 (0.17; 1.93)	1.6 (0.79; 3.27)	1.35 (0.71; 2.55)	1.13 (0.69; 1.85)	1.36 (0.79; 2.34)	1.18 (0.65; 2.14)	0.72 (0.39; 1.33)
Eating disorder	NC	5.89 (0.89; 38.83)	5.58 (1.04; 29.87)	3.31 (0.92; 11.88)	5.28 (1.73; 16.13)	2.27 (0.76; 6.75)	1.12 (0.32; 3.95)	1.96 (0.6; 6.44)

For peer review only

History of mental health issues	NC	2.72 (0.26; 28.08)	1.49 (0.18; 12.11)	12.25 (2.47; 60.76)	7.96 (2.28; 27.85)	8.54 (2.7; 27.02)	8.67 (1.79; 42.05)	6.83 (1.71; 27.34)
Self-harm	5.11 (0.7; 37.06)	3.97 (0.9; 17.61)	4.15 (1.44; 11.97)	3.72 (1.61; 8.57)	4.46 (2.21; 9.01)	3.61 (1.7; 7.69)	3.23 (1.3; 8)	4.31 (1.56; 11.92)
Any other mental health issues	NC	9.64 (1.73; 53.63)	2.47 (0.69; 8.89)	1.78 (0.63; 5.04)	2.86 (1.25; 6.54)	2.08 (0.83; 5.23)	1.12 (0.45; 2.83)	1.58 (0.63; 3.99)
Referral to mental health services	1.92 (0.18; 20.11)	7.01 (1.89; 25.97)	3.39 (1.37; 8.4)	5.84 (2.83; 12.07)	5.76 (3.19; 10.42)	6.85 (3.45; 13.61)	3.71 (1.79; 7.65)	4.22 (2.08; 8.56)
Smoking (or ex-smoker)	NC	2.1 (0.4; 11.08)	2.08 (0.9; 4.82)	2.92 (1.54; 5.52)	3.38 (2.01; 5.71)	2.26 (1.26; 4.05)	1.24 (0.67; 2.28)	1.15 (0.61; 2.16)

NA = Not applicable (either because measure not available at that time-point, or because the variable is part of the GP diagnosis variable; e.g., current anti-anxiety treatment is part of the 'current diagnosis or symptoms or treatment' definition of CMDs)

NC = Not calculable (due to collinearity in model as a result of lack of data). Variables where half or more of all analyses were not calculable due to collinearity have been removed from this table, as sample sizes are likely to be too low to be informative. These variables are: historical phobia diagnosis, current phobia diagnosis, historical anxiety symptoms, current anxiety symptoms, ADHD, conduct disorder, autism spectrum disorder, substance abuse, family history of mental illness, family history of depression, personal history of depression and other psychological illness.

For peer review only

Figure S1 (following page): Graphical summary of key results of the identification in primary care records analysis, based on whether individuals who were diagnosed have having depression or common mental disorders (CMDs) in ALSPAC were also diagnosed based on primary care record data (with primary care diagnosis defined as 'current diagnosis or treatment or symptoms'). Values for depression are displayed in black, and common mental disorders are in red. Odds ratios and 95% confidence intervals are displayed on the y-axis (on the log scale), with time point along the x-axis (going forwards in time, from the age 15/16 TF3 clinic to the age 22/23 YPB questionnaire. Due to the small sample sizes in some analyses these estimates are rather imprecise, especially regarding the 15/16 clinic as very few individuals were classified correctly/diagnosed as depressed/CMD in primary care records. Coefficients are odds ratios derived from univariable logistic regressions and denote the odds of identification relative to the baseline (e.g., for age 17/18 TF4 clinic depression, females have three times greater odds of being identified than males). Note also that when comparing against ALSPAC data (e.g., mother's marital status, parental education, etc.) the sample size for each analysis will vary as the variables come from different data sources, with different levels of completeness. For a full list of results, see table S11.

For peer review only

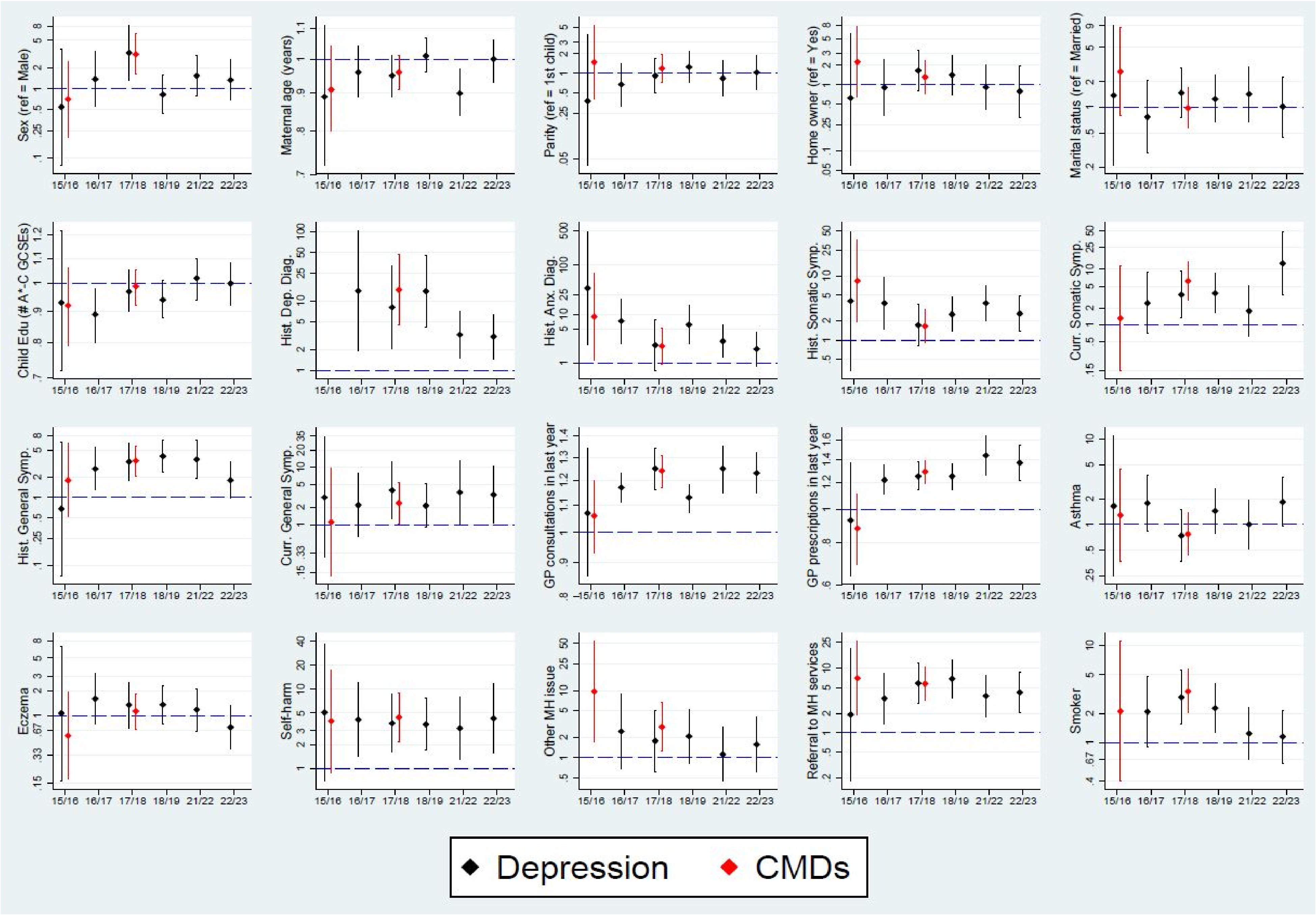
Sex (ref = Male)
Maternal age (years)
Parity (ref = 1st child)
Home owner (ref = Yes)
Marital status (ref = Married)
Child Edu (# A*-C GCSEs)
Hist. Dep. Diag.
Hist. Anx. Diag.
Hist. Somatic Symp.
Curr. Somatic Symp.
Hist. General Symp.
Curr. General Symp.
GP consultations in last year
GP prescriptions in last year
Asthma
Eczema
Self-harm
Other MH issue
Referral to MH services
Smoker
15/16
16/17
17/18
18/19
21/22
22/23
Depression
CMDs

Table S13: Penalised coefficients from the optimal lasso prediction models, predicting ALSPAC questionnaire-derived depression or common mental disorder (CMD) cases using only linked primary care record data. Coefficients are log-odds estimates. Note that as the estimates are derived from lasso models used for model prediction, standard errors are not calculated.

	**Age 15/16 TF3 clinic**		**Age 16/17 CCS quest**	**Age 17/18 TF4 clinic**		**Age 18/19 CCT quest**	**Age 21/22 YPA quest**	**Age 22/23 YPB quest**
	Dep.	CMD	Dep.	Dep.	CMD	Dep.	Dep.	Dep.
**Constant**	-4.307	-3.788	-2.602	-3.24	-5.966	-1.864	-2.036	-5.805
**Self-harm status (ref: no)**	2.297	1.225	0.476	1.469	1.301	1.536	-	0.538
**Historical general symptoms (ref: no)**	0.464	0.143	0.204	-	-	-	-	-
**Mean number of GP consultations**	0.02	0.036	0.03	0.046	0.019	0.004	-	-
**Historical depression diagnosis (ref: no)**	0.84	-	-	-	-	-	0.421	0.071
**Current depression diagnosis (ref: no)**	0.478	1.445	0.217	0.472	0.68	0.69	-	0.606
**Sex (ref: male)**	-	0.544	0.777	0.437	0.493	0.449	-	-
**Eating disorder (ref: no)**	-	0.748	-	0.791	-	-	-	-
**Referral to mental health services (ref: no)**	-	0.242	1.042	-	0.525	0.22	0.539	0.29
**Current phobia diagnosis (ref: no)**	-	1.434	-	-	-	2.281	-	-
**Current anxiety diagnosis (ref: no)**	-	0.349	1.035	-	0.016	1.057	-	-
**Current antidepressant use (ref: no)**	-	0.312	-	0.808	0.618	0.725	0.73	0.82
**Current depression symptoms (ref: no)**	-	-	0.218	1.156	0.76	-	0.033	0.502
**Smoking status (ref: no)**	-	-	0.466	0.058	-	-	-	-
**Historical depression symptoms (ref: no)**	-	-	0.469	-	0.388	0.457	0.18	0.54
**Historical somatic symptoms (ref: no)**	-	-	0.125	-	0.185	0.016	-	-
**Autism spectrum disorder (ref: no)**	-	-	2.131	-	-	-	-	0.702
**Family history of depression (ref: no)**	-	-	2.61	-	-	-	-	-
**Conduct disorder (ref: no)**	-	-	0.89	-	-	-	-	-
**ADHD (ref: no)**	-	-	-0.622	-	-	-	-	-
**Substance abuse (ref: no)**	-	-	0.881	-	-	-	-	-
**Historical phobia diagnosis (ref: no)**	-	-	0.373	-	-	-	-	0.447
**Historical anxiety symptoms (ref: no)**	-	-	0.292	-	-	-	-	-0.634
**Historical anti-anxiety meds (ref: no)**	-	-	-	0.63	0.17	0.426	-	-
**Current anti-anxiety meds (ref: no)**	-	-	-	0.251	-	-	0.122	-
**Eczema (ref: no)**	-	-	-	0.027	-	-	-	-

**Historical antidepressant use (ref: no)**	-	-	-	0.058	0.135	-0.066	0.613	0.688
**Historical anxiety diagnosis (ref: no)**	-	-	-	-	0.524	-	-	-
**Age (months)**	-	-	-	-	0.017	-	-	0.014
**Current general symptoms (ref: no)**	-	-	-	-	0.189	-	-	-
**Current somatic symptoms (ref: no)**	-	-	-	-	-	0.613	-	-
**History of depression (ref: no)**	-	-	-	-	-	0.808	-	-
**History of mental health issues (ref: no)**	-	-	-	-	-	-0.198	-	-
**Other psychological illness (ref: no)**	-	-	-	-	-	-0.013	-	-

For peer review only

Table S14: Full models to estimate the predicted probability of ALSPAC questionnaire-derived depression or common mental disorders (CMDs) at each time-point, based on the best-fitting lasso model using only linked primary care record data as predictors. Code is provided in Stata syntax.

**Time-point (*n*)**	**Diagnosis**	**Model to estimate predicted probability of diagnosis**
**Age 15/16 TF3 clinic (*n* = 3,663)**	Depression	gen TF3_dep_DAWBA = (exp(-4.306898 + (selfharm_TF3 * 2.297141) + (hist_gensym_TF3 * 0.4635765) + (consult_mean_TF3 * 0.0201705) + (hist_depress_TF3 * 0.8404444) + (curr_depress_TF3 * 0.4780882))) / (1 (exp(-4.306898 + (selfharm_TF3 * 2.297141) + (hist_gensym_TF3 * 0.4635765) + (consult_mean_TF3 * 0.0201705) + (hist_depress_TF3 * 0.8404444) + (curr_depress_TF3 * 0.4780882))))
	CMD	gen TF3_CMD_DAWBA = (exp(-3.78843 + (sex * 0.5437601) + (selfharm_TF3 * 1.224612) + (consult_mean_TF3 * 0.0356085) + (curr_depress_TF3 * 1.444507) + (eatdisorder_TF3 * 0.7475241) + (hist_gensym_TF3 * 0.1431262) + (referral_TF3 * 0.2418507) + (curr_phob_TF3 * 1.434107) + (curr_anx_TF3 * 0.3485559) + (curr_depdrugs_TF3 * 0.3121066))) / (1 + (exp(-3.78843 + (sex * 0.5437601) + (selfharm_TF3 * 1.224612) + (consult_mean_TF3 * 0.0356085) + (curr_depress_TF3 * 1.444507) + (eatdisorder_TF3 * 0.7475241) + (hist_gensym_TF3 * 0.1431262) + (referral_TF3 * 0.2418507) + (curr_phob_TF3 * 1.434107) + (curr_anx_TF3 * 0.3485559) + (curr_depdrugs_TF3 * 0.3121066))))
**Age 16/17 CCS quest (*n* = 3,213)**	Depression	gen CCS_dep_SMFQ = (exp(-2.602106 + (sex * 0.7768598) + (referral_CCS * 1.042151) + (asd_CCS * 2.131247) + (smoke_CCS * 0.4664435) + (consult_mean_CCS * 0.0301841) + (curr_anx_CCS * 1.03486) + (fhdep_CCS * 2.610094) + (hist_gensym_CCS * 0.203526) + (selfharm_CCS * 0.4762654) + (conduct_CCS * 0.8898849) + (hist_depsymp_CCS * 0.4685372) + (adhd_CCS * -0.6223789) + (hist_somsym_CCS * 0.1245751) + (alcdrug_CCS * 0.8809454) + (hist_phob_CCS * 0.3725191) + (curr_depsymp_CCS * 0.2181478) + (curr_depress_CCS * 0.2167674) + (hist_anxsymp_CCS * 0.2916632))) / (1 + (exp(-2.602106 + (sex * 0.7768598) + (referral_CCS * 1.042151) + (asd_CCS * 2.131247) + (smoke_CCS * 0.4664435) + (consult_mean_CCS * 0.0301841) + (curr_anx_CCS * 1.03486) + (fhdep_CCS * 2.610094) + (hist_gensym_CCS * 0.203526) + (selfharm_CCS * 0.4762654) + (conduct_CCS * 0.8898849) + (hist_depsymp_CCS * 0.4685372) + (adhd_CCS * -0.6223789) + (hist_somsym_CCS * 0.1245751) + (alcdrug_CCS * 0.8809454) + (hist_phob_CCS * 0.3725191) + (curr_depsymp_CCS * 0.2181478) + (curr_depress_CCS * 0.2167674) + (hist_anxsymp_CCS * 0.2916632))))
**Age 17/18 TF4 clinic (*n* = 3,084)**	Depression	gen TF4_dep_CISR = (exp(-3.239794 + (sex * 0.436772) + (selfharm_TF4 * 1.469416) + (consult_mean_TF4 * 0.0459718) + (referral_TF4 * 0.7906288) + (curr_depsymp_TF4 * 1.155598) + (curr_depdrugs_TF4 * 0.8078427) + (hist_anxdrugs_TF4 * 0.6298965) + (curr_depress_TF4 * 0.4717727) + (curr_anxdrugs_TF4 * 0.2511227) + (smoke_TF4 * 0.0582065) + (eczema * 0.0274914) + (hist_depdrugs_TF4 * 0.0581372))) / (1 + (exp(-3.239794 + (sex * 0.436772) + (selfharm_TF4 * 1.469416) + (consult_mean_TF4 * 0.0459718) + (referral_TF4 * 0.7906288) + (curr_depsymp_TF4 * 1.155598) + (curr_depdrugs_TF4 * 0.8078427) + (hist_anxdrugs_TF4 * 0.6298965) + (curr_depress_TF4 * 0.4717727) + (curr_anxdrugs_TF4 * 0.2511227) + (smoke_TF4 * 0.0582065) + (eczema * 0.0274914) + (hist_depdrugs_TF4 * 0.0581372))))
	CMD	gen TF4_CMD_CISR = (exp(-5.96604 + (sex * 0.4931608) + (selfharm_TF4 * 1.301296) + (referral_TF4 * 0.5245662) + (curr_depsymp_TF4 * 0.7602904) + (curr_depdrugs_TF4 * 0.6179576) + (consult_mean_TF4 * 0.0194573) + (hist_anx_TF4 *

		0.5237928) + (TF4_agemths * 0.0168863) + + (curr_depress_TF4 * 0.6795285) + (hist_depsymp_TF4 * 0.3876382) + (hist_somsym_TF4 * 0.184908) + (curr_gensym_TF4 * 0.189229) + (hist_anxdrugs_TF4 * 0.1699545) + (hist_depdrugs_TF4 * 0.1345062) + (curr_anx_TF4 * 0.0156222))) / (1 + (exp(-5.96604 + (sex * 0.4931608) + (selfharm_TF4 * 1.301296) + (referral_TF4 * 0.5245662) + (curr_depsymp_TF4 * 0.7602904) + (curr_depdrugs_TF4 * 0.6179576) + (consult_mean_TF4 * 0.0194573) + (hist_anx_TF4 * 0.5237928) + (TF4_agemths * 0.0168863) + + (curr_depress_TF4 * 0.6795285) + (hist_depsymp_TF4 * 0.3876382) + (hist_somsym_TF4 * 0.184908) + (curr_gensym_TF4 * 0.189229) + (hist_anxdrugs_TF4 * 0.1699545) + (hist_depdrugs_TF4 * 0.1345062) + (curr_anx_TF4 * 0.0156222))))
**Age 18/19 CCT quest (*n* = 1,982)**	Depression	gen CCS_dep_SMFQ = (exp(-1.864214 + (selfharm_CCT * 1.536287) + (sex * 0.4488152) + (curr_depdrugs_CCT * 0.7249568) + (curr_anx_CCT * 1.056577) + (curr_somsym_CCT * 0.6129295) + (curr_phob_CCT * 2.280971) + (hist_depsymp_CCT * 0.4572833) + (hist_anxdrugs_CCT * 0.4263218) + (curr_depress_CCT * 0.6901121) + (referral_CCT * 0.220459) + (histdep_CCT * 0.807517) + (histmh_CCT * -0.197546) + (consult_mean_CCT * 0.0.0041373) + (hist_depdrugs_CCT * -0.0660863) + (hist_somsym_CCT * 0.015973) + (CCT_agemths * -0.0003042) + (psych_other_CCT * -0.0130987))) / (1 + (exp(-1.864214 + (selfharm_CCT * 1.536287) + (sex * 0.4488152) + (curr_depdrugs_CCT * 0.7249568) + (curr_anx_CCT * 1.056577) + (curr_somsym_CCT * 0.6129295) + (curr_phob_CCT * 2.280971) + (hist_depsymp_CCT * 0.4572833) + (hist_anxdrugs_CCT * 0.4263218) + (curr_depress_CCT * 0.6901121) + (referral_CCT * 0.220459) + (histdep_CCT * 0.807517) + (histmh_CCT * -0.197546) + (consult_mean_CCT * 0.0.0041373) + (hist_depdrugs_CCT * -0.0660863) + (hist_somsym_CCT * 0.015973) + (CCT_agemths * -0.0003042) + (psych_other_CCT * -0.0130987))))
**Age 21/22 YPA quest (*n* = 1,298)**	Depression	gen YPA_dep_SMFQ = (exp(-2.036359 + (curr_depdrugs_YPA * 0.7299155) + (hist_depdrugs_YPA * 0.6132707) + (referral_YPA * 0.5390764) + (hist_depress_YPA * 0.4207832) + (hist_depsymp_YPA * 0.1804129) + (curr_anxdrugs_YPA * 0.122329) + (curr_depsymp_YPA * 0.033265))) / (1 + (exp(-2.036359 + (curr_depdrugs_YPA * 0.7299155) + (hist_depdrugs_YPA * 0.6132707) + (referral_YPA * 0.5390764) + (hist_depress_YPA * 0.4207832) + (hist_depsymp_YPA * 0.1804129) + (curr_anxdrugs_YPA * 0.122329) + (curr_depsymp_YPA * 0.033265))))
**Age 22/23 YPB quest (*n* = 1,325)**	Depression	gen YPB_dep_SMFQ = (exp(-5.804938 + (hist_depdrugs_YPB * 0.6876923) + (curr_depdrugs_YPB * 0.8200612) + (hist_depsymp_YPB * 0.5401723) + (curr_depress_YPB * 0.6056239) + (hist_anxsymp_YPB * -0.6341232) + (selfharm_YPB * 0.5377375) + (YPB_agemths * 0.0136081) + (curr_depsymp_YPB * 0.502017) + (referral_YPB * 0.2895768) + (asd_YPB * 0.7015883) + (hist_phob_YPB * 0.4473284) + (hist_depress_YPB * 0.0714968))) / (1 + (exp(-5.804938 + (hist_depdrugs_YPB * 0.6876923) + (curr_depdrugs_YPB * 0.8200612) + (hist_depsymp_YPB * 0.5401723) + (curr_depress_YPB * 0.6056239) + (hist_anxsymp_YPB * -0.6341232) + (selfharm_YPB * 0.5377375) + (YPB_agemths * 0.0136081) + (curr_depsymp_YPB * 0.502017) + (referral_YPB * 0.2895768) + (asd_YPB * 0.7015883) + (hist_phob_YPB * 0.4473284) + (hist_depress_YPB * 0.0714968))))

Table S15: In-sample and out-of-sample deviance ratios for common mental disorders (CMDs) or depression at each time-point. Deviance ratios are taken from logistic cross-validation lasso models. The full models are based on the set of all the primary care variables described in table S3. The 'diagnosis only' models contain just the relevant 'current' diagnosis variables (for depression models, this is just current depression diagnosis; for CMD models, this is current depression, anxiety and phobia diagnosis). The 'diagnosis, symptoms and treatment' models contain the relevant 'current' diagnosis, symptoms or treatment variables (for depression models, this is current depression diagnosis, current depressive symptoms and current antidepressant use; for CMD models, this is current depression, anxiety and phobia diagnosis, current depressive and anxiety symptoms, and current antidepressant and anti-anxiety medications).

Time-point (*n*)	Diagnosis	Lasso model	Sample	Deviance ratio
**Age 15/16 TF3 clinic (*n* = 3,663)**	Depression	Full	Training	9.9%
			Validation	-1.3%
		Diagnosis only	Training	0%
			Validation	-0.9%
		Diagnosis, symptoms and treatment	Training	0%
			Validation	-0.9%
	CMD	Full	Training	8.4%
			Validation	2.9%
		Diagnosis only	Training	2%
			Validation	-1.2%
		Diagnosis, symptoms and treatment	Training	1.6%
			Validation	-1.5%
**Age 16/17 CCS questionnaire (*n* = 3,213)**	Depression	Full	Training	8.3%
			Validation	4.3%
		Diagnosis only	Training	0.4%
			Validation	0.3%
		Diagnosis, symptoms and treatment	Training	0.8%
			Validation	1.8%
**Age 17/18 TF4 clinic (*n* = 3,084)**	Depression	Full	Training	14.6%
			Validation	7.8%
		Diagnosis only	Training	2.4%
			Validation	1.1%
		Diagnosis, symptoms and treatment	Training	7.6%
			Validation	6.9%
	CMD	Full	Training	9.2%
			Validation	7.8%
		Diagnosis only	Training	3.8%
			Validation	4.9%
		Diagnosis, symptoms and treatment	Training	3.8%
			Validation	5.2%
**Age 18/19 CCT questionnaire (*n* = 1,982)**	Depression	Full	Training	9%
			Validation	8%
		Diagnosis only	Training	1.3%
			Validation	0.8%

For peer review only - http://bmjopen.bmj.com/site/about/guidelines.xhtml

		Diagnosis, symptoms and treatment	Training	3.2%
			Validation	5.3%
**Age 21/22 YPA questionnaire (*n* = 1,298)**	Depression	Full	Training	11.7%
			Validation	12.6%
		Diagnosis only	Training	3.3%
			Validation	1.6%
		Diagnosis, symptoms and treatment	Training	8.8%
			Validation	10%
**Age 22/23 YPB questionnaire (*n* = 1,325)**	Depression	Full	Training	13.4%
			Validation	9.1%
		Diagnosis only	Training	3.5%
			Validation	2.7%
		Diagnosis, symptoms and treatment	Training	7.9%
			Validation	5%

peer review only

Table S16: Sensitivities and specificities from the 40% validation sample, assessing how well the lasso model predicts common mental disorders (CMDs) or depression in ALSPAC across each of the time-points. Sensitivities and specificities are also given for the three 'CMD/depression' definitions using only primary care diagnosis, symptoms and/or treatment data (all using the same 40% validation sample). In these analyses we are treating the ALSPAC data as the reference standard. Note that for disclosure control purposes, statistics calculated where at least one cell in the cross-tabulation has a value <5 have been suppressed and replaced with a '<' or '>' summary statistic.

	**Sensitivity**	**Specificity**
**Age 15/16 TF3 clinic depression – DAWBA (*n*=1,465; *n* with ALSPAC depression = 19)**		
**Lasso prediction model**	0% (0; 17.6)[a]	100% (99.7; 100)[a]
**Current diagnosis**	0% (0; 17.6)[a]	>99%
**Current diagnosis, treated**	0% (0; 17.6)[a]	100% (99.7; 100)[a]
**Current diagnosis or symptoms or treatment**	<10%	>99%
**Age 15/16 TF3 clinic CMD – DAWBA (*n*=1,465; *n* with ALSPAC CMD = 40)**		
**Lasso prediction model**	0% (0; 8.8)[a]	100% (99.7; 100)[a]
**Current diagnosis**	<5%	>99%
**Current diagnosis, treated**	0% (0; 8.8)[a]	100% (99.7; 10)[a]
**Current diagnosis or symptoms or treatment**	<10%	>98%
**Age 16/17 CCS questionnaire depression – SMFQ (*n*=1,285; *n* with ALSPAC depression = 197)**		
**Lasso prediction model**	3.5% (1.4; 7.2)	99% (98.2; 99.5)
**Current diagnosis**	<3%	>99%
**Current diagnosis, treated**	<3%	>99%
**Current diagnosis or symptoms or treatment**	8.1% (4.7; 12.9)	99% (98.2; 99.5)
**Age 17/18 TF4 clinic depression – CIS-R (*n*=1,234; *n* with ALSPAC depression = 100)**		
**Lasso prediction model**	6% (2.2; 12.6)	99.1% (98.4; 99.6)
**Current diagnosis**	5% (1.6; 11.3)	99.3% (98.6; 99.7)
**Current diagnosis, treated**	<5%	>99%
**Current diagnosis or symptoms or treatment**	26% (17.7; 35.7)	97.3% (96.1; 98.1)
**Age 17/18 TF4 clinic CMD – CIS-R (*n*=1,234; *n* with ALSPAC CMD = 187)**		
**Lasso prediction model**	8% (4.6; 12.9)	99.1% (98.4; 99.6)
**Current diagnosis**	7% (3.8; 11.6)	98.5% (97.5; 99.1)
**Current diagnosis, treated**	3.7% (1.5; 7.6)	99.4% (98.8; 99.8)
**Current diagnosis or symptoms or treatment**	23% (17.2; 29.7)	96.4% (95.1; 97.4)
**Age 18/19 CCT questionnaire depression – SMFQ (*n*=793; *n* with ALSPAC depression = 152)**		
**Lasso prediction model**	13.2% (8.2; 19.6)	98.1% (96.8; 99)
**Current diagnosis**	3.9% (1.5; 8.4)	99.2% (98.2; 99.7)
**Current diagnosis, treated**	<5%	>99%
**Current diagnosis or symptoms or treatment**	22.4% (16; 29.8)	97.7% (96.2; 98.7)

For peer review only - http://bmjopen.bmj.com/site/about/guidelines.xhtml

**Age 21/22 YPA questionnaire depression – SMFQ (*n*=519; *n* with ALSPAC depression = 82)**		
**Lasso prediction model**	<10%	>99%
**Current diagnosis**	7.3% (2.7; 15.2)	98.6% (97)
**Current diagnosis, treated**	6.1% (2; 13.7)	98.9% (97.4; 99.6)
**Current diagnosis or symptoms or treatment**	36.6% (26.2; 48)	93.6% (90.9; 95.7)
**Age 22/23 YPB questionnaire depression – SMFQ (*n*=530; *n* with ALSPAC depression = 92)**		
**Lasso prediction model**	16.3% (9.4; 25.5)	98.2% (96.4; 99.2)
**Current diagnosis**	<10%	>99%
**Current diagnosis, treated**	<10%	>99%
**Current diagnosis or symptoms or treatment**	25% (16.6; 35.1)	93.8% (91.2; 95.9)

[a] One-sided 97.5% confidence interval.

DAWBA: Development and Well-Being Assessment; *SMFQ*: Short Mood and Feelings Questionnaire; *CIS-R:* Clinical Interview Schedule – Revised.

For peer review only

Table S17: Examining the association between primary care depression and common mental disorder (CMD) diagnoses and having ALSPAC data at each time point. For each time point, we present three sets of analyses: i) unadjusted models exploring the univariable associations between having ALSPAC data and both depression and CMDs; ii) models adjusting for sex; and iii) models adjusting for both sex and maternal education (a proxy for SEP; operationalised as a binary variable with the categories 'CSE/Vocational/O level' vs 'A level/Degree'). All results are odds ratios with 95% confidence intervals in brackets.

	**Depression**	**CMDs**
**Age 15/16 TF3 clinic**		
Unadjusted model (n = 8,565)	1.17 (0.42; 3.23)	1.2 (0.71; 2.04)
Adjusted for sex (n = 8,565)	1.16 (0.42; 3.21)	1.14 (0.67; 1.93)
Adjusted for sex & maternal education (n = 7,342)	1.38 (0.41; 4.66)	1.05 (0.58; 1.89)
**Age 16/17 CCS questionnaire**		
Unadjusted model (n = 8,555)	1.31 (0.61; 2.1)	1.44 (0.92; 2.23)
Adjusted for sex (n = 8,555)	1 (0.53; 1.86)	1.28 (0.82; 2.01)
Adjusted for sex & maternal education (n = 7,335)	1.09 (0.55; 2.13)	1.18 (0.72; 1.93)
**Age 17/18 TF4 clinic**		
Unadjusted model (n = 8,045)	1.07 (0.67; 1.71)	1.21 (0.86; 1.71)
Adjusted for sex (n = 8,045)	0.93 (0.58; 1.48)	1.07 (0.75; 1.52)
Adjusted for sex & maternal education (n = 6,786)	0.99 (0.59; 1.66)	1.04 (0.71; 1.54)
**Age 18/19 CCT questionnaire**		
Unadjusted model (n = 8,384)	0.99 (0.63; 1.57)	1.32 (0.95; 1.82)
Adjusted for sex (n = 8,384)	0.76 (0.48; 1.21)	1.04 (0.75; 1.44)
Adjusted for sex & maternal education (n = 7,066)	0.92 (0.56; 1.5)	1.12 (0.79; 1.59)
**Age 21/22 YPA questionnaire**		
Unadjusted model (n = 6,353)	1.29 (0.88; 1.91)	1.59 (1.17; 2.14)
Adjusted for sex (n = 6,353)	1.06 (0.72; 1.57)	1.33 (0.98; 1.81)
Adjusted for sex & maternal education (n = 5,300)	1.12 (0.74; 1.7)	1.38 (1; 1.91)
**Age 22/23 YPB questionnaire**		
Unadjusted model (n = 5,660)	1.22 (0.8; 1.86)	1.83 (1.36; 2.47)
Adjusted for sex (n = 5,660)	0.94 (0.61; 1.45)	1.5 (1.1; 2.03)
Adjusted for sex & maternal education (n = 4,668)	1.1 (0.69; 1.75)	1.65 (1.82; 2.3)

STROBE 2007 (v4) Statement—Checklist of items that should be included in reports of *cohort studies*

Section/Topic	Item #	Recommendation	Reported on page #
**Title and abstract**	1	(*a*) Indicate the study's design with a commonly used term in the title or the abstract	1 and 2
		(*b*) Provide in the abstract an informative and balanced summary of what was done and what was found	1 and 2
**Introduction**			
Background/rationale	2	Explain the scientific background and rationale for the investigation being reported	4 and 5
Objectives	3	State specific objectives, including any prespecified hypotheses	5
**Methods**			
Study design	4	Present key elements of study design early in the paper	5 to 8
Setting	5	Describe the setting, locations, and relevant dates, including periods of recruitment, exposure, follow-up, and data collection	5 to 7
Participants	6	(*a*) Give the eligibility criteria, and the sources and methods of selection of participants. Describe methods of follow-up	5 to 8
		(*b*) For matched studies, give matching criteria and number of exposed and unexposed	N/A
Variables	7	Clearly define all outcomes, exposures, predictors, potential confounders, and effect modifiers. Give diagnostic criteria, if applicable	5 to 9 (plus supplementary tables S1 to S3)
Data sources/ measurement	8*	For each variable of interest, give sources of data and details of methods of assessment (measurement). Describe comparability of assessment methods if there is more than one group	5 to 9 (plus supplementary tables S1 to S3)
Bias	9	Describe any efforts to address potential sources of bias	See discussion (pages 15 to 17)
Study size	10	Explain how the study size was arrived at	5 and 6 (and Table 1)
Quantitative variables	11	Explain how quantitative variables were handled in the analyses. If applicable, describe which groupings were chosen and why	5 to 9 (plus supplementary tables S1 to S3)
Statistical methods	12	(*a*) Describe all statistical methods, including those used to control for confounding	8 and 9
		(*b*) Describe any methods used to examine subgroups and interactions	N/A

For peer review only

		(*c*) Explain how missing data were addressed	See discussion (page 14 to 17)
		(*d*) If applicable, explain how loss to follow-up was addressed	See discussion (page 14 to 17)
		(*e*) Describe any sensitivity analyses	N/A
**Results**			
Participants	13*	(a) Report numbers of individuals at each stage of study—eg numbers potentially eligible, examined for eligibility, confirmed eligible, included in the study, completing follow-up, and analysed	5 to 9 (Tables 1, 3, S4 and S5)
		(b) Give reasons for non-participation at each stage	Table S4
		(c) Consider use of a flow diagram	N/A
Descriptive data	14*	(a) Give characteristics of study participants (eg demographic, clinical, social) and information on exposures and potential confounders	9 to 11 (Table 3)
		(b) Indicate number of participants with missing data for each variable of interest	9 (Tables 1 and S4)
		(c) Summarise follow-up time (eg, average and total amount)	N/A
Outcome data	15*	Report numbers of outcome events or summary measures over time	12 (Tables 1, 4, S7 to S11)
Main results	16	(*a*) Give unadjusted estimates and, if applicable, confounder-adjusted estimates and their precision (eg, 95% confidence interval). Make clear which confounders were adjusted for and why they were included	9 to 14 (Table 4, S7 to S13, S16, S17)
		(*b*) Report category boundaries when continuous variables were categorized	6 and 7
		(*c*) If relevant, consider translating estimates of relative risk into absolute risk for a meaningful time period	N/A
Other analyses	17	Report other analyses done—eg analyses of subgroups and interactions, and sensitivity analyses	16 (Table S17)
**Discussion**			
Key results	18	Summarise key results with reference to study objectives	15 to 17
**Limitations**			
Interpretation	20	Give a cautious overall interpretation of results considering objectives, limitations, multiplicity of analyses, results from similar studies, and other relevant evidence	3, 15 to 17
Generalisability	21	Discuss the generalisability (external validity) of the study results	15 and 16
**Other information**			
Funding	22	Give the source of funding and the role of the funders for the present study and, if applicable, for the original study on which the present article is based	18

For peer review only

*Give information separately for cases and controls in case-control studies and, if applicable, for exposed and unexposed groups in cohort and cross-sectional studies.

Note: An Explanation and Elaboration article discusses each checklist item and gives methodological background and published examples of transparent reporting. The STROBE checklist is best used in conjunction with this article (freely available on the Web sites of PLoS Medicine at http://www.plosmedicine.org/, Annals of Internal Medicine at http://www.annals.org/, and Epidemiology at http://www.epidem.com/). Information on the STROBE Initiative is available at www.strobe-statement.org.

For peer review only

BMJ Open

Assessing and predicting adolescent and early adulthood common mental disorders using electronic primary care data: analysis of a prospective cohort study (ALSPAC) in southwest England

Journal:	*BMJ Open*
Manuscript ID	bmjopen-2021-053624.R1
Article Type:	Original research
Date Submitted by the Author:	03-Sep-2021
Complete List of Authors:	Smith, Daniel; University of Bristol, MRC Integrative Epidemiology Unit; University of Bristol, Population Health Sciences, Bristol Medical School<br>Willan, Kathryn ; Bradford Teaching Hospitals NHS Foundation Trust, Born in Bradford<br>Prady, Stephanie; University of York, Department of Health Sciences<br>Dickerson, Josie; Bradford Teaching Hospitals NHS Foundation Trust, Born in Bradford<br>Santorelli, Gillian; Bradford Teaching Hospitals NHS Foundation Trust, Born in Bradford<br>Tilling, Kate; University of Bristol, MRC Integrative Epidemiology Unit; University of Bristol, Population Health Sciences, Bristol Medical School<br>Cornish, Rosie; University of Bristol, MRC Integrative Epidemiology Unit; University of Bristol, Population Health Sciences, Bristol Medical School
**Primary Subject Heading**:	Epidemiology
Secondary Subject Heading:	Mental health
Keywords:	Depression & mood disorders < PSYCHIATRY, PRIMARY CARE, Anxiety disorders < PSYCHIATRY, EPIDEMIOLOGY, Child & adolescent psychiatry < PSYCHIATRY

SCHOLARONE™
Manuscripts

I, the Submitting Author has the right to grant and does grant on behalf of all authors of the Work (as defined in the below author licence), an exclusive licence and/or a non-exclusive licence for contributions from authors who are: i) UK Crown employees; ii) where BMJ has agreed a CC-BY licence shall apply, and/or iii) in accordance with the terms applicable for US Federal Government officers or employees acting as part of their official duties; on a worldwide, perpetual, irrevocable, royalty-free basis to BMJ Publishing Group Ltd ("BMJ") its licensees and where the relevant Journal is co-owned by BMJ to the co-owners of the Journal, to publish the Work in this journal and any other BMJ products and to exploit all rights, as set out in our licence.

The Submitting Author accepts and understands that any supply made under these terms is made by BMJ to the Submitting Author unless you are acting as an employee on behalf of your employer or a postgraduate student of an affiliated institution which is paying any applicable article publishing charge ("APC") for Open Access articles. Where the Submitting Author wishes to make the Work available on an Open Access basis (and intends to pay the relevant APC), the terms of reuse of such Open Access shall be governed by a Creative Commons licence – details of these licences and which Creative Commons licence will apply to this Work are set out in our licence referred to above.

Other than as permitted in any relevant BMJ Author's Self Archiving Policies, I confirm this Work has not been accepted for publication elsewhere, is not being considered for publication elsewhere and does not duplicate material already published. I confirm all authors consent to publication of this Work and authorise the granting of this licence.

For peer review only

Assessing and predicting adolescent and early adulthood common mental disorders using electronic primary care data: analysis of a prospective cohort study (ALSPAC) in southwest England

Daniel Smith[1,2], Kathryn Willan[3], Stephanie L Prady[4], Josie Dickerson[3], Gillian Santorelli[3], Kate Tilling[1,2], Rosie P Cornish[1,2]

[1] MRC Integrative Epidemiology Unit at the University of Bristol, UK
[2] Population Health Sciences, Bristol Medical School, University of Bristol, UK
[3] Born in Bradford, Bradford Teaching Hospitals NHS Foundation Trust, Bradford, UK
[4] Department of Health Sciences, University of York, UK

Corresponding authors: Daniel Smith (dan.smith@bristol.ac.uk) and Rosie P Cornish (Rosie.Cornish@bristol.ac.uk)

ORCIDs:
DS: 0000-0001-6467-2023
KW: 0000-0002-1566-6054
SLP: 0000-0002-8933-8045
JD: 0000-0003-0121-3406
GS: 0000-0003-0427-1783
RC: 0000-0002-2874-7646

Word count: 6,288
Tables: 5
Figures: 2

For peer review only - http://bmjopen.bmj.com/site/about/guidelines.xhtml

For peer review only

Abstract

Objectives: We aimed to examine agreement between common mental disorders (CMDs) from primary care records and repeated CMD questionnaire data from ALSPAC (the Avon Longitudinal Study of Parents and Children) over adolescence and young adulthood, explore factors affecting CMD identification in primary care records, and construct models predicting ALSPAC-derived CMDs using only primary care data.

Design and Setting: Prospective cohort study (ALSPAC) in southwest England with linkage to electronic primary care records.

Participants: Primary care records were extracted for 11,807 participants (80% of 14,731 eligible). Between 31% (3,633; age 15/16) and 11% (1,298; age 21/22) of participants had both primary care and ALSPAC CMD data.

Outcome measures: ALSPAC outcome measures were diagnoses of suspected depression and/or CMDs. Primary care outcome measure were Read codes for diagnosis, symptoms and treatment of depression/CMDs.

For each time point, sensitivities and specificities for primary care CMD diagnoses were calculated for predicting ALSPAC-derived measures of CMDs, and the factors associated with identification of primary care-based CMDs in those with suspected ALSPAC-derived CMDs explored. Lasso models were used at each time point to predict ALSPAC-derived CMDs using only primary care data, with internal validation by randomly splitting data into 60% training and 40% validation samples.

Results: Sensitivities for primary care diagnoses were low for CMDs (range: 3.5 to 19.1%) and depression (range: 1.6 to 34.0%), while specificities were high (nearly all >95%). The strongest predictors of identification in the primary care data for those with ALSPAC-derived CMDs were symptom severity indices. The lasso models had relatively low prediction rates, especially in the validation sample (deviance ratio range: -1.3 to 12.6%), but improved with age.

Conclusions: Primary care data underestimate CMDs compared to population-based studies. Improving GP identification, and using free-text or secondary care data, is needed to improve the accuracy of models using clinical data.

Keywords: ALSPAC, Common Mental Disorders, Depression, Primary Care Data, Data Linkage, Predictive Modelling

For peer review only - http://bmjopen.bmj.com/site/about/guidelines.xhtml

Strengths and limitations of this study

- We used a large prospective cohort (ALSPAC) with validated mental health questionnaires to assess depression and common mental disorders (which we treat as our 'reference standard') and were able to link these data to individuals' electronic primary care records, with this linkage data covering ~80% of the cohort.
- We were able to assess agreement between ALSPAC data and electronic primary care data for common mental disorders across adolescence and into adulthood, a key life transition and period where mental health problems often emerge.
- There is a risk of selection bias, as many participants with primary care data did not have ALSPAC mental health measures, while primary care data coverage also decreased with age; continued participation in both cases is likely to be non-random.
- For this study we assumed that the common mental disorder measures from ALSPAC are the 'reference standard' against which the primary care data should be compared; however, these measures may also be subject to misclassification.
- The available linkage data consisted of primary care Read codes, which misses data from other clinical sources, such as secondary care or from primary care free-text data.

For peer review only - http://bmjopen.bmj.com/site/about/guidelines.xhtml

INTRODUCTION

Common mental disorders (CMDs; depression and anxiety) are a leading cause of morbidity, disability and premature death worldwide [1]. Rates of CMDs have increased over the past few decades [2], including in adolescence and early adulthood [3], where these conditions frequently first appear [4,5]. The prevalence of CMDs in childhood and adolescence (age 5-16) in the UK is estimated to be 4% [6], rising to 16% among 16-24 year-olds [7]; these can have significant long-term consequences, including on education, quality-of-life, employment and physical and mental health [5,8,9].

Assessing the prevalence of CMDs in the population, especially in adolescence, is essential for monitoring, research and planning of appropriate public health services. Estimates of prevalence could be from population studies (which are expensive and time-consuming to conduct), or using primary (General Practitioner; GP) and secondary (hospital and specialised healthcare services) care records [10–13]. However, CMDs are often under-diagnosed in routine primary care data (the so-called 'clinical iceberg' phenomenon), with over half of all depressed patients with clinical symptoms of depression not recognised as such [14,15]. Reasons for this include: individuals with CMDs not visiting their GP [16]; GPs misdiagnosing, or being reticent in diagnosing, CMDs [15]; and GPs increasingly recording symptoms, rather than specific diagnoses [17]. This 'clinical iceberg' may be particularly prevalent among children and adolescents, who may be less likely to visit their GP. Additionally, GPs may fail to identify, or be less willing to diagnose CMDs or prescribe medication to these groups, potentially in part due to a lack of confidence of GPs in identifying and managing CMDs in children and adolescents, low mental health literacy and help-seeking behaviour among these groups, and GP visits being too brief to elicit such sensitive information [18–22]. Primary care physicians frequently refer to secondary care services, such as Child and Adolescent Mental Health Services (CAMHS; [23]), again contributing to the under-reporting of adolescent CMDs in primary care records.

To assess the accuracy of primary care-derived CMD rates, these must be compared against a reference standard [16]. A systematic review in adults found that, relative to a reference standard, specificity is generally high (few false positives) but sensitivity is rather low (many false negatives; [12]). Previous research from the Avon Longitudinal Study of Parents and Children (ALSPAC) compared linked primary care records at age 17/18 against CMDs measured on 1,562 participants via the revised Clinical Interview Schedule (CIS-R) [10]. Using CIS-R as the reference standard, this study found that – similar to findings in adults - sensitivities were low while specificities were high. Together, these findings suggest that primary care data may significantly underestimate the prevalence of CMDs in the population.

Previous UK research has shown that greater symptom severity is the strongest predictor of attending primary care regarding mental health [16]. Other factors, such as age, sex and employment status, also predicted access to primary care, but their contributions were weaker [16]. In contrast, a smaller US study of individuals with depressive symptoms found no demographic differences between those who sought help and those who did not, although symptom severity again predicted help-seeking behaviour [24]. Sociodemographic factors may play a role in access to primary care, recognition of symptoms, and access to treatment, which contribute to continuing health inequalities [25,26]. For instance, a UK study found that both ethnicities other than White British and low socioeconomic position predicted lower rates of CMD detection in primary care records during the maternal period [27]. Even if individuals with a CMD do contact a physician, the

For peer review only - http://bmjopen.bmj.com/site/about/guidelines.xhtml

likelihood of receiving treatment is also dependent on symptom severity, as well as socio-demographic factors [28,29].

Models predicting 'true' CMD status from variables available in primary care records could help to identify the prevalence of individuals with 'missing' CMDs as well as the factors predicting these cases. Previous work has predicted CMDs based on an Australian dataset [30], but did not use primary care records, so its utility may be limited as some relevant factors are unlikely to be present in routine health records (e.g., job satisfaction, social isolation, being a carer, having a partner, etc.). Research using only primary care record data to predict validated measures of CMDs from population-based studies is therefore required.

This study has three aims:

1) Replicate and expand the results of a previous ALSPAC study at age 17/18 (~2,800 participants [10]) by including additional participants with linkage data (~12,000 participants [31,32]), and explore agreement between primary care records and cohort data across multiple time points over adolescence and young adulthood (ages 15-23).
2) Assess the factors impacting rates of CMD identification in primary care records.
3) Construct prediction models, with ALSPAC-measured CMDs as the outcome, to predict CMD status using only primary care data.

METHODS

Study Design and Participants

ALSPAC is a pregnancy-based longitudinal birth cohort which recruited pregnant women in the Bristol area of southwest England with an expected delivery date between 1st April 1991 and 31st December 1992 [33,34]. A total of 14,541 eligible pregnancies were initially recruited into the study, with a total of 14,676 foetuses, resulting in 14,062 live births, of which 13,988 were alive at one year of age. After further waves of post-natal recruitment, as of February 2019 there are a total of 14,901 study child participants enrolled in ALSPAC who were alive at one year [32]. These children and their parents have been followed since birth, with detailed data collected via questionnaires, in-person clinic assessments, and linkage to routine data sets. The study website contains details of all available data through a fully searchable data dictionary and variable search tool: http://www.bristol.ac.uk/alspac/researchers/our-data/. From 2014 onwards (when the study children were aged 22 years) data were collected and managed using REDCap electronic data capture tools hosted at the University of Bristol [35]. We used the STROBE cohort reporting guidelines [36].

When the study children reached legal adulthood (age 18), ALSPAC initiated a postal fair processing campaign to formally re-enrol the children into the study (prior to this parent-based consent was mandatory, although from age 9 children assented to data collection as well) and to simultaneously seek opt-out permission for ALSPAC to link to their health and administrative records [37]. Linkage to primary care records was carried out following this campaign and electronic primary care records have been extracted for nearly 12,000 study children [32]. This linkage is described in more detail in the supplementary material (see also [31]).

In total, 14,731 ALSPAC participants were eligible for our study, comprising all enrolled singletons and twins who were alive at 1 year of age and had not withdrawn consent from the study (data from triplet/quadruplet births were excluded due to confidentiality reasons, as per standard ALSPAC

For peer review only - http://bmjopen.bmj.com/site/about/guidelines.xhtml

protocols). Of this total sample, 13,113 participants were sent fair processing materials, of which 368 (2.8%) dissented to linkage. Primary care records (although not necessarily for the entire time period) were extracted for 11,807 of these individuals (80% of the original 14,731 eligible participants; 90% of the 13,113 sent fair processing materials). Note that there are several dynamic factors that affect inclusion eligibility in these analyses (e.g., study enrolment status and linkage quality to the NHS Person Demographics Service, PDS). Therefore, the numbers reported here may differ from the numbers reported in the ALSPAC primary care linkage data note (currently in preparation).

The current study includes ALSPAC data from multiple time points between the ages of 15 and 23 (table 1), from either clinic or questionnaire data collections. The age 15/16 and 17/18 clinics collected data on both depression and anxiety; at the other time points only depression was assessed. Linked primary care record data coverage decreases with age because the linkage data primarily covers the Bristol area; as many participants moved away as they reached adulthood (e.g. for university or work) they are lost from the linked dataset.

Table 1: Details of ALSPAC data used and coverage with primary care linkage data.

Age (time point)	Measure	# with ALSPAC CMD data	# of these with primary care data (%)
**Age 15/16 (TF3 clinic)**	DAWBA (Depression & anxiety)	5,332	3,663 (68.7%)
**Age 16/17 (CCS questionnaire)**	SMFQ (Depression only)	4,950	3,213 (64.9%)
**Age 17/18 (TF4 clinic)**	CIS-R (Depression & anxiety)	4,534	3,084 (68.0%)
**Age 18/19 (CCT questionnaire)**	SMFQ (Depression only)	3,302	1,982 (60.0%)
**Age 21/22 (YPA questionnaire)**	SMFQ (Depression only)	3,283	1,298 (39.5%)
**Age 22/23 (YPB questionnaire)**	SMFQ (Depression only)	3,896	1,325 (34.0%)

DAWBA: Development and Well-Being Assessment; *SMFQ*: Short Mood and Feelings Questionnaire; *CIS-R:* Clinical Interview Schedule – Revised.

ALSPAC data

At the age 15/16 clinic, depression and anxiety were assessed using the Development and Well-Being Assessment (DAWBA) interview [38], which identifies several psychiatric diagnoses in children and adolescents (based on International Classification of Diseases-10 (ICD-10) and Diagnostic and Statistical Manual of Mental Disorders fourth edition (DSM-IV) criteria). Here, in addition to a diagnosis for depression by itself, we defined CMDs as a diagnosis of depression and/or any anxiety disorder (generalised anxiety disorder, panic disorder, agoraphobia, social phobia and specific phobias).

At the 17/18 clinic, depression and anxiety were assessed using a self-administered computerised CIS-R questionnaire [39]. As with DAWBA, CIS-R can be used to assign ICD-10 diagnoses of depression and anxiety disorders [40]. Here, anyone diagnosed with mild, moderate or severe depression was classified as having depression, while a diagnosis of CMD was defined having

For peer review only - http://bmjopen.bmj.com/site/about/guidelines.xhtml

depression and/or an anxiety disorder (generalised anxiety disorder, mixed anxiety and depression, panic disorders and phobic disorders).

At the other ages (16/17, 18/19, 21/22 and 22/23 questionnaires), depression was assessed using a self-administered Short Mood and Feelings Questionnaire (SMFQ), a 13-item questionnaire assessing depressive symptoms over the past two weeks [41]. Total SMFQ scores range between 0-26, with a score of 12 or more frequently used as a diagnosis of depression [42]. Although there are problems of inaccuracy with using cut-offs from questionnaires as screening tools for depression [43], using ALSPAC data the validity of the SMFQ during childhood and adolescence was found to be high when compared against ICD-10-derived depression diagnoses from CIS-R at age 17/18 [44]. Only participants who answered all 13 SMFQ questions were included in the analyses.

To compare sociodemographic differences between those with and without linked primary care data and to explore whether demographic factors impact rates of identification in primary care records, several variables measured during pregnancy or at birth and known to be predictive of non-response in ALSPAC were utilised [33,34]. These include child sex; maternal age, home ownership status; marital status and parity; parental education levels; and child ethnicity. Additional variables used for aims 2 and 3 are discussed below.

Electronic primary care data

The linked primary care data comprised Read codes V.2 (5 byte), along with associated dates. Read codes are a comprehensive list of standardised clinical terms used by healthcare professionals within the UK National Health Service to record clinical information (they have since been replaced by 'SNOMED CT' codes, but our data contained Read codes as they pre-dated this change). Read codes relevant to diagnosis, symptoms or treatment (antidepressants, anxiolytics and hypnotics) of depression or anxiety (including phobic disorders) were extracted [10,11]. These were combined to produce three definitions of depression and CMDs (table 2). Based on previous research [10], these were chosen to include the definitions with the lowest sensitivity ('current diagnosis, treated'), the highest sensitivity ('current diagnosis or treatment or symptoms'), and an intermediate sensitivity which is also the most straightforward to extract from primary care records ('current diagnosis'). 'Current' diagnoses, symptoms or treatment were defined as being 6 months either side of the age the study child attended the clinic or completed the questionnaire and 'historical' as having occurred at least 6 months prior to the age at the clinic/questionnaire. Note that treatment does not include psychological therapies, even though these are the recommended first line of treatment for adolescents, as these therapies are mainly given by specialist secondary mental health services and may not be noted in primary care records. Read codes were used to identify referrals to mental health services. A list of the Read codes used are provided in supplementary table S1.

Table 2: Details of the multiple definitions of 'depression' and 'CMD' derived from the primary care data.

Definition	Description
**Current diagnosis**	Current diagnosis of depression/CMD
**Current diagnosis, treated**	Current diagnosis of depression/CMD and currently receiving treatment
**Current diagnosis or treatment or symptoms**	Current diagnosis or symptoms or treatment for depression/CMD

For peer review only - http://bmjopen.bmj.com/site/about/guidelines.xhtml

Additional data were extracted to predict identification in primary care records and for the prediction models. These primary care variables may be associated with our outcomes of interest, and included: average annual number of GP consultations and prescriptions at the relevant time point; current and historical somatic and general symptoms (defined in supplementary table S1); referral to mental health services; common chronic health conditions (asthma and eczema); other mental health conditions (eating disorders, ADHD, conduct disorder, autistic spectrum disorder, alcohol and drug abuse, schizophrenia, bipolar disorder and psychosis); family and personal history of depression and mental health issues; self-harm; and smoking status (described in more detail below).

To ensure that only individuals with primary care data at the relevant time points were included, inclusion criteria were: i) having associated linkage data; ii) having primary care data for at least 6 months after their clinic visit or questionnaire completion (based on GP registration dates); and iii) first appearing in the primary care records a minimum of 18 months prior to their clinic visit or questionnaire completion (allowing a 6 month window for 'current' data, plus a whole year previous for 'historical' data).

Statistical Analysis

For each primary care definition and at each time point, sensitivity, specificity and positive and negative predictive values were calculated separately for predicting ALSPAC-derived measures of depression and CMDs (if measured). Exact 95% confidence intervals were derived using binomial probabilities.

We then explored factors associated with identification of CMDs/depression in primary care records for individuals diagnosed in the ALSPAC data. As primary care diagnosis numbers were low, we used the definition with the highest sensitivity ('current diagnosis or symptoms or treatment'). Univariate logistic regression was used to explore whether each covariate was associated with identification. The variables used to predict identification were a combination of ALSPAC and primary care data (for a full list see table S2). These identification analyses were repeated for each timepoint, separately for both depression and CMD (if measured).

Finally, lasso (Least Absolute Selection and Shrinkage Operator) models were used at each time point to assess the combination of variables from primary care data which best predicted ALSPAC-derived depression/CMDs. Lasso models apply a lambda weight which constrains weakly-predictive variables falling below this value to zero, while also shrinking remaining non-zero coefficients towards zero. This results in sparse models which minimise over-fitting, and can subsequently be used for out-of-sample prediction [45,46]. Ten-fold cross-validation was used for all lasso models and visual inspection of the cross-validation plots were performed to assess that the optimal lambda value had been selected.

We randomly split our sample into 60% training and 40% validation samples, and then compared the deviance ratios for each to inspect how well the training model performed when predicting depression/CMDs in the validation sample. The deviance ratio is a goodness-of-fit statistic generally between 0 and 1 comparable to R^2, but for non-linear models, indicating the fraction of deviance explained relative to the null constant-only model (with values closer to 1 indicating better model fit; see page 33 of [45]). In-sample deviance ratios refer to results from the 60% training sample, while out-of-sample deviance ratios refer to results from the 40% validation sample (note that as the coefficients derived from the training sample are used in the validation sample, it is possible to

For peer review only - http://bmjopen.bmj.com/site/about/guidelines.xhtml

observe negative deviance ratios, indicating that the coefficients are *worse* at predicting the outcome than the null model). Logistic lasso models were used with ALSPAC-derived depression or CMDs at each time point as the outcome variable, and all variables in table S3 as predictors.

To assess whether these models, which utilise all the available information held in primary care records, increase model fit relative to just the primary care diagnosis/symptoms/treatment data, we compared these models against: i) a prediction model which just contained 'current diagnosis' as a predictor variable; and ii) a prediction model which included 'current diagnosis', 'current symptoms' and 'current treatment' as predictor variables. In- and out-of-sample deviance ratios of these models were compared to assess model fit.

For each time point, from the models utilising all the available primary care data we also calculated the predicted probabilities of receiving a depression or CMD diagnosis (with a threshold of >50% probability to define diagnosis) in the 40% validation sample, and compared sensitivities and specificities derived from these prediction models against the three definitions using the raw primary care data (table 2). All analyses were conducted using Stata v.16.0.

Patient and Public Involvement Statement

ALSPAC has an advisory panel of >30 participants who meet bimonthly to advise on study design, methodology and acceptability. Investigations were carried out into participants' understanding of and feelings towards the acceptability of linking to health and administrative data and, in particular, to accessing sensitive health information such as data on mental and sexual health. This information was obtained through a qualitative study and through discussions with ALSPAC's this advisory panel. However, the advisory panel were not involved in the specific study reported here. ALSPAC communicates with participants via regular newsletters and has an active website and social media presence.

RESULTS

Demographics and Linkage Data Coverage

Table 1 shows numbers with both linkage and ALSPAC data at each time point (the reasons individuals who have ALSPAC data, but do not have linkage data, are provided in table S4). The proportion of unlinked records increases with age, most likely because these individuals left the area as they became adults.

Comparisons between those with ALSPAC data who do and do not have primary care data are presented in tables 3 (for age 15/16 and 22/23 time points) and S5 (for all other time points). There are some differences, particularly in terms of socio-economic position (e.g., less likely to have primary care data if higher parental education levels), but little difference in terms of sex. At later time points, participants with more GCSEs or equivalents are less likely to have primary care data. Few differences in depression/CMD diagnosis are apparent between these two groups. With the exception of CMD/depression diagnoses (which increases with age), differences in demographics across the time points are minimal, although the proportion of females with ALSPAC data does increase over time.

Figure 1 gives the proportions with a current diagnosis of depression/CMD in the primary care data comparing those who did to those who did not complete the ALSPAC clinic or questionnaire. Those

For peer review only - http://bmjopen.bmj.com/site/about/guidelines.xhtml

with ALSPAC data are more likely to have a current CMD diagnosis, particularly at the later time points. For depression, those with ALSPAC data are slightly more likely to have a primary care diagnosis at ages 21/22 and 22/23 but there are no differences at earlier time points.

For peer review only

For peer review only - http://bmjopen.bmj.com/site/about/guidelines.xhtml

Table 3: Demographics at the age 15/16 clinic and 22/23 questionnaire time points. Of those with ALSPAC data, the table compares those who have primary care data against those who do not. For categorical variables cells are counts and percentages. For continuous variables cells are means and standard deviations. Note also that the denominators vary as the variables come from different data sources, with different levels of completeness. As the demographics are broadly similar across all time points, only the first and last time point are presented here (see table S5 for all other time points).

	**Age 15/16 (TF3 clinic) – DAWBA (CMDs and Depression)**		**Age 22/23 (YPB questionnaire) – SMFQ (Depression)**	
	**Primary care data (*n*=3663)**	**No primary care data (*n*=1669)**	**Primary care data (*n*=1325)**	**No primary care data (*n*=2571)**
**Sex**				
**Male**	1748 (47.7%)	782 (46.9%)	474 (35.8%)	868 (33.8%)
**Female**	1915 (52.3%)	887 (53.2%)	851 (64.2%)	1703 (66.2%)
**Maternal age at child's birth**	29.2 (4.6)	29.3 (4.6)	29.2 (4.5)	29.6 (4.4)
**Mother's home ownership status**				
**Owned/ Mortgaged**	2878 (85.3%)	1292 (83.8%)	998 (84.2%)	2022 (85.5%)
**Rented**	137 (4.1%)	90 (5.8%)	58 (4.9%)	121 (5.1%)
**Council/Housing Association**	281 (8.3%)	111 (7.2%)	100 (8.4%)	151 (6.4%)
**Other**	79 (2.3%)	48 (3.1%)	30 (2.5%)	70 (3%)
**Mother's marital status**				
**Never married**	460 (13.5%)	211 (13.6%)	152 (12.6%)	262 (11%)
**Single/Divorced**	147 (4.3%)	83 (5.3%)	50 (4.2%)	105 (4.4%)
**First marriage**	2607 (76.5%)	1149 (74.0%)	918 (76.2%)	1851 (77.7%)
**2nd/3rd marriage**	194 (5.7%)	110 (7.1%)	85 (7.1%)	164 (6.9%)
**Mother's parity**				
**0**	1623 (48.1%)	787 (51.3%)	560 (47.0%)	1155 (49.1%)
**1**	1201 (35.6%)	507 (33.1%)	413 (34.7%)	815 (34.7%)
**2 or more**	554 (16.4%)	240 (15.7%)	218 (18.3%)	381 (16.2%)
**Mother's highest education level**				
**O level/lower**	1884 (55.2%)	765 (49.3%)	748 (62.8%)	1071 (45.5%)
**A level**	920 (27.3%)	476 (30.7%)	280 (23.5%)	770 (30.6%)
**Degree**	565 (16.8%)	312 (20.1%)	164 (13.8%)	562 (23.9%)
**Father's highest education level**				
**O level/lower**	1428 (46.2%)	559 (39.3%)	550 (50.5%)	795 (35.9%)
**A level**	947 (30.6%)	437 (30.6%)	348 (32%)	653 (29.5%)

For peer review only - http://bmjopen.bmj.com/site/about/guidelines.xhtml

**Degree**	716 (23.2%)	429 (30.1%)	191 (17.5%)	767 (34.6%)
**Child ethnicity**				
**White**	3179 (95.9%)	1466 (95.8%)	1138 (96.8%)	2237 (96.2%)
**Non-white**	137 (4.1%)	65 (4.3%)	38 (3.2%)	88 (3.8%)
**# GCSEs (or equivalents)**	7.3 (3.6)	7.5 (3.6)	7.3 (3.5)	8.3 (3.2)
**ALSPAC depression diagnosis**				
**No**	3606 (98.4%)	1638 (98.1%)	1103 (83.2%)	2180 (84.6%)
**Yes**	57 (1.6%)	31 (1.9%)	223 (16.8%)	398 (14.5%)
**ALSPAC common mental disorder (CMD) diagnosis**				
**No**	3540 (96.6%)	1622 (97.2%)	-	-
**Yes**	123 (3.4%)	47 (2.8%)	-	-

DAWBA: Development and Well-Being Assessment; *CMDs:* Common mental disorders; *SMFQ*: Short Mood and Feelings Questionnaire.

For peer review only

Sensitivity, Specificity and Predictive Values

We focused first on the age 17/18 clinic data (table 4), the results of which were broadly consistent with previous ALSPAC analyses [10]. At this age, 243/3,084 participants (7.9%) were diagnosed as depressed using the ALSPAC CIS-R data, while 455 (14.8%) met the criteria for ALSPAC diagnosis of CMD. Using the various primary care definitions, the number of individuals diagnosed as depressed ranged from a minimum of 20 (0.7%) using a definition of 'current diagnosis, treated', to a maximum of 122 (4%) using a definition of 'current diagnosis or symptoms or treatment'. Thus, the sensitivity of each primary care definition to predict ALSPAC-derived CMDs was low, ranging from 3.7% to 24.3%. Specificity was higher (all > 97.8%), as was the negative predictive values (NPVs; all > 92%), while positive predictive values (PPVs) ranged between 45% and 58.4%. For the primary care CMD data, numbers diagnosed ranged from a minimum of 29 (0.9%) to a maximum of 171 (5.5%). Sensitivity (range: 3.5-19.1%) and specificity (all > 96.8%) to predict ALSPAC-derived CMDs were marginally lower compared to depression at this age, while each PPV was slightly higher (range: 47.4-55.2%) and NPV lower (range: 85.6-87.4%).

Similar results were found for the age 15/16 clinic using the DAWBA measure (table S7), albeit with fewer depression and CMD diagnoses (in both primary care and ALSPAC) and lower sensitivities. The comparison between ALSPAC SMFQ questionnaire data and primary care data at ages 16/17, 18/19, 21/22 and 22/23 are displayed in supplementary tables S8 to S11, and were similar to those using DAWBA (age 15/16 clinic) and CIS-R (age 17/18 clinic), with relatively high specificity but low sensitivity for all primary care definitions of depression when predicting ALSPAC-derived depression. Sensitivity increased with age, while specificity decreased (figure 2).

Identification of CMDs/Depression Cases in Primary Care Records

Next, we explored the factors associated with identification as a case from the primary care data in those with an ALSPAC-derived diagnosis. Results are presented in full in table S12 (giving odds ratios and 95% confidence intervals for all analyses) and figure S1 (providing a graphical summary of key results over each time point). There are few consistent associations of sociodemographic factors (parental education, child sex, child education, etc.) with being identified as a case in the primary care records. Primary care case identification was more likely in participants with greater symptom severity. Some primary care covariates (e.g., smoking status, eating disorder and other mental health issues) were associated with higher rates of primary care case identification at younger ages, but had weaker associations at later ages. Others (somatic and general symptoms, higher consultation/prescription rates, referrals to mental health services and self-harm status) were consistently associated with higher rates of primary care case identification. Due to the low numbers diagnosed as having CMD or depression at the age 15/16 clinic, both from the DAWBA assessment and from primary care records, results from this time point should be treated with caution.

For peer review only - http://bmjopen.bmj.com/site/about/guidelines.xhtml

Table 4: Depression and CMD diagnoses based on the CIS-R (clinical interview schedule – revised) data from the age 17/18 TF4 clinic against various definitions derived from the primary care data at this age (*n*=3,084). This table also includes sensitivities, specificities, positive predictive values (PPV) and negative predictive values (NPV) for the depression and common mental disorder (CMD) diagnoses based on the CIS-R data from this clinic. In these analyses we are treating the ALSPAC data as the reference standard.

		CIS-R Depression			CIS-R CMD		
**Primary care definition**		**No**	**Yes**	**Total**	**No**	**Yes**	**Total**
**Current diagnosis**	No	2,825	229	3,054	2,599	428	3,027
	Yes	16	14	30	30	27	57
**Sensitivity**		5.8% (3.2; 9.5)			5.9% (3.9; 8.5)		
**Specificity**		99.4% (99.1; 99.7)			98.9% (98.4; 99.2)		
**Positive Predictive Value**		46.7% (28.3; 65.7)			47.4% (34.0; 61.0)		
**Negative Predictive Value**		92.5% (91.5; 93.4)			85.9% (84.6; 87.1)		
		**No**	**Yes**	**Total**	**No**	**Yes**	**Total**
**Current diagnosis, treated**	No	2,830	234	3,064	2,616	439	3,055
	Yes	11	9	20	13	16	29
**Sensitivity**		3.7% (1.7; 6.9)			3.5% (2.0; 5.6)		
**Specificity**		99.6% (99.3; 99.8)			99.5% (99.2; 99.7)		
**Positive Predictive Value**		45% (23.1; 68.5)			55.2% (35.7; 73.6)		
**Negative Predictive Value**		92.4% (91.4; 93.3)			85.6% (84.3; 86.9)		
		**No**	**Yes**	**Total**	**No**	**Yes**	**Total**
**Current diagnosis or symptoms or treatment**	No	2,778	184	2,962	2,545	368	2,913
	Yes	63	59	122	84	87	171
**Sensitivity**		24.3% (19.0; 30.2)			19.1% (15.6; 23.0)		
**Specificity**		97.8% (97.2; 98.3)			96.8% (96.1; 97.4)		
**Positive Predictive Value**		48.4% (39.2; 57.6)			50.9% (43.1; 58.6)		
**Negative Predictive Value**		93.8% (92.9; 94.6)			87.4% (86.1; 88.6)		

Predicting ALSPAC CMDs/Depression from Primary Care Records

The in-sample deviance ratios, fitted on the 60% training sample, and the out-of-sample deviance ratios, fitted on the 40% validation sample, for each time point are displayed in table 5. In general, in-sample deviance ratios are quite low (8.3 to 14.6%). Out-of-sample deviance ratios are lower (-1.3 to 12.6%) but do increase with age. The penalised coefficients from these prediction models are presented in table S13, with full models to estimate predicted probabilities given in table S14. Many factors from the primary care data consistently predicted ALSPAC CMD/depression diagnoses across many time points, including: being female, a history of self-harm, number of GP consultations, referral to mental health services and historical and/or current depression diagnoses/symptoms/treatment. Several associations were time point specific, occurring in only one or two models (e.g., smoking at TF4 depression and CCS, eczema for TF4 depression, conduct disorder at CCS, etc.). These coefficients should not be interpreted causally, especially is there is high collinearity between variables (as is likely to be present here given that many variables measure similar constructs).

For peer review only - http://bmjopen.bmj.com/site/about/guidelines.xhtml

Table 5: In-sample and out-of-sample deviance ratios predicting depression and common mental disorders (CMDs) for each time point using all the available primary care data. Deviance ratios are taken from logistic cross-validation lasso prediction models.

	**Age 15/16 TF3 clinic (*n* = 3,663)**		**Age 16/17 CCS quest. (*n* = 3,213)**	**Age 17/18 TF4 clinic (*n* = 3,084)**		**Age 18/19 CCT quest. (*n* = 1,982)**	**Age 21/22 YPA quest. (*n* = 1,298)**	**Age 22/23 YPB quest. (*n* = 1,325)**
	Dep.	CMD	Dep.	Dep.	CMD	Dep.	Dep.	Dep.
**In-sample deviance ratio (60% training sample)**	9.9%	8.4%	8.3%	14.6%	9.2%	9.0%	11.7%	13.4%
**Out-of-sample deviance ratio (40% validation sample)**	-1.3%	2.9%	4.3%	7.8%	7.8%	8.0%	12.6%	9.1%

For all time points other than age 15/16 clinic depression, the 'full' prediction model (based on the set of all primary care variables; table S3) performed better than both the 'diagnosis only' and 'diagnosis/symptoms/ treatment' models for both in-sample and out-of-sample prediction (table S15).

Sensitivities from these prediction models were marginally higher than for definitions of 'current diagnosis' and 'current diagnosis with treatment', but lower than the 'current diagnosis or treatment or symptoms' sensitivities. However, the specificities of the prediction models were greater than the 'current diagnosis or treatment or symptoms' definition, and on par with the stricter definitions based on 'current diagnosis' or 'current diagnosis with treatment' (table S16). These prediction models therefore appear to more accurately detect cases of depression/CMD compared to these more stringent definitions from the primary care records, while also avoiding many of the false negatives associated with broader definitions (such as 'current diagnosis or treatment of symptoms'). However, sensitivities from these prediction models are still rather low, ranging between 3.5% and 16.3% (all specificities are >98%).

DISCUSSION

This study compared primary care data against validated measures of CMDs at multiple time points during adolescence and young adulthood. Taking ALSPAC data as the reference standard, our results demonstrate that, regardless of how CMDs are defined from primary care records, sensitivities are low across all ages (range: 1.6 to 34%). However, detection of CMDs in primary care records does improve with age. Specificities were high, with most above 95%. This suggests that the primary care data for CMDs is likely to contain many 'false negatives' but few 'false positives', as documented previously [12].

This study also explored the factors predicting identification of "cases" (as identified in ALSPAC data) in primary care data. Consistent with previous research [16,24], the strongest predictor was symptom severity, with individuals displaying more severe symptoms increasingly likely to be correctly classified. A history of CMDs, as well as increased rates of other mental health issues, somatic or general symptoms and engagement with primary care services (consultation and

For peer review only - http://bmjopen.bmj.com/site/about/guidelines.xhtml

prescription rates), also predicted greater primary care identification rates. Many adolescents receive mental health care via specialised secondary care services, rather than through their GP, and this is reflected in referrals to secondary mental health services also being associated with higher identification rates. Unlike for the wider adult population, we found little evidence that sociodemographic factors were consistently associated with case identification in primary care records for adolescents and young adults [27,47].

Finally, this paper also presented a series of prediction models, which can be used by epidemiologists with access only to primary care data to predict CMDs in individuals who may not be formally diagnosed by a GP. Although the variance explained by these models is quite low, these models demonstrate that the inclusion of additional covariates from primary care records improved model fit, relative to models that contained only current diagnosis, symptoms or treatment. Out-of-sample prediction rates increased with age, suggesting that these models better predict depression/CMDs in young adulthood compared to adolescence. This is perhaps not surprising, given that rates of diagnoses from primary care data are low in adolescence and increase with age. However, comparison of the predicted sensitivities and specificities from these prediction models indicates that the improvement in detection of depression/CMDs relative to the primary care record data based on diagnosis, symptoms and treatment is minimal. We also acknowledge that these prediction models were only validated internally using ALSPAC data; before being used more widely, these models should be calibrated and validated externally using independent datasets from different populations (see, e.g., [48]).

Strengths and Limitations

A strength of this study is that it uses established methods to systematically define CMDs from primary care records [10,11], allowing cross-study comparisons. This study uses a larger sample than a previous study using ALSPAC adolescent data [10], and extends the age range assessed to adolescence through to early adulthood. This permits a broader view of how both ALSPAC-derived and primary care-derived CMD rates change with age, how sensitivities and specificities vary over the transition to adulthood, and how prediction models alter over this developmental stage. A further strength is that this study also uses a large, deeply-phenotyped cohort, with depression and CMD measured at multiple time points using validated instruments.

This study has several limitations. The primary care data coding used may miss crucial information: possible diagnoses and symptoms may be noted within the ‘free text’ of routine electronic records [12], which are generally not available for research purposes [49]. The primary care data only records pharmacological treatments prescribed by the GP, rather than psychological treatments provided by secondary care services. As the first line of treatment for adolescents is often psychological therapies, especially for mild depression [50]), this may partially explain the lower sensitivities at younger ages. Although we included CAMHS referrals in our identification analyses and prediction models, this is still likely to underestimate the true prevalence of adolescent CMDs as only around one-third of children with a mental health disorder are referred to CAMHS [23]. Further, fewer than half of referrals to CAMHS in the UK are from a GP [51], with school nurses, self-referrals and other routes to CAMHS possible. It is important to reinforce here that the aim of this paper was to detect and predict participants with CMDs using just primary care data, not to identify all potential individuals with a CMD from routine health records. For instance, it is possible that some participants may be ‘detected’ as having a CMD and referred to secondary healthcare services (e.g., CAMHS) without having a CMD diagnosis in their primary care records if they did not present to their

For peer review only - http://bmjopen.bmj.com/site/about/guidelines.xhtml

GP and were referred to secondary care services by another route. As we were interested in using just primary care records, these individuals would not appear as having a CMD in our data. Implications of this, and methods of improving detection rates when using routine health records, are discussed in the 'Implications and recommendations' section below.

It is also possible that we have omitted other clinically-relevant conditions noted in primary care record Read codes, such as additional personality disorders (e.g., schizoid, borderline, obsessive-compulsive, etc.), which are often associated with CMDs and may have improved the accuracy of our prediction models [52]. However, personality disorders are frequently difficult to diagnose in primary care settings [53], and hence often not diagnosed by GPs [54]. Additionally, many personality disorders, or proxies thereof – such as conduct disorders, eating disorders, psychosis, schizophrenia, self-harm, frequency of consultations, referral to secondary health services and CMDs themselves [52–55] – were included in the present study, yet were not consistent predictors of ALSPAC-derived CMDs (Table S13). Additionally, for all participants over the whole period covered in the study, there were approximately 14,500 Read codes relating to diagnoses of a mental health disorder, of which ~8,000 were for CMDs and a further ~4,200 were captured by other measured conditions (ADHD, eating disorders, conduct disorder, etc.). In total, there were only 220 diagnosis codes for either personality disorder or obsessive-compulsive disorder, which related to 42 individuals. Omitting these additional personality disorders is therefore unlikely to substantially impact our conclusions or improve our prediction models. While this may be an important consideration if personality disorders were more commonly-identified in the primary care records, we do not believe that this is a major limitation of this research.

A second limitation is that the linkage is primarily Bristol-based. As the cohort reaches adulthood they are more likely to move away from Bristol; as such, the proportion with linkage data drops from approximately two-thirds before age 18 to roughly one-third after this age. In addition to the resulting loss of statistical power and precision, there is also the potential for selection bias if those with linkage data systematically differ from those without [56,57]. At each time point, of those with ALSPAC data there are differences between those with and without linked primary care data in terms of socioeconomic position (e.g., higher maternal education levels are associated with lower probability of having linkage data). Although differences in ALSPAC-derived CMDs appear minimal comparing those with vs without primary care data (table 3), it is possible that primary care data may differ between these groups. This may limit the generalisability of our prediction models; for example, compared to the whole ALSPAC cohort our sample with primary care data is biased towards those with a lower socioeconomic position, who may be less likely to attend GP appointments [58]. However, as in the wider ALSPAC cohort respondents tend to be from higher socioeconomic strata [34], the impact of linkage data biased towards lower-SEP (socioeconomic position) individuals on generalisability is uncertain. Comparing the primary care-derived CMD status of those with and without ALSPAC data we observe few differences in terms of depression or CMDs at younger ages but, in adulthood, CMDs (although less so for depression) appear more prevalent among those with ALSPAC data (figure 1). One possible interpretation of this is that it reflects the demographics of ALSPAC respondents, as being female is associated with continued ALSPAC participation [34], and females are at greater risk of CMDs [59,60]. When adjusting for sex these effects were somewhat attenuated, although participants with ALSPAC data at the 21/22 and 22/23 questionnaire time points were still more likely to have a primary care-derived CMD (table S17). Inclusion of parental education (a proxy for SEP), which may also predict both continued ALSPAC participation and mental health, did not further diminish this effect. The selection pressures associated with having continued primary care linkage data in ALSPAC are likely to be complex and require further investigation to assess the potential for selection bias when using this data.

For peer review only - http://bmjopen.bmj.com/site/about/guidelines.xhtml

A related limitation is that as the research is specifically Bristol-based, generalisability to other populations, both in the UK and elsewhere, should be made with caution. For instance, the ALSPAC cohort is not representative of the UK national population, as ALSPAC contains a greater proportion of white and higher SEP individuals [34], which is likely to shape health-seeking behaviour and GP engagement rates [26,27]. A further issue regarding generalisability is that the data in adolescence was collected between 2006 and 2011. Given the large shift in societal values towards increased visibility, awareness and understanding of mental health issues over the past few years, this may impact both GP decision-making and adolescents' health-seeking behaviour, potentially affecting diagnosis rates in this age group. Additional research is necessary to explore this among existing adolescents. As such, these models should be calibrated before use in other areas or calendar times.

A third limitation is the small numbers of individuals with CMD/depression in ALSPAC, especially at younger ages (and particularly the age 15/16 clinic data). This may explain why we failed to detect consistent sociodemographic differences in case identification, contrary to previous research with larger samples [16,27,47]. Larger studies are required to explore sociodemographic associations with identification in primary care records in greater detail, which – if present – are likely to be weaker than the effects of symptom severity [16,24]. In addition to the relatively small sample size, one potential reason for the lack of SEP gradient could be that SEP is based on parental SEP at the time of the study child's birth. Although parental SEP and child health outcomes are frequently correlated, this association is strongest in early childhood and tends to weaken with age [26]. Assessing the individual's SEP directly, particularly in early adulthood, may reveal these health inequalities. A further consequence of this paucity of diagnoses in adolescence is that we were not able to explore CMDs in early adolescence, despite many CMDs having their onset prior to age 15 years [5]; extending and replicating these results in a larger study which includes early adolescents would be an interesting avenue for future research.

A further limitation is that we have taken the ALSPAC data as the 'reference standard'. These measures may over-diagnose the presence of CMDs, especially in 'borderline' cases with less severe symptoms who may not visit their GP, thus increasing the number of false positives in the ALSPAC data. Although all of the instruments used in ALSPAC have been validated and are routinely used to screen for depression and CMDs [38,39,41,44], previous studies have demonstrated that these questionnaire-based tools can provide quite divergent diagnoses of mental health conditions compared to standard clinical interviews (e.g., CIS-R; [40]). Additionally, apparent false negatives may also appear in the ALSPAC data if individuals are successfully receiving treatment to alleviate their CMD symptoms; in these cases, individuals would be diagnosed as having CMD via primary care records, but not via ALSPAC data.

Implications and Recommendations

Consistent with previous research [12], this study has demonstrated that the rate of false negatives for CMDs in adolescents and young adults in routine primary care data is high. Thus, additional sources of information need to be utilised when working with routine health data. As fewer than half of referrals to CAMHS are from GPs [51], using linkage data from CAMHS and other secondary mental health care services would likely increase detection rates. This would appear particularly important for adolescents, as the sensitivities at this age are much lower than in early adulthood. However, as CAMHS is over-subscribed, often only severe cases are accepted, potentially biasing these sources towards those with more severe CMD symptoms. Additionally, even in early adulthood sensitivities are still rather low (maximum 34% at age 21/22), suggesting that additional

For peer review only - http://bmjopen.bmj.com/site/about/guidelines.xhtml

information is required to correctly identify CMDs in linkage data. One potential source of information is from the free-text fields in primary care records, which are not usually made available for research purposes [12]. However, although evidence suggests that using free text data can improve detection of medical conditions more generally [61], the current evidence for CMDs – albeit limited to a small number of studies – suggests their inclusion only marginally improves detection rates [12]. Therefore, in addition to making use of more data sources, we also need better case detection at the primary care level to identify these "invisible" cases who have a CMD but are not currently recorded as such in primary care records. As more severe CMD cases are more likely to be detected in primary care records, these missing cases are likely to present with milder CMD symptoms. Methods to improve GP detection of CMDs include: increasing GP confidence when identifying child and adolescent CMDs [22]; additional support and screening for at-risk groups (e.g., after adverse life events), particularly in childhood and adolescence, to aid early detection of CMDs, as per best-practice guidelines [50,62]; and further training and resources for GPs to aid identification and management of mental health issues [20,21].

Conclusion

We have demonstrated how routine electronic primary care data can be used with cohort study data to estimate the size of the 'clinical iceberg' of undetected CMDs in primary care data throughout adolescence and early adulthood, and to describe the characteristics of those less likely to be identified as cases in primary care records. Although overall sensitivities were low, both sources of data accurately predicted individuals with more severe CMD symptoms. The number of individuals diagnosed as having a CMD, and the correspondence between ALSPAC and primary care data, increased with age. Additional sources of data – e.g., from secondary care services such as CAMHS, or from free text fields – might be required to determine CMD prevalence more accurately, particularly in adolescence. Development of further prediction models may improve estimation of prevalence of CMDs from primary care records and help target interventions to individuals with CMDs who would otherwise not be identified as cases in primary care records. This should be conducted in tandem with methods to improve case detection of CMDs among adolescents and young adults by primary care clinicians.

For peer review only

Acknowledgements

We are extremely grateful to all the families who took part in this study, the midwives for their help in recruiting them, and the whole ALSPAC team, which includes interviewers, computer and laboratory technicians, clerical workers, research scientists, volunteers, managers, receptionists and nurses.

Author Contributions

RPC, KT, SLP, KW, GS and JD conceived and designed the study. RPC processed the primary care data. DS performed the statistical analyses and drafted the manuscript. RPC, KT, SLP, KW, GS and JD contributed to the interpretation of the results. All authors commented on the draft, and have read and approved the final version of the manuscript.

Funding

This work was funded by the Medical Research Council (MRC grant number: MC_PC_17210). The UK Medical Research Council and Wellcome (Grant ref: 217065/Z/19/Z) and the University of Bristol provide core support for ALSPAC. A comprehensive list of grants funding is available on the ALSPAC website http://www.bristol.ac.uk/alspac/external/documents/grant-acknowledgements.pdf); collection of ALSPAC CMD data was funded by the NIH (grant references 5R01MH073842-04 and PD301198-SC101645; for the TF3 DAWBA and YPB MFQ data), Wellcome Trust (grant reference 08426812/Z/07/Z; for the TF4 CIS-R data), and a joint Wellcome Trust and MRC grant (grant reference 092731; for the CCS, CCT and YPA SMFQ data). This publication is the work of the authors and Daniel Smith and Rosie Cornish will serve as guarantors for the contents of this paper.

Competing interests

None declared.

Data availability statement

Information about access to ALSPAC data is given on the ALSPAC website (http://www.bristol.ac.uk/alspac/researchers/access/).

Ethics approval

Ethical approval for the study was obtained from the ALSPAC Ethics and Law Committee and the Local Research Ethics Committees (NHS Haydock REC: 10/H1010/70). Informed consent for the use of data collected via questionnaires and clinics was obtained from participants following the recommendations of the ALSPAC Ethics and Law Committee at the time.

Supplementary material

Supplementary material supporting this manuscript is available as an online supplement.

For peer review only - http://bmjopen.bmj.com/site/about/guidelines.xhtml

References

1 Ferrari AJ, Charlson FJ, Norman RE, *et al.* Burden of Depressive Disorders by Country, Sex, Age, and Year: Findings from the Global Burden of Disease Study 2010. *PLoS Med* 2013;**10**. doi:10.1371/journal.pmed.1001547

2 Pearson RM, Carnegie RE, Cree C, *et al.* Prevalence of Prenatal Depression Symptoms Among 2 Generations of Pregnant Mothers: The Avon Longitudinal Study of Parents and Children. *JAMA Netw open* 2018;**1**:e180725. doi:10.1001/jamanetworkopen.2018.0725

3 Collishaw S, Maughan B, Natarajan L, *et al.* Trends in adolescent emotional problems in England: A comparison of two national cohorts twenty years apart. *J Child Psychol Psychiatry Allied Discip* 2010;**51**:885–94. doi:10.1111/j.1469-7610.2010.02252.x

4 Lewinsohn PM, Clarke GN, Seeley JR, *et al.* Major Depression in Community Adolescents: Age at Onset, Episode Duration, and Time to Recurrence. J. Am. Acad. Child Adolesc. Psychiatry. 1994;**33**:809–18. doi:10.1097/00004583-199407000-00006

5 Patel V, Flisher AJ, Hetrick S, *et al.* Mental health of young people: a global public-health challenge. *Lancet* 2007;**369**:1302–13. doi:10.1016/S0140-6736(07)60368-7

6 Green H, McGinnity A, Meltzer H, *et al.* Mental health of children and young people in Great Britain, 2004. 2005. doi:10.1037/e557702010-001

7 McManus S, Meltzer H, Brugha T, *et al.* Adult psychiatric morbidity in England, 2007: Results of a household survey. 2009. doi:10.13140/2.1.1563.5205

8 Bhatia SK, Bhatia SC. Childhood and adolescent depression. *Am Fam Physician* 2007;**75**:73–80. doi:10.1192/bjp.153.4.476

9 Harvey SB, Henderson M, Lelliott P, *et al.* Mental health and employment: Much work still to be done. *Br J Psychiatry* 2009;**194**:201–3. doi:10.1192/bjp.bp.108.055111

10 Cornish R, John A, Boyd A, *et al.* Defining adolescent common mental disorders using electronic primary care data: A comparison with outcomes measured using the CIS-R. *BMJ Open* 2016;**6**. doi:10.1136/bmjopen-2016-013167

11 John A, McGregor J, Fone D, *et al.* Case-finding for common mental disorders of anxiety and depression in primary care: An external validation of routinely collected data. *BMC Med Inform Decis Mak* 2016;**16**:1–10. doi:10.1186/s12911-016-0274-7

12 Larvin H, Peckham E, Prady SL. Case-finding for common mental disorders in primary care using routinely collected data: a systematic review. *Soc Psychiatry Psychiatr Epidemiol* 2019;**54**:1161–75. doi:10.1007/s00127-019-01744-4

13 Davis KAS, Sudlow CLM, Hotopf M. Can mental health diagnoses in administrative data be used for research? A systematic review of the accuracy of routinely collected diagnoses. *BMC Psychiatry* 2016;**16**:1–11. doi:10.1186/s12888-016-0963-x

14 Coyne JC, Schwenk TL, Fechner-Bates S. Nondetection of depression by primary care physicians reconsidered. *Gen Hosp Psychiatry* 1995;**17**:3–12. doi:10.1016/0163-8343(94)00056-J

15 Cepoiu M, McCusker J, Cole MG, *et al.* Recognition of depression by non-psychiatric physicians - A systematic literature review and meta-analysis. *J Gen Intern Med* 2008;**23**:25–36. doi:10.1007/s11606-007-0428-5

16 Bebbington P, Meltzer H, Brugha T, *et al.* Unequal access and unmet need: Neurotic disorders

For peer review only - http://bmjopen.bmj.com/site/about/guidelines.xhtml

and the use of primary care services. *Psychol Med* 2000;**30**:1359–67. doi:10.1080/095402602100004602 9

17 Rait G, Walters K, Griffin M, *et al.* Recent trends in the incidence of recorded depression in primary care. *Br J Psychiatry* 2009;**195**:520–4. doi:10.1192/bjp.bp.108.058636

18 Wijlaars LPMM, Nazareth I, Petersen I. Trends in depression and antidepressant prescribing in children and adolescents: A cohort study in the health improvement network (THIN). *PLoS One* 2012;**7**. doi:10.1371/journal.pone.0033181

19 Iliffe S, Williams G, Fernandez V, *et al.* General practitioners' understanding of depression in young people: Qualitative study. *Prim Heal Care Res Dev* 2008;**9**:269–79. doi:10.1017/S1463423608000868

20 O'Brien D, Harvey K, Howse J, *et al.* Barriers to managing child and adolescent mental health problems: A systematic review of primary care practitioners' perceptions. *Br J Gen Pract* 2016;**66**:e693–707. doi:10.3399/bjgp16X687061

21 Zwaanswijk M, Verhaak PFM, Bensing JM, *et al.* Help seeking for emotional and behavioural problems in children and adolescents: A review of recent literature. *Eur Child Adolesc Psychiatry* 2003;**12**:153–61. doi:10.1007/s00787-003-0322-6

22 O'Brien D, Harvey K, Creswell C. Barriers to and facilitators of the identification, management and referral of childhood anxiety disorders in primary care: A survey of general practitioners in England. *BMJ Open* 2019;**9**:1–8. doi:10.1136/bmjopen-2018-023876

23 Sayal K. Annotation: Pathways to care for children with mental health problems. *J Child Psychol Psychiatry Allied Discip* 2006;**47**:649–59. doi:10.1111/j.1469-7610.2005.01543.x

24 Henderson JG, Pollard CA, Jacobi KA, *et al.* Help-seeking patterns of community residents with depressive symptoms. *J Affect Disord* 1992;**26**:157–62.

25 Fryers T, Melzer D, Jenkins R. Social inequalities and the common mental disorders - A systematic review of the evidence. *Soc Psychiatry Psychiatr Epidemiol* 2003;**38**:229–37. doi:10.1007/s00127-003-0627-2

26 Adler NE, Stewart J. Health disparities across the lifespan: Meaning, methods, and mechanisms. *Ann N Y Acad Sci* 2010;**1186**:5–23. doi:10.1111/j.1749-6632.2009.05337.x

27 Prady SL, Pickett KE, Petherick ES, *et al.* Evaluation of ethnic disparities in detection of depression and anxiety in primary care during the maternal period: Combined analysis of routine and cohort data. *Br J Psychiatry* 2016;**208**:453–61. doi:10.1192/bjp.bp.114.158832

28 Bebbington P, Brugha T, Meltzer H, *et al.* Neurotic disorders and the receipt of psychiatric treatment. *Psychol Med* 2000;**30**:1369–76. doi:10.1080/095402602100004601 0

29 Prady SL, Pickett KE, Gilbody S, *et al.* Variation and ethnic inequalities in treatment of common mental disorders before, during and after pregnancy: Combined analysis of routine and research data in the Born in Bradford cohort. *BMC Psychiatry* 2016;**16**:1–13. doi:10.1186/s12888-016-0805-x

30 Fernandez A, Salvador-Carulla L, Choi I, *et al.* Development and validation of a prediction algorithm for the onset of common mental disorders in a working population. *Aust N Z J Psychiatry* 2018;**52**:47–58. doi:10.1177/0004867417704506

31 Cornish RP, Macleod J, Boyd A, *et al.* Factors associated with participation over time in the Avon Longitudinal Study of Parents and Children: a study using linked education and primary care data. *Int J Epidemiol* 2020;:1–10. doi:10.1093/ije/dyaa192

For peer review only - http://bmjopen.bmj.com/site/about/guidelines.xhtml

32 Northstone K, Lewcock M, Groom A, *et al.* The Avon Longitudinal Study of Parents and Children (ALSPAC): an update on the enrolled sample of index children in 2019 [version 1; peer review: 2 approved]. *Wellcome Open Res* 2019;**4**:1–10. doi:10.12688/wellcomeopenres.15132.1

33 Fraser A, Macdonald-wallis C, Tilling K, *et al.* Cohort profile: The avon longitudinal study of parents and children: ALSPAC mothers cohort. *Int J Epidemiol* 2013;**42**:97–110. doi:10.1093/ije/dys066

34 Boyd A, Golding J, Macleod J, *et al.* Cohort profile: The ‘Children of the 90s’-The index offspring of the avon longitudinal study of parents and children. *Int J Epidemiol* 2013;**42**:111–27. doi:10.1093/ije/dys064

35 Harris PA, Taylor R, Thielke R, *et al.* Research electronic data capture (REDCap)-A metadata-driven methodology and workflow process for providing translational research informatics support. *J Biomed Inform* 2009;**42**:377–81. doi:10.1016/j.jbi.2008.08.010

36 von Elm E, Altman DG, Egger M, *et al.* The Strengthening the Reporting of Observational Studies in Epidemiology (STROBE) Statement: guidelines for reporting observational studies. doi:10.4324/9781315883595-19

37 Boyd A, Tilling K, Cornish R, *et al.* Professionally designed information materials and telephone reminders improved consent response rates: Evidence from an RCT nested within a cohort study. *J Clin Epidemiol* 2015;**68**:877–87. doi:10.1016/j.jclinepi.2015.03.014

38 Goodman R, Ford T, Richards H, *et al.* The Development and Well-Being Assessment: Description and Initial Validation of an Integrated Assessment of Child and Adolescent Psychopathology. *J Child Psychol Psychiatry* 2000;**41**:645–55. doi:10.1111/j.1469-7610.2000.tb02345.x

39 Lewis G, Pelosi AJ, Araya R, *et al.* Measuring psychiatric disorder in the community: A standardized assessment for use by lay interviewers. *Psychol Med* 1992;**22**:465–86. doi:10.1017/S0033291700030415

40 Brugha TS, Bebbington PE, Jenkins R, *et al.* Cross validation of a general population survey diagnostic interview: A comparison of CIS-R with SCAN ICD-10 diagnostic categories. *Psychol Med* 1999;**29**:1029–42. doi:10.1017/S0033291799008892

41 Angold A, Costello EJ, Messer SC, *et al.* Development of a short questionnaire for use in epidemiological studies of depression in children and adolescents. *Int J Methods Psychiatr Res* 1995;**5**:237–49.

42 Thabrew H, Stasiak K, Bavin LM, *et al.* Validation of the Mood and Feelings Questionnaire (MFQ) and Short Mood and Feelings Questionnaire (SMFQ) in New Zealand help-seeking adolescents. *Int J Methods Psychiatr Res* 2018;**27**:1–9. doi:10.1002/mpr.1610

43 Roseman M, Kloda LA, Saadat N, *et al.* Accuracy of Depression Screening Tools to Detect Major Depression in Children and Adolescents: A Systematic Review. *Can J Psychiatry* 2016;**61**:746–57. doi:10.1177/0706743716651833

44 Turner N, Joinson C, Peters TJ, *et al.* Validity of the Short Mood and feelings questionnaire in late adolescence. *Psychol Assess* 2014;**26**:752–62. doi:10.1037/a0036572

45 Hastie T, Tibshirani R, Wainwright M. *Statistical Learning with Sparsity: The Lasso and Generalizations*. CRC Press 2015.

46 Tibshirani R. Regression Shrinkage and Selection via the Lasso. *J R Stat Soc Ser B Stat*

For peer review only - http://bmjopen.bmj.com/site/about/guidelines.xhtml

Methodol 1996;**58**:267–88.

47 McManus S, Bebbington P, Jenkins R, *et al.* Mental health and wellbeing in England: Adult Psychiatric Morbidity Survey 2014. Leeds: 2016. doi:10.1079/9781786393401.0000

48 Perry BI, Osimo EF, Upthegrove R, *et al.* Development and external validation of the Psychosis Metabolic Risk Calculator (PsyMetRiC): a cardiometabolic risk prediction algorithm for young people with psychosis. *The Lancet Psychiatry* 2021;**8**:589–98. doi:10.1016/S2215-0366(21)00114-0

49 Price SJ, Stapley SA, Shephard E, *et al.* Is omission of free text records a possible source of data loss and bias in Clinical Practice Research Datalink studies? A case-control study. *BMJ Open* 2016;**6**:1–7. doi:10.1136/bmjopen-2016-011664

50 NICE. Depression in children and young people: Identification and management. 2019. doi:10.1211/CP.2018.20204575

51 Children's Commissioner for England. Lightning Review: Access to Child and Adolescent Mental Health Services, May 2016. London, UK: 2016. https://www.childrenscommissioner.gov.uk/publication/lightning-review-access-to-child-and-adolescent-mental-health-services/ (accessed 3/3/2020)

52 Moran P, Jenkins R, Tylee A. The prevalence of personality disorder among U.K. primary care attenders. *Prim Care Companion J Clin Psychiatry* 2000;**2**:148.

53 Gask L, Evans M, Kessler D. Personality disorder. *BMJ* 2013;**347**:1–7. doi:10.1136/bmj.f5276

54 Moran P, Rendu A, Jenkins R, *et al.* The impact of personality disorder in UK primary care: A 1-year follow-up of attenders. *Psychol Med* 2001;**31**:1447–54. doi:10.1017/s003329170105450z

55 Carr MJ, Ashcroft DM, Kontopantelis E, *et al.* Premature death among primary care patients with a history of self-harm. *Ann Fam Med* 2017;**15**:246–54. doi:10.1370/afm.2054

56 Hernán M, Robins J. *Causal Inference: What If*. Boca Raton: : Chapman & Hall/CRC Press 2020.

57 Munafò MR, Tilling K, Taylor AE, *et al.* Collider scope: When selection bias can substantially influence observed associations. *Int J Epidemiol* 2018;**47**:226–35. doi:10.1093/ije/dyx206

58 Ellis DA, McQueenie R, McConnachie A, *et al.* Demographic and practice factors predicting repeated non-attendance in primary care: a national retrospective cohort analysis. *Lancet Public Heal* 2017;**2**:e551–9. doi:10.1016/S2468-2667(17)30217-7

59 Leach LS, Christensen H, Mackinnon AJ, *et al.* Gender differences in depression and anxiety across the adult lifespan: The role of psychosocial mediators. *Soc Psychiatry Psychiatr Epidemiol* 2008;**43**:983–98. doi:10.1007/s00127-008-0388-z

60 McLean CP, Asnaani A, Litz BT, *et al.* Gender differences in anxiety disorders: Prevalence, course of illness, comorbidity and burden of illness. *J Psychiatr Res* 2011;**45**:1027–35. doi:10.1016/j.jpsychires.2011.03.006

61 Ford E, Carroll JA, Smith HE, *et al.* Extracting information from the text of electronic medical records to improve case detection: A systematic review. *J Am Med Informatics Assoc* 2016;**23**:1007–15. doi:10.1093/jamia/ocv180

62 Zuckerbrot RA, Cheung A, Jensen PS, *et al.* Guidelines for adolescent depression in primary care (GLAD-PC): Part I. Practice preparation, identification, assessment, and initial management. *Pediatrics* 2018;**141**. doi:10.1542/peds.2017-4081

For peer review only - http://bmjopen.bmj.com/site/about/guidelines.xhtml

Figure 1: Comparing primary care common mental disorder (CMD) and depression rates in participants with vs without ALSPAC data at each time point. For participants who did not attend the clinic/complete the questionnaire, the age to define a 'current' diagnosis was based on +/- 6 months from the average age each clinic/questionnaire was completed. Individuals who have primary care data and attended/completed the clinic/questionnaire, but do not have ALSPAC-derived depression/CMD data (as this session was not completed for whatever reason), are not included in the figure below. Full details of these numbers, and the data for this figure, are provided in table S6.

Figure 2: Sensitivity, specificity and positive/negative predicted values for depression (black) and common mental disorders (CMDs; red) over each of the time points studies. Results are based on the definition 'current diagnosis, symptoms or treatment' to determine cases in primary care records, treating the ALSPAC data as the reference standard. Note that CMDs were only assessed at the age 15/16 and 17/18 clinics.

For peer review only

For peer review only - http://bmjopen.bmj.com/site/about/guidelines.xhtml

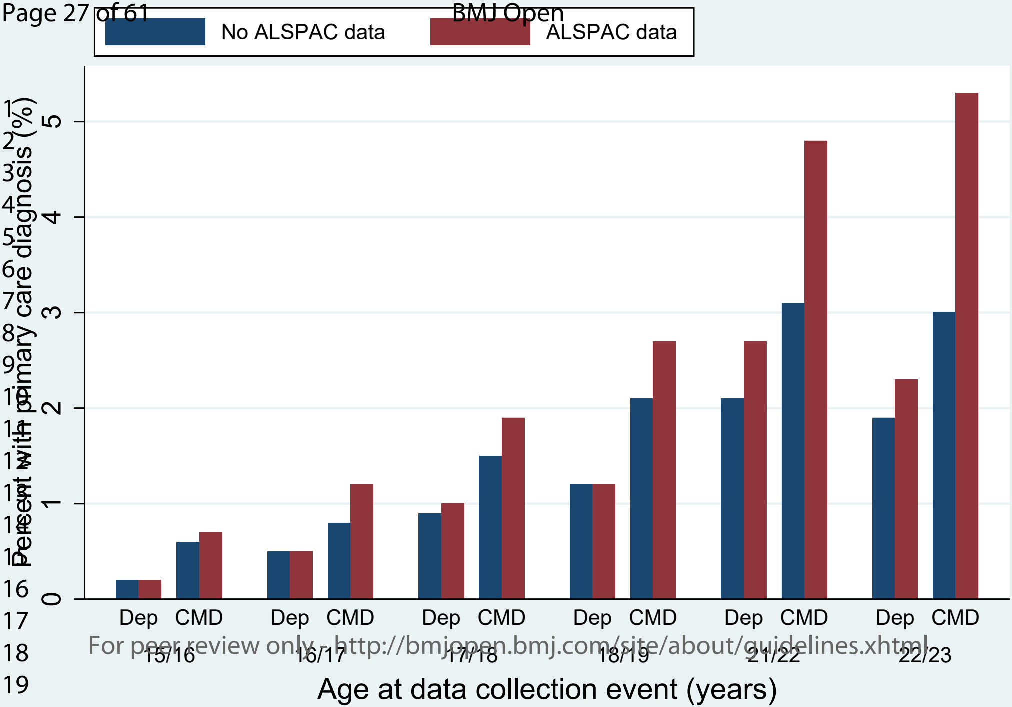

For peer review only - http://bmjopen.bmj.com/site/about/guidelines.xhtml

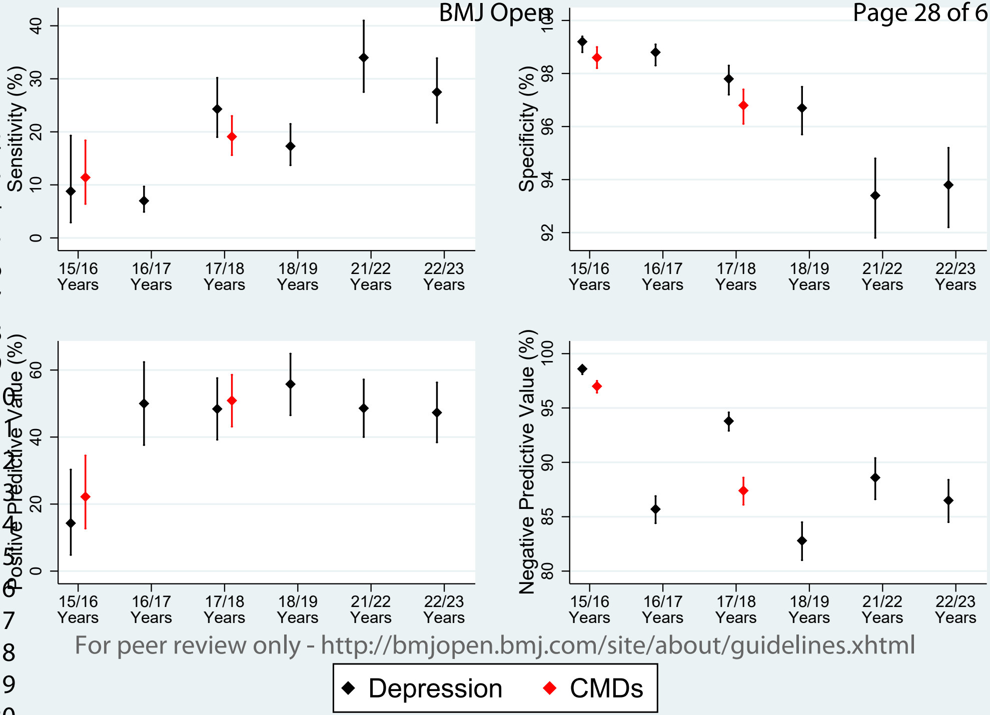

For peer review only - http://bmjopen.bmj.com/site/about/guidelines.xhtml

Supplementary information for 'Assessing and predicting adolescent and early adulthood common mental disorders using electronic primary care data: analysis of a prospective cohort study (ALSPAC) in southwest England'

Linkage to primary care data

The Secure Anonymised Information Linkage (SAIL) project has established a system which allows individual-level data from multiple sources – including electronic primary care data – to be linked together and analysed securely [1]. In collaboration with SAIL and GP software system providers, the Project to Enhance ALSPAC through Record Linkage (PEARL) developed two methods to extract primary care data. The first was a pilot extraction in 2012 using a sample of study participants based on an 'opt-in' consent principle, from which 2,806 participants consented to having their primary care records accessed (see [2] for further details). The second method was the main data extraction, where all ALSPAC participants with known addresses were sent fair processing materials and allowed to 'opt-out' of having their primary care records extracted, if they did not consent to this. Extraction of the electronic primary care data occurred in partnership with EMIS, which provides the software systems to most of the practices in the Bristol area. Records were extracted from all EMIS practices in the Bristol, North Somerset and South Gloucestershire clinical commissioning group which had consented to the NHS South West Commissioning Support Unit (SWCSU) governance framework and data extraction mechanism. Approval for this ALSPAC project was obtained from the SWCSU Security and Informatics Group and the SWCSU informed all participating practices and offered the option to opt-out. For more details, see [3].

Data processing methods after this extraction were identical for both the pilot and main extraction phases. The extracted electronic primary care records were pseudonymised and then securely transferred to a SAIL secure setting (a UK Secure eResearch Platform; UKSeRP) using SAIL's 'split file' method. NHS standards of encryption and security were maintained throughout. These protocols have been described in more detail in previous publications [2,3].

References cited in supplementary information:

1 Ford D V., Jones KH, Verplancke JP, *et al.* The SAIL Databank: Building a national architecture for e-health research and evaluation. *BMC Health Serv Res* 2009;**9**:1–12. doi:10.1186/1472-6963-9-157

2 Cornish R, John A, Boyd A, *et al.* Defining adolescent common mental disorders using electronic primary care data: A comparison with outcomes measured using the CIS-R. *BMJ Open* 2016;**6**. doi:10.1136/bmjopen-2016-013167

3 Cornish RP, Macleod J, Boyd A, *et al.* Factors associated with participation over time in the Avon Longitudinal Study of Parents and Children: a study using linked education and primary care data. *Int J Epidemiol* 2020;:1–10. doi:10.1093/ije/dyaa192

For peer review only

Table S1: Read code lists

Condition	Reference for Read code list (doi) / list of codes
**Anxiety – diagnosis, symptoms, treatment**	Cornish *et al*., 2016: http://dx.doi.org/10.1136/bmjopen-2016-013167
**Depression – diagnosis, symptoms, treatment**	Cornish *et al*., 2016: http://dx.doi.org/10.1136/bmjopen-2016-013167
**ADHD**	E2E.. (Childhood hyperkinetic syndrome)<br>E2E0. (Child attention deficit disorder)<br>E2E00 (Attention deficit without hyperactivity)<br>E2E01 (Attention deficit without hyperactivity)<br>E2E0z (Child attention deficit disorder NOS)<br>E2E1. (Hyperkinesis with developmental delay)<br>E2E2. (Hyperkinetic conduct disorder)<br>E2Ey. (Other hyperkinetic manifestation)<br>E2Ez. (Hyperkinetic syndrome NOS)<br>Eu90. ([X]Hyperkinetic disorders)<br>Eu900 ([X]Attention deficit hyperactivity disorder)<br>Eu901 ([X]Hyperkinetic conduct disorder)<br>Eu902 ([X]Deficits in attention motor control and perception)<br>Eu90y ([X]Other hyperkinetic disorders)<br>Eu90z ([X]Hyperkinetic disorder unspecified)<br>Eu9y7 ([X]Attention deficit disorder)<br>9Ngp. (On drug therapy ADHD)<br>8BPT. (Drug therapy for ADHD)
**Asthma**	Cornish *et al*., 2014: http://dx.doi.org/10.1136/bmjopen-2014-005345
**Autism Spectrum Disorder (ASD)**	Hagberg & Jick, 2017: https://doi.org/10.2147/CLEP.S139107
**Bipolar disorder: diagnosis**	Carr *et al*., 2017: https://dx.doi.org/10.1370%2Fafm.2054 (used diagnosis codes only from their list)
**Conduct disorder**	E2C4. (Mixed disturbance of conduct and emotion)<br>E2C4z (Mixed disturbance of conduct and emotion NOS)<br>Eu912 ([X]Socialized conduct disorder)<br>Eu911 ([X]Conduct disorder, solitary aggressive type)<br>E2C2z (Socialised conduct disorder NOS)<br>E2C1. (Nonaggressive unsocial conduct disorder)<br>Eu911 ([X]Unsocialized conduct disorder)<br>E2C.. (Disturbance of conduct NEC)<br>Eu91. ([X]Conduct disorders)<br>Eu92z ([X]Mixed disorder of conduct and emotions, unspecified)<br>Eu92. ([X]Mixed disorders of conduct and emotions)<br>E2C2. (Socialised conduct disorder)<br>E2C0. (Aggressive unsocial conduct disorder)<br>E2C1z (Nonaggressive unsocial conduct disorder NOS)<br>E2C0z (Aggressive unsocial conduct disorder NOS)<br>Eu901 ([X]Hyperkinetic disorder associated with conduct disorder)<br>E2Czz (Disturbance of conduct NOS)<br>Eu91z ([X]Childhood conduct disorder NOS)<br>Eu92y ([X]Conduct disorder associated with emotional disorder)<br>Eu91y ([X]Other conduct disorders)<br>E2Cy. (Other conduct disturbances)<br>E2Cz. (Unspecified disturbance of conduct)

	E2Cyz (Other conduct disturbances NOS)<br>Eu910 ([X]Conduct disorder confined to the family context)<br>Eu91z ([X]Conduct disorder, unspecified)<br>Eu920 ([X]Depressive conduct disorder)<br>Eu92y ([X]Other mixed disorders of conduct and emotions)<br>Eu913 ([X]Oppositional defiant disorder)<br>E2Dy0 (Childhood and adolescent oppositional disorder)
**Eating disorder**	Carr *et al*., 2017: https://dx.doi.org/10.1370%2Fafm.2054
**Eczema**	Mukherjee *et al*., 2014: https://dx.doi.org/10.1136/bmjopen-2014-006647 (used diagnosis codes only from their list)
**Family history depression**	Read codes 1285.
**Family history mental illness**	128% except 1281. (Read codes starting 128 - % used as sort of wild card for Read codes. 1281. is family history senile dementia)
**General symptoms**	1954. (Indigestion)<br>1968. (Abdominal discomfort)<br>1969. (Abdominal pain)<br>1981. (c/o Nausea)<br>1982. (Nausea present)<br>198Z. (Nausea NOS)<br>1B14. (Tenseness)<br>1B18. (Inadequate)<br>1B1G. (Headache)<br>1B1G1 (c/o Headache)<br>1B1N. (Poor self-esteem)<br>1B1O. (Restless)<br>1B51 (Dizziness symptoms)<br>E2781 (Tension headache)<br>R0040 ([D] Dizziness)<br>R00Z2 ([D] Pain, generalised)<br>R040. ([D] Headache)<br>R0700 ([D] Nausea)<br>R090. ([D] Abdominal pain)<br>R090z ([D] Abdominal pain NOS)<br>R090E ([D] Recurrent abdominal pain)<br>R090N ([D] Non-specific abdominal pain)
**History of depression**	1465. (H/O: depression)
**History of mental illness**	146%. (H/O: psychiatric disorder)
**Number of consultations and prescriptions**	Cornish *et al*., 2020: https://doi.org/10.1093/ije/dyaa192
**Psychosis**	Carr *et al*., 2017: https://dx.doi.org/10.1370%2Fafm.2054 (used diagnosis codes only from their list)
**Referral to mental health services**	8H49. (Psychiatric referral)<br>8H4P. (Referral to child psychiatrist)<br>8H78. (Referral to counsellor)<br>8H7A. (Referral to mental health worker)<br>8H7B. (Referral to community psychiatric nurse)<br>8H7T. (Referral to psychotherapist)<br>8H7W. (Referral to TOP counselling)<br>8Hc.. (Referral to mental health team)<br>8Hc0. (Referral to community mental health team)<br>8Hc1. (Referral to mental health crisis team)

For peer review only

	8Hc2. (Referral to primary care mental health team)<br>8HHn. (Referral to non-NHS mental health community service)<br>8HHp. (Referral for guided self-help for anxiety)<br>8HHq. (Referral for guided self-help for depression)<br>8HHr. (Referral for mental health self-help literature)<br>8HHu. (Referral to primary care mental health gateway worker)<br>8HHR. (Referral to child and adolescent psychiatry service)<br>8HHT. (Refer to psychotherapist)<br>8HkK. (Referral to improving access to psychological therapies programme)<br>8HVO. (Private referral to psychiatrist)
**Schizophrenia**	Carr *et al*., 2017: https://dx.doi.org/10.1370%2Fafm.2054 (used diagnosis codes only from their list)
**Self-harm**	Thomas *et al*., 2012 : https://dx.doi.org/10.1111%2Fbcp.12059
**Smoking**	Cornish *et al*., 2020: https://doi.org/10.1093/ije/dyaa192
**Somatic symptoms**	16122 (Loss of appetite – symptom)<br>1613. (Increased appetite)<br>1615. (Reduced appetite)<br>168% (Tired all the time, fatigue, tiredness, tearful, malaise/lethargy)<br>1B15. (Irritable)<br>1B16. (Agitated)<br>1B19. (Suicidal)<br>1B1B. (Cannot sleep – insomnia)<br>1B1D. (Nightmares)<br>1B1I. (Crying, excessive)<br>1B1I1 (C/O tearfulness)<br>1BD1. (Suicidal ideation)<br>1BD2. (Morbid thoughts)<br>1BD3. (Suicidal plans)<br>1BD4. (Suicide risk)<br>1BD5. (High suicide risk)<br>1BD6. (Moderate suicide risk)<br>1BD8. (At risk of deliberate self harm)<br>1BDA. (Thoughts of deliberate self harm)<br>1BJ.. (Loss of confidence)<br>1BR.. (Reduced concentration)<br>1BW.. (Poor concentration)<br>E2052 (Tired all the time)<br>E2741 (Insomnia NOS)
**Substance abuse (alc/drugs)**	Eu1% ([X] Mental and behavioural disorders due to psychoactive substance abuse)<br>E23% (Alcohol dependence)<br>E24% (Drug dependence)

Table S2: Details of variables used in the misclassification analyses.

Variable	Origin	Type	Notes
**Sex**	ALSPAC	Binary	0 = Male; 1 = Female
**Age at data collection event**	ALSPAC	Cont.	Measured in months
**Mother's age at birth**	ALSPAC	Cont.	Measured in years
**Mother's home ownership status**	ALSPAC	Binary	0 = Owned/Mortgaged; 1 = Rented/Housing Association/Council House/Other
**Mother's marital status**	ALSPAC	Binary	0 = Married; 1 = Not married
**Mother's age at first pregnancy**	ALSPAC	Cont.	Measured in years
**Mother's parity at child's birth**	ALSPAC	Binary	0 = First child; 1 = Second or later child
**Mother's highest education level**	ALSPAC	Binary	0 = CSE/Vocational/O Level; 1 = A Level/Degree
**Father's highest education level**	ALSPAC	Binary	0 = CSE/Vocational/O Level; 1 = A Level/Degree<br>Assumes Mother's partner is child's Father.
**Mother's social class**	ALSPAC	Binary	0 = I/II – Professional/Managerial; 1 – III/IV/V – Skilled/Partly skilled/Unskilled
**Father's social class**	ALSPAC	Binary	0 = I/II – Professional/Managerial; 1 – III/IV/V – Skilled/Partly skilled/Unskilled<br>Assumes Mother's partner is child's Father.
**Child's ethnic background**	ALSPAC	Binary	1 = White; 2 = Non-white
**Child's education (# A*-C GCSE equivalents)**	ALSPAC	Cont.	From education linkage data
**CIS-R depression score**	ALSPAC	Cat.	Specific to TF4 analyses
**CIS-R diagnosis of moderate depressive episode**	ALSPAC	Binary	Specific to TF4 analysis
**CIS-R diagnosis of severe depressive episode**	ALSPAC	Binary	Specific to TF4 analysis
**CIS-R Generalised Anxiety Disorder (GAD) symptoms**	ALSPAC	Binary	Specific to TF4 analysis
**CIS-R total score**	ALSPAC	Cont.	Specific to TF4 analysis
**DAWBA predicted depression**	ALSPAC	Cat.	Specific to TF3 analyses
**DAWBA predicted anxiety**	ALSPAC	Cat.	Specific to TF3 analyses
**Total MFQ score**	ALSPAC	Cont.	Specific to CCS, CCT, YPA and YPB analyses
**Historical depression diagnosis**	Primary care	Binary	0 = No; 1 = Yes
**Historical anxiety diagnosis**	Primary care	Binary	0 = No; 1 = Yes
**Current anxiety diagnosis**	Primary care	Binary	0 = No; 1 = Yes
**Historical phobia diagnosis**	Primary care	Binary	0 = No; 1 = Yes
**Current phobia diagnosis**	Primary care	Binary	0 = No; 1 = Yes
**Historical depression symptoms**	Primary care	Binary	0 = No; 1 = Yes
**Current depression symptoms**	Primary care	Binary	0 = No; 1 = Yes
**Historical anxiety symptoms**	Primary care	Binary	0 = No; 1 = Yes
**Current anxiety symptoms**	Primary care	Binary	0 = No; 1 = Yes

For peer review only

**Historical somatic symptoms**	Primary care	Binary	0 = No; 1 = Yes
**Current somatic symptoms**	Primary care	Binary	0 = No; 1 = Yes
**Historical general symptoms**	Primary care	Binary	0 = No; 1 = Yes
**Current general symptoms**	Primary care	Binary	0 = No; 1 = Yes
**Historical anti-depressant treatment**	Primary care	Binary	0 = No; 1 = Yes
**Current anti-depressant treatment**	Primary care	Binary	0 = No; 1 = Yes
**Historical anti-anxiety treatment**	Primary care	Binary	0 = No; 1 = Yes
**Current anti-anxiety treatment**	Primary care	Binary	0 = No; 1 = Yes
**# of GP consultations per year**	Primary care	Cont.	Average over 2-year period at time of data collection
**# of GP prescriptions per year**	Primary care	Cont.	Average over 2-year period at time of data collection
**Asthma diagnosis**	Primary care	Binary	0 = No; 1 = Yes (Includes any previous read codes, up to 6 months after age at event)
**Eczema diagnosis**	Primary care	Binary	0 = No; 1 = Yes (Includes any previous read codes, up to 6 months after age at event)
**Eating disorder**	Primary care	Binary	0 = No; 1 = Yes (Includes any previous read codes, up to 6 months after age at event)
**ADHD**	Primary care	Binary	0 = No; 1 = Yes (Includes any previous read codes, up to 6 months after age at event)
**Conduct disorder**	Primary care	Binary	0 = No; 1 = Yes (Includes any previous read codes, up to 6 months after age at event)
**Autism spectrum disorder**	Primary care	Binary	0 = No; 1 = Yes (Includes any previous read codes, up to 6 months after age at event)
**Substance abuse (alcohol/drugs)**	Primary care	Binary	0 = No; 1 = Yes (Includes any previous read codes, up to 6 months after age at event)
**Family history of mental illness**	Primary care	Binary	0 = No; 1 = Yes (Includes any previous read codes, up to 6 months after age at event)
**Family history of depression**	Primary care	Binary	0 = No; 1 = Yes (Includes any previous read codes, up to 6 months after age at event)
**History of mental health issues**	Primary care	Binary	0 = No; 1 = Yes (Includes any previous read codes, up to 6 months after age at event)
**History of depression**	Primary care	Binary	0 = No; 1 = Yes (Includes any previous read codes, up to 6 months after age at event)
**Self-harm**	Primary care	Binary	0 = No; 1 = Yes (Includes any previous read codes, up to 6 months after age at event)
**Other psychological illness (schizophrenia, bipolar disorder and psychosis)**	Primary care	Binary	0 = No; 1 = Yes (Includes any previous read codes, up to 6 months after age at event)
**Any other mental health issues**	Primary care	Binary	0 = No; 1 = Yes (Combining eating disorder, ADHD, conduct disorder, ASD, substance abuse and other psychological illness, as low cell counts for these individual variables)
**Referral to mental health services**	Primary care	Binary	0 = No; 1 = Yes (Includes any previous read codes, up to 6 months after age at event)
**Smoking (or ex-smoker)**	Primary care	Binary	0 = No; 1 = Yes (Includes any previous read codes, up to 6 months after age at event)

Table S3: Details of variables used in the lasso analyses.

Variable	Type	Notes
**Sex**	Binary	0 = Male; 1 = Female
**Age at ALSPAC data collection**	Cont.	Measured in months
**Historical depression diagnosis**	Binary	0 = No; 1 = Yes
**Historical anxiety diagnosis**	Binary	0 = No; 1 = Yes
**Current anxiety diagnosis**	Binary	0 = No; 1 = Yes
**Historical phobia diagnosis**	Binary	0 = No; 1 = Yes
**Current phobia diagnosis**	Binary	0 = No; 1 = Yes
**Historical depression symptoms**	Binary	0 = No; 1 = Yes
**Current depression symptoms**	Binary	0 = No; 1 = Yes
**Historical anxiety symptoms**	Binary	0 = No; 1 = Yes
**Current anxiety symptoms**	Binary	0 = No; 1 = Yes
**Historical somatic symptoms**	Binary	0 = No; 1 = Yes
**Current somatic symptoms**	Binary	0 = No; 1 = Yes
**Historical general symptoms**	Binary	0 = No; 1 = Yes
**Current general symptoms**	Binary	0 = No; 1 = Yes
**Historical anti-depressant treatment**	Binary	0 = No; 1 = Yes
**Current anti-depressant treatment**	Binary	0 = No; 1 = Yes
**Historical anti-anxiety treatment**	Binary	0 = No; 1 = Yes
**Current anti-anxiety treatment**	Binary	0 = No; 1 = Yes
**# of GP consultations per year**	Cont.	Average over 2-year period at time of data collection
**# of GP prescriptions per year**	Cont.	Average over 2-year period at time of data collection
**Asthma diagnosis**	Binary	0 = No; 1 = Yes (Includes any previous read codes, up to 6 months after age at event)
**Eczema diagnosis**	Binary	0 = No; 1 = Yes (Includes any previous read codes, up to 6 months after age at event)
**Eating disorder**	Binary	0 = No; 1 = Yes (Includes any previous read codes, up to 6 months after age at event)
**ADHD**	Binary	0 = No; 1 = Yes (Includes any previous read codes, up to 6 months after age at event)
**Conduct disorder**	Binary	0 = No; 1 = Yes (Includes any previous read codes, up to 6 months after age at event)
**Autism spectrum disorder**	Binary	0 = No; 1 = Yes (Includes any previous read codes, up to 6 months after age at event)
**Substance abuse (alcohol/drugs)**	Binary	0 = No; 1 = Yes (Includes any previous read codes, up to 6 months after age at event)
**Family history of mental illness**	Binary	0 = No; 1 = Yes (Includes any previous read codes, up to 6 months after age at event)
**Family history of depression**	Binary	0 = No; 1 = Yes (Includes any previous read codes, up to 6 months after age at event)

For peer review only

**History of mental health issues**	Binary	0 = No; 1 = Yes (Includes any previous read codes, up to 6 months after age at event)
**History of depression**	Binary	0 = No; 1 = Yes (Includes any previous read codes, up to 6 months after age at event)
**Self-harm**	Binary	0 = No; 1 = Yes (Includes any previous read codes, up to 6 months after age at event)
**Other psychological illness (schizophrenia, bipolar disorder and psychosis)**	Binary	0 = No; 1 = Yes (Includes any previous read codes, up to 6 months after age at event)
**Any other mental health issues**	Binary	0 = No; 1 = Yes (Combining eating disorder, ADHD, conduct disorder, ASD, substance abuse and other psychological illness, as low cell counts for these individual variables)
**Referral to mental health services**	Binary	0 = No; 1 = Yes (Includes any previous read codes, up to 6 months after age at event)
**Smoking (or ex-smoker)**	Binary	0 = No; 1 = Yes (Includes any previous read codes, up to 6 months after age at event)

For peer review only

Table S4: Reasons for having ALSPAC clinic/questionnaire common mental disorder (CMD) data, but not primary care linkage data. Note also that the numbers excluded do not always correspond to the numbers with ALSPAC, but not primary care, data; this is because some individuals may have been counted both in the 'lost from records' and 'first entered records' columns (i.e., they first have primary care data within 18 months of the time-point, then were lost from primary care records again within 6 months after the time-point), or have missing data. Numbers in brackets denote the percentage of individuals in each exclusion category of the total participants without primary care data but with ALSPAC data. Most individuals are likely to be lost from primary care records because they moved out of the Bristol area, but it is also possible that they stayed within the Bristol area but moved to a GP practice using a different software system from which it was not possible to extract primary care data. Similarly, individuals may first appear in the primary care data after the time-point because they either moved into the Bristol area at this time, or stayed in the Bristol area but moved to a GP practice from which it was possible to extract primary care data.

**Age (time point)**	**# with ALSPAC CMD data**	**# with ALSPAC, but not primary care, CMD data**	**# Excluded as no primary care data (no consent or no records)**	**# Excluded as lost from primary care records before time-point**	**# Excluded as first primary care data is after time-point**
**Age 15/16 (TF3 clinic)**	5,332	1,669	737 (44.2%)	614 (36.8%)	313 (18.8%)
**Age 16/17 (CCS quest)**	4,950	1,737	762 (43.9%)	690 (39.7%)	283 (16.3%)
**Age 17/18 (TF4 clinic)**	4,534	1,450	583 (40.2%)	616 (42.5%)	254 (17.5%)
**Age 18/19 (CCT quest)**	3,302	1,320	510 (38.6%)	601 (45.5%)	214 (16.2%)
**Age 21/22 (YPA quest)**	3,283	1,985	471 (23.7%)	1,462 (73.7%)	64 (3.2%)
**Age 22/23 (YPB quest)**	3,896	2,571	563 (21.9%)	1,969 (76.6%)	43 (1.7%)

For peer review only

Table S5: Demographics at age 16/17 CCS questionnaire, 17/18 TF4 clinic, 18/19 CCT questionnaire and 21/22 YPA questionnaire time points. Of those with ALSPAC data, the table compares those who have primary data against those who do not. For categorical variables cells are counts and percentages. For continuous variables cells are means and standard deviations. Note also that the denominators vary as the variables come from different data sources, with different levels of completeness. See table 3 for age 15/16 TF3 and 22/23 YPB questionnaire time points.

	**Age 16/17 (CCS questionnaire) – SMFQ (Depression)**		**Age 17/18 (TF4 clinic) – CIS-R (CMDs and Depression)**		**Age 18/19 (CCT questionnaire) – SMFQ (Depression)**		**Age 21/22 (YPA questionnaire) – SMFQ (Depression)**	
	**Primary care data (*n*=3213)**	**No primary care data (*n*=1737)**	**Primary care data (*n*=3084)**	**No primary care data (*n*=1450)**	**Primary care data (*n*=1982)**	**No primary care data (*n*=1320)**	**Primary care data (*n*=1228)**	**No primary care data (*n*=1985)**
**Sex**								
**Male**	1302 (40.5%)	706 (40.6%)	1358 (44.0%)	624 (43.0%)	703 (35.5%)	470 (35.6%)	456 (35.1%)	710 (35.8%)
**Female**	1911 (59.5%)	1031 (59.4%)	1726 (56.0%)	826 (57.0%)	1279 (64.5%)	850 (64.4%)	842 (64.9%)	1275 (64.2%)
**Maternal age at child's birth**	29.4 (4.6)	29.4 (4.5)	29.3 (4.6)	29.2 (4.6)	29.4 (4.6)	29.8 (4.6)	29.2 (4.6)	29.8 (4.4)
**Mother's home ownership status**								
**Owned/ Mortgaged**	2549 (85.8%)	1359 (83.6%)	2405 (85.4%)	1115 (83.7%)	1567 (85.2%)	1052 (85.1%)	1015 (85.0%)	1632 (87.6%)
**Rented**	138 (4.6%)	104 (6.4%)	113 (4.0%)	69 (5.2%)	83 (4.5%)	70 (5.7%)	53 (4.4%)	94 (5.0%)
**Council/Housing Association**	220 (7.4%)	101 (6.2%)	229 (8.1%)	101 (7.6%)	148 (8.1%)	72 (5.8%)	98 (8.2%)	89 (4.8%)
**Other**	65 (2.2%)	62 (3.8%)	69 (2.5%)	48 (3.6%)	41 (2.2%)	42 (3.4%)	28 (2.4%)	49 (2.6%)
**Mother's marital status**								
**Never married**	380 (12.7%)	214 (13.0%)	386 (13.6%)	190 (14.1%)	242 (13.0%)	130 (10.4%)	146 (12.1%)	206 (11.0%)
**Single/Divorced**	132 (4.4%)	86 (5.2%)	122 (4.3%)	70 (5.2%)	78 (4.2%)	56 (4.5%)	48 (4.0%)	80 (4.3%)
**First marriage**	2305 (76.9%)	1225 (74.6%)	2165 (76.3%)	998 (74.0%)	1435 (77.0%)	979 (78.2%)	924 (76.6%)	1470 (78.4%)
**2nd/3rd marriage**	182 (6.1%)	118 (7.2%)	164 (5.8%)	91 (6.8%)	108 (5.8%)	87 (7.0%)	88 (7.3%)	120 (6.4%)
**Mother's parity**								
**0**	1410 (47.3%)	840 (51.5%)	1326 (47.2%)	670 (50.6%)	876 (47.4%)	629 (50.8%)	556 (46.5%)	933 (50.2%)
**1**	1058 (35.5%)	553 (33.9%)	999 (35.6%)	444 (33.6%)	666 (36.0%)	413 (33.4%)	433 (36.2%)	631 (34.0%)
**2 or more**	511 (17.5%)	238 (14.6%)	482 (17.2%)	209 (15.8%)	307 (16.6%)	196 (15.8%)	207 (17.3%)	294 (15.8%)
**Mother's highest education level**								
**O level/lower**	1612 (54.1%)	777 (47.7%)	1541 (55.2%)	642 (48.2%)	993 (53.9%)	542 (43.6%)	726 (60.8%)	826 (44.3%)
**A level**	825 (27.7%)	468 (28.7%)	756 (27.1%)	400 (30.0%)	496 (26.9%)	367 (29.6%)	300 (25.1%)	555 (29.7%)
**Degree**	544 (18.3%)	385 (23.6%)	495 (17.7%)	291 (21.8%)	355 (19.3%)	333 (26.8%)	168 (14.1%)	485 (26.0%)

**Father's highest education level**								
**O level/lower**	1217 (44.4%)	556 (36.6%)	1143 (44.5%)	502 (40.7%)	737 (43.7%)	391 (33.2%)	538 (49.3%)	604 (34.2%)
**A level**	828 (30.2%)	465 (30.6%)	795 (30.9%)	363 (29.4%)	510 (30.3%)	365 (31.0%)	344 (31.5%)	528 (29.9%)
**Degree**	699 (25.5%)	498 (32.8%)	632 (24.6%)	368 (29.9%)	439 (26.0%)	423 (35.9%)	209 (19.2%)	634 (35.9%)
**Child ethnicity**								
**White**	2836 (96.4%)	1535 (95.6%)	2639 (95.8%)	1255 (95.4%)	1752 (96.0%)	1186 (96.3%)	1138 (96.2%)	1769 (95.9%)
**Non-white**	105 (3.6%)	70 (4.4%)	116 (4.2%)	60 (4.6%)	74 (4.0%)	46 (3.7%)	45 (3.8%)	75 (4.1%)
**# GCSEs (or equivalents)**	7.7 (3.4)	7.8 (3.4)	7.5 (3.5)	7.6 (3.4)	7.8 (3.3)	8.4 (3.1)	7.5 (3.5)	8.5 (3.0)
**ALSPAC depression diagnosis**								
**No**	2728 (84.9%)	1452 (83.6%)	2841 (92.1%)	1334 (92.0%)	1595 (80.5%)	1095 (83.0%)	1098 (84.6%)	1685 (84.9%)
**Yes**	485 (15.1%)	285 (16.4%)	243 (7.9%)	116 (8.0%)	387 (19.5%)	225 (17.1%)	200 (15.4%)	300 (15.1%)
**ALSPAC common mental disorder (CMD) diagnosis**								
**No**	-	-	2629 (85.3%)	1231 (84.9%)	-	-	-	-
**Yes**	-	-	455 (14.8%)	219 (15.1%)	-	-	-	-

SMFQ: Short Mood and Feelings Questionnaire; *CMDs:* Common mental disorders; *CIS-R:* Clinical Interview Schedule – Revised.

For peer review only

Table S6: Comparing ALSPAC participants who possess primary care data at each time-point, split by whether they attended/completed each specific data collection event. The numbers with and without ALSPAC data are provided, as are differences in rates of current depression or common mental disorder (CMD) diagnoses from the primary care records. For participants who did not attend the clinic/complete the questionnaire, the age to define a 'current' diagnosis was based on +/- 6 months from the average age each clinic/questionnaire was completed. Individuals who have GP data and completed the clinic/questionnaire, but do not have ALSPAC-derived depression/CMD data (as this session was not completed for whatever reason), are not included in the table below. The number of these individuals at each time point are: 104 at the age 15/16 TF3 clinic (2.8% of those with both ALSPAC and primary care data); 82 at the age 16/17 CCS questionnaire (2.5% of those with both ALSPAC and primary care data); 437 at the age 17/18 TF4 clinic (12.4% of those with both ALSPAC and primary care data); 20 at the age 18/19 CCT questionnaire (1% of those with both ALSPAC and primary care data); 63 at the age 21/22 YPA questionnaire (4.6% of those with both ALSPAC and primary care data); 43 at the age 22/23 YPB questionnaire (3.1% of those with both ALSPAC and primary care data). For a graphical summary of these results, see figure 1.

**Age (time point)**	**Ave. age at clinic/quest (years (months))**	**ALSPAC measure completed**	**# with and without ALSPAC CMD/ depression data (%)**	**# with current depression diagnosis in GP records (%)**	**# with current CMD diagnosis in GP records (%)**
**Age 15/16 (TF3 clinic)**	15.5 years (186 mths)	Yes	3,663 (42.8%)	7 (0.2%)	26 (0.7%)
		No	4,902 (57.2%)	8 (0.2%)	29 (0.6%)
**Age 16/17 (CCS quest)**	16.67 years (200 mths)	Yes	3,213 (37.6%)	17 (0.5%)	37 (1.2%)
		No	5,342 (62.4%)	25 (0.5%)	43 (0.8%)
**Age 17/18 (TF4 clinic)**	17.83 years (214 mths)	Yes	3,084 (38.3%)	30 (1.0%)	57 (1.9%)
		No	4,961 (61.7%)	45 (0.9%)	76 (1.5%)
**Age 18/19 (CCT quest)**	18.67 years (224 mths)	Yes	1,982 (23.6%)	24 (1.2%)	53 (2.7%)
		No	6,402 (76.4%)	78 (1.2%)	131 (2.1%)
**Age 21/22 (YPA quest)**	21.92 years (263 mths)	Yes	1,298 (20.4%)	35 (2.7%)	62 (4.8%)
		No	5,055 (79.6%)	106 (2.1%)	155 (3.1%)
**Age 22/23 (YPB quest)**	22/92 years (275 mths)	Yes	1,325 (23.4%)	30 (2.3%)	70 (5.3%)
		no	4,335 (76.6%)	81 (1.9%)	128 (3.0%)

For peer review only

Table S7: Raw data comparing depression and common mental disorder (CMD) diagnoses based on the Development and Well-Being Assessment (DAWBA) data from the age 15/16 TF3 clinic against various definitions derived from the primary care data at this age (*n*=3,663). Note that values with an asterisk have been suppressed for disclosure control purposes as at least one cell has a value < 5. This table also includes sensitivities, specificities, positive predictive values (PPV) and negative predictive values (NPV) for the depression and CMD diagnoses based on the DAWBA data from this clinic. In these analyses we are treating the ALSPAC data as the reference standard.

		**DAWBA Depression**			**DAWBA CMD**		
**Primary care definition**		**No**	**Yes**	**Total**	**No**	**Yes**	**Total**
**Current diagnosis**	No	*	*	*	3,521	116	3,637
	Yes	*	*	*	19	7	26
**Sensitivity**		*			5.7% (2.3; 11.4)		
**Specificity**		*			99.5% (99.2; 99.7)		
**Positive Predictive Value**		*			26.9% (11.6; 47.8)		
**Negative Predictive Value**		*			96.8% (96.2; 97.4)		
		**No**	**Yes**	**Total**	**No**	**Yes**	**Total**
**Current diagnosis, treated**	No	*	*	*	*	*	*
	Yes	*	*	*	*	*	*
**Sensitivity**		*			*		
**Specificity**		*			*		
**Positive Predictive Value**		*			*		
**Negative Predictive Value**		*			*		
		**No**	**Yes**	**Total**	**No**	**Yes**	**Total**
**Current diagnosis or symptoms or treatment**	No	3,576	52	3,628	3,491	109	3,600
	Yes	30	5	35	49	14	63
**Sensitivity**		8.8% (2.9; 19.3)			11.4% (6.4; 18.4)		
**Specificity**		99.2% (98.8; 99.4)			98.6% (98.2; 99.0)		
**Positive Predictive Value**		14.3% (4.8; 30.3)			22.2% (12.7; 34.5)		
**Negative Predictive Value**		98.6% (98.1; 98.9)			97 (96.4; 97.5)		

For peer review only

Table S8: Raw data comparing depression diagnoses based on the Short Mood and Feelings Questionnaire (SMFQ) data from the age 16/17 CCS questionnaire against various definitions derived from the primary care data at this age (*n*=3,213). Note that values with an asterisk have been suppressed for disclosure control purposes as at least one cell has a value < 5. This table also includes sensitivities, specificities, positive predictive values (PPV) and negative predictive values (NPV) for the depression diagnoses based on the SMFQ data from this questionnaire. In these analyses we are treating the ALSPAC data as the reference standard.

		**SMFQ Depression**		
**Primary care definition**		**No**	**Yes**	**Total**
**Current diagnosis**	No	2,719	477	3,196
	Yes	9	8	17
**Sensitivity**		1.6% (0.7; 3.2)		
**Specificity**		99.7% (99.4; 99.8)		
**Positive Predictive Value**		47.1% (23.0; 72.2)		
**Negative Predictive Value**		85.1% (83.8; 86.3)		
		**No**	**Yes**	**Total**
**Current diagnosis, treated**	No	*	*	*
	Yes	*	*	*
**Sensitivity**		*		
**Specificity**		*		
**Positive Predictive Value**		*		
**Negative Predictive Value**		*		
		**No**	**Yes**	**Total**
**Current diagnosis or symptoms or treatment**	No	2,694	451	3,145
	Yes	34	34	68
**Sensitivity**		7.0% (4.9; 9.7)		
**Specificity**		98.8% (98.3; 99.1)		
**Positive Predictive Value**		50% (37.6; 62.4)		
**Negative Predictive Value**		85.7% (84.4; 86.9)		

For peer review only

Table S9: Raw data comparing depression diagnoses based on the Short Mood and Feelings Questionnaire (SMFQ) data from the age 18/19 CCT questionnaire against various definitions derived from the primary care data at this age (*n*=1,982). This table also includes sensitivities, specificities, positive predictive values (PPV) and negative predictive values (NPV) for the depression diagnoses based on the SMFQ data from this questionnaire. In these analyses we are treating the ALSPAC data as the reference standard.

		**SMFQ Depression**		
**Primary care definition**		**No**	**Yes**	**Total**
**Current diagnosis**	No	1,586	372	1,958
	Yes	9	15	24
**Sensitivity**		3.9% (2.2; 6.3)		
**Specificity**		99.4% (98.9; 99.7)		
**Positive Predictive Value**		62.5% (40.6; 81.2)		
**Negative Predictive Value**		81.0% (79.2; 82.7)		
		**No**	**Yes**	**Total**
**Current diagnosis, treated**	No	1,590	374	1,964
	Yes	5	13	18
**Sensitivity**		3.4% (1.8; 5.7)		
**Specificity**		99.7% (99.3; 99.9)		
**Positive Predictive Value**		72.2% (46.5; 90.3)		
**Negative Predictive Value**		81% (79.1; 82.7)		
		**No**	**Yes**	**Total**
**Current diagnosis or symptoms or treatment**	No	1,542	320	1,862
	Yes	53	67	120
**Sensitivity**		17.3% (13.7; 21.5)		
**Specificity**		96.7% (95.7; 97.5)		
**Positive Predictive Value**		55.8% (46.5; 64.9)		
**Negative Predictive Value**		82.8% (81.0; 84.5)		

For peer review only

Table S10: Raw data comparing depression diagnoses based on the Short Mood and Feelings Questionnaire (SMFQ) data from the age 21/22 YPA questionnaire against various definitions derived from the primary care data at this age (*n*=1,298). This table also includes sensitivities, specificities, positive predictive values (PPV) and negative predictive values (NPV) for the depression diagnoses based on the SMFQ data from this questionnaire. In these analyses we are treating the ALSPAC data as the reference standard.

		**SMFQ Depression**		
**Primary care definition**		**No**	**Yes**	**Total**
**Current diagnosis**	No	1,082	181	1,263
	Yes	16	19	35
**Sensitivity**		9.5% (5.8; 14.4)		
**Specificity**		98.5% (97.6; 99.2)		
**Positive Predictive Value**		54.3% (36.6; 71.2)		
**Negative Predictive Value**		85.7% (83.6; 87.6)		
		**No**	**Yes**	**Total**
**Current diagnosis, treated**	No	1,086	182	1,268
	Yes	12	18	30
**Sensitivity**		9.0% (5.4; 13.9)		
**Specificity**		98.9% (98.1; 99.4)		
**Positive Predictive Value**		60% (40.6; 77.3)		
**Negative Predictive Value**		85.6% (83.6; 87.5)		
		**No**	**Yes**	**Total**
**Current diagnosis or symptoms or treatment**	No	1,026	132	1,158
	Yes	72	68	140
**Sensitivity**		34.0% (27.5; 41.0)		
**Specificity**		93.4% (91.8; 94.8)		
**Positive Predictive Value**		48.6% (40.0; 57.2)		
**Negative Predictive Value**		88.6% (86.6; 90.4)		

For peer review only

Table S11: Raw data comparing depression diagnoses based on the Short Mood and Feelings Questionnaire (SMFQ) data from the age 22/23 YPB questionnaire against various definitions derived from the primary care data at this age (*n*=1,326). This table also includes sensitivities, specificities, positive predictive values (PPV) and negative predictive values (NPV) for the depression diagnoses based on the SMFQ data from this questionnaire. In these analyses we are treating the ALSPAC data as the reference standard.

		**SMFQ Depression**		
**Primary care definition**		**No**	**Yes**	**Total**
**Current diagnosis**	No	1,093	202	1,295
	Yes	10	20	30
**Sensitivity**		9.0% (5.6; 13.6)		
**Specificity**		99.1% (98.3; 99.6)		
**Positive Predictive Value**		66.7% (47.2; 82.7)		
**Negative Predictive Value**		84.4% (82.3; 86.3)		
		**No**	**Yes**	**Total**
**Current diagnosis, treated**	No	1,095	205	1,300
	Yes	8	17	25
**Sensitivity**		7.7% (4.5; 12.0)		
**Specificity**		99.3% (98.6; 99.7)		
**Positive Predictive Value**		68.0% (46.5; 85.1)		
**Negative Predictive Value**		84.2% (82.1; 86.2)		
		**No**	**Yes**	**Total**
**Current diagnosis or symptoms or treatment**	No	1,035	161	1,196
	Yes	68	61	129
**Sensitivity**		27.5% (21.7; 33.9)		
**Specificity**		93.8% (92.2; 95.2)		
**Positive Predictive Value**		47.3% (38.4; 56.3)		
**Negative Predictive Value**		86.5% (84.5; 88.4)		

Table S12: Results of the identification in primary care records analysis, based on whether individuals who were diagnosed have having depression or common mental disorders (CMDs) in ALSPAC were also diagnosed based on primary care record data (with primary care diagnosis defined as 'current diagnosis or treatment or symptoms'). Odds ratios are displayed (with 95% confidence intervals). Due to the small sample sizes in some analyses these estimates are rather imprecise, especially regarding the age 15/16 TF3 clinic as very few individuals were classified correctly/diagnosed as depressed/CMD in primary care records. Coefficients are odds ratios derived from univariable logistic regressions and denote the odds of identification relative to the baseline (e.g., for age 17/18 TF4 clinic depression, females have three times greater odds of being identified than males). Note also that when comparing against ALSPAC data (e.g., mother's marital status, parental education, etc.) the sample size for each analysis will vary as the variables come from different data sources, with different levels of completeness. For a graphical illustration of key results, see figure S1.

Variable	*Age 15/16 TF3 clinic*		*Age 16/17 CCS questionnaire*	*Age 17/18 TF4 clinic*		*Age 18/19 CCT questionnaire*	*Age 21/22 YPA questionnaire*	*Age 22/23 YPB questionnaire*
	Dep. (n = 57)	*CMD (n = 123)*	*Dep. (n = 485)*	*Dep. (n = 243)*	*CMD (n = 455)*	*Dep. (n = 387)*	*Dep. (n = 200)*	*Dep. (n = 222)*
Sex (ref = Male)	0.55 (0.08; 3.66)	0.71 (0.20; 2.45)	1.38 (0.56; 3.43)	3.30 (1.33; 8.14)	3.14 (1.61; 6.13)	0.83 (0.44; 1.55)	1.54 (0.78; 3.04)	1.34 (0.68; 2.63)
Age at data collection event (months)	0.81 (0.55; 1.19)	0.89 (0.73; 1.08)	0.93 (0.81; 1.08)	0.98 (0.92; 1.06)	0.98 (0.92; 1.03)	0.98 (0.94; 1.03)	1.02 (0.97; 1.07)	0.98 (0.94; 1.03)
Mother's age at birth (years)	0.89 (0.72; 1.11)	0.91 (0.80; 1.04)	0.96 (0.89; 1.04)	0.95 (0.89; 1.01)	0.96 (0.91; 1.01)	1.01 (0.96; 1.07)	0.90 (0.84; 0.97)	1.00 (0.93; 1.06)
Mother's home ownership status (ref = owned/mort.)	0.63 (0.06; 6.09)	2.23 (0.65; 7.68)	0.91 (0.34; 2.44)	1.65 (0.81; 3.35)	1.31 (0.73; 2.34)	1.41 (0.71; 2.80)	0.92 (0.43; 1.99)	0.80 (0.32; 1.98)
Mother's marital status (ref = married)	1.38 (0.21; 9.07)	2.61 (0.8; 8.48)	0.77 (0.29; 2.06)	1.48 (0.76; 2.87)	0.98 (0.57; 1.72)	1.25 (0.67; 2.36)	1.43 (0.68; 3.01)	1.02 (0.45; 2.28)
Mother's age at first pregnancy (years)	1.16 (0.88; 1.53)	0.89 (0.77; 1.03)	1.00 (0.93; 1.08)	0.93 (0.87; 0.99)	0.96 (0.91; 1.00)	1.00 (0.95; 1.05)	0.97 (0.91; 1.02)	0.99 (0.93; 1.05)
Mother's parity at child's birth (ref = first child)	0.38 (0.04; 3.91)	1.49 (0.41; 5.40)	0.68 (0.32; 1.43)	0.92 (0.50; 1.70)	1.2 (0.73; 1.96)	1.26 (0.72; 2.21)	0.84 (0.45; 1.56)	1.04 (0.56; 1.92)
Mother's highest education level (ref = CSE/Voc./O level)	NC	0.35 (0.07; 1.66)	0.83 (0.38; 1.81)	0.78 (0.41; 1.47)	1.09 (0.66; 1.80)	0.90 (0.51; 1.57)	1.10 (0.58; 2.11)	1.26 (0.66; 2.40)
Father's highest education level (ref = CSE/Voc./O level)	4.06 (0.39; 42.49)	2.32 (0.63; 8.51)	0.77 (0.35; 1.70)	1.16 (0.61; 2.21)	1.18 (0.71; 1.98)	1.20 (0.68; 2.14)	0.85 (0.45; 1.61)	0.67 (0.36; 1.26)
Mother's social class (ref = I/II)	NC	NC	0.96 (0.44; 2.10)	1.33 (0.67; 2.65)	0.86 (0.50; 1.47)	0.90 (0.50; 1.64)	0.77 (0.39; 1.54)	0.43 (0.22; 0.85)
Father's social class (ref = I/II)	0.56 (0.08; 3.66)	1.04 (0.33; 3.35)	1.74 (0.76; 3.99)	1.57 (0.81; 3.05)	1.00 (0.59; 1.68)	1.24 (0.70; 2.21)	1.31 (0.69; 2.48)	0.77 (0.40; 1.47)
Child's ethnic background (ref = white)	NC	0.91 (0.10; 7.90)	0.87 (0.11; 6.80)	0.37 (0.04; 2.99)	0.67 (0.19; 2.32)	0.75 (0.16; 3.42)	1.23 (0.20; 7.59)	1.22 (0.30; 5.06)
Child's education (# A-C GCSE equivalents)*	0.93 (0.72; 1.22)	0.92 (0.79; 1.06)	0.89 (0.80; 0.98)	0.97 (0.90; 1.05)	0.99 (0.92; 1.05)	0.94 (0.88; 1.01)	1.02 (0.94; 1.10)	1.00 (0.92; 1.08)
CIS-R depression score (ref = 1 for depression model; ref = 0 for CMD model)	NA	NA	NA	2: 1.44 (0.54; 3.83)<br>3: 2.21 (0.88; 5.54)<br>4: 3.78 (1.5; 9.53)	1: 1.97 (0.83; 4.70)<br>2: 2.85 (1.21; 6.67)	NA	NA	NA

For peer review only - http://bmjopen.bmj.com/site/about/guidelines.xhtml

					3: 5.73 (2.57; 12.80)<br>4: 10.08 (4.40; 23.09)			
CIS-R diagnosis of moderate depressive episode (ref = no)	NA	NA	NA	2.49 (1.26; 4.92)	3.23 (2.00; 5.22)	NA	NA	NA
CIS-R diagnosis of severe depressive episode (ref = no)	NA	NA	NA	2.96 (1.39; 6.29)	5.01 (2.44; 10.30)	NA	NA	NA
CIS-R GAD symptoms (ref = no)	NA	NA	NA	2.11 (1.15; 3.88)	1.49 (0.93; 2.38)	NA	NA	NA
CIS-R total score	NA	NA	NA	1.09 (1.04; 1.15)	1.11 (1.07; 1.15)	NA	NA	NA
DAWBA predicted depression (ref = ~50%)	>70%: 1.38 (0.14; 13.95)	~0.5%: 0.29 (0.03; 2.58)<br>~15%: 2.32 (0.67; 8.05)<br>>70%: 1.08 (0.11; 10.47)	NA	NA	NA	NA	NA	NA
DAWBA predicted anxiety (ref = ~50%)	~3%: 2.53 (0.25; 25.39)	~3%: 1.45 (0.41; 5.15)	NA	NA	NA	NA	NA	NA
Total SMFQ score	NA	NA	1.11 (1.02; 1.21)	NA	NA	1.09 (1.03; 1.16)	1.14 (1.06; 1.22)	1.08 (1.01; 1.16)
Historical depression diagnosis	NC	NC	14.03 (1.91; 102.9)	8.12 (2.03; 32.52)	14.56 (4.57; 46.38)	13.86 (4.20; 45.71)	3.27 (1.52; 7.03)	3.07 (1.46; 6.46)
Historical anxiety diagnosis	34.00 (2.36; 489.96)	8.92 (1.15; 69.18)	7.22 (2.55; 20.43)	2.34 (0.71; 7.68)	2.24 (0.97; 5.18)	6.09 (2.47; 15.01)	2.81 (1.31; 6.01)	1.95 (0.87; 4.33)
Current anxiety diagnosis	NC	NA	13.59 (4.28; 43.16)	16.94 (1.94; 148.16)	NA	9.76 (3.68; 25.87)	10.16 (2.79; 37.09)	5.07 (1.75; 14.63)
Historical depression symptoms	NC	NC	4.69 (1.60; 13.7)	5.50 (2.21; 13.65)	5.34 (2.58; 11.08)	4.71 (2.29; 9.68)	5.6 (2.86; 10.97)	4.75 (2.52; 8.97)
Historical somatic symptoms	4.08 (0.34; 48.86)	8.32 (1.92; 36.05)	3.77 (1.51; 9.40)	1.72 (0.82; 3.61)	1.66 (0.92; 2.98)	2.53 (1.38; 4.66)	3.77 (2.01; 7.07)	2.59 (1.37; 4.9)
Current somatic symptoms	NC	1.32 (0.15; 11.85)	2.47 (0.69; 8.89)	3.50 (1.32; 9.29)	6.18 (2.78; 13.76)	3.73 (1.65; 8.46)	1.78 (0.62; 5.13)	12.9 (3.50; 47.57)
Historical general symptoms	0.68 (0.07; 6.60)	1.78 (0.51; 6.26)	2.63 (1.27; 5.45)	3.35 (1.79; 6.27)	3.46 (2.07; 5.75)	4.04 (2.32; 7.06)	3.62 (1.91; 6.88)	1.79 (0.97; 3.31)
Current general symptoms	3.00 (0.27; 33.64)	1.12 (0.13; 9.85)	2.20 (0.62; 7.84)	3.99 (1.29; 12.4)	2.38 (1.02; 5.54)	2.15 (0.90; 5.14)	3.67 (1.04; 13.02)	3.35 (1.08; 10.40)
Historical anti-depressant treatment	NC	NC	11.89 (3.04; 46.61)	3.97 (1.53; 10.31)	7.46 (3.22; 17.26)	23.37 (9.47; 57.66)	9.87 (5.00; 19.44)	9.59 (4.83; 19.04)
Historical anti-anxiety treatment	NC	8.31 (0.49; 140.9)	3.46 (0.71; 26.98)	1.80 (0.58; 5.6)	3.74 (1.5; 9.34)	6.24 (2.78; 14.01)	4.03 (1.90; 8.55)	5.49 (2.68; 11.27)
Current anti-anxiety treatment	NC	NA	6.98 (1.23; 39.59)	6.80 (2.39; 19.32)	NA	12.90 (5.02; 33.15)	10.16 (2.79; 37.08)	5.40 (1.73; 16.84)
# of GP consultations per year	1.07 (0.86; 1.34)	1.06 (0.93; 1.20)	1.17 (1.11; 1.23)	1.25 (1.16; 1.34)	1.24 (1.17; 1.31)	1.13 (1.07; 1.18)	1.25 (1.15; 1.35)	1.23 (1.15; 1.32)

For peer review only

# of GP prescriptions per year	0.93 (0.64; 1.37)	0.88 (0.69; 1.11)	1.22 (1.11; 1.35)	1.25 (1.14; 1.38)	1.29 (1.19; 1.39)	1.25 (1.14; 1.36)	1.44 (1.26; 1.64)	1.37 (1.22; 1.54)
Asthma diagnosis	1.64 (0.25; 10.85)	1.28 (0.37; 4.41)	1.77 (0.83; 3.76)	0.74 (0.37; 1.48)	0.77 (0.44; 1.36)	1.44 (0.79; 2.61)	1.00 (0.51; 1.94)	1.82 (0.95; 3.51)
Eczema diagnosis	1.07 (0.16; 6.95)	0.57 (0.17; 1.93)	1.60 (0.79; 3.27)	1.35 (0.71; 2.55)	1.13 (0.69; 1.85)	1.36 (0.79; 2.34)	1.18 (0.65; 2.14)	0.72 (0.39; 1.33)
Eating disorder	NC	5.89 (0.89; 38.83)	5.58 (1.04; 29.87)	3.31 (0.92; 11.88)	5.28 (1.73; 16.13)	2.27 (0.76; 6.75)	1.12 (0.32; 3.95)	1.96 (0.60; 6.44)
History of mental health issues	NC	2.72 (0.26; 28.08)	1.49 (0.18; 12.11)	12.25 (2.47; 60.76)	7.96 (2.28; 27.85)	8.54 (2.70; 27.02)	8.67 (1.79; 42.05)	6.83 (1.71; 27.34)
Self-harm	5.11 (0.70; 37.06)	3.97 (0.90; 17.61)	4.15 (1.44; 11.97)	3.72 (1.61; 8.57)	4.46 (2.21; 9.01)	3.61 (1.70; 7.69)	3.23 (1.30; 8.00)	4.31 (1.56; 11.92)
Any other mental health issues	NC	9.64 (1.73; 53.63)	2.47 (0.69; 8.89)	1.78 (0.63; 5.04)	2.86 (1.25; 6.54)	2.08 (0.83; 5.23)	1.12 (0.45; 2.83)	1.58 (0.63; 3.99)
Referral to mental health services	1.92 (0.18; 20.11)	7.01 (1.89; 25.97)	3.39 (1.37; 8.40)	5.84 (2.83; 12.07)	5.76 (3.19; 10.42)	6.85 (3.45; 13.61)	3.71 (1.79; 7.65)	4.22 (2.08; 8.56)
Smoking (or ex-smoker)	NC	2.10 (0.40; 11.08)	2.08 (0.90; 4.82)	2.92 (1.54; 5.52)	3.38 (2.01; 5.71)	2.26 (1.26; 4.05)	1.24 (0.67; 2.28)	1.15 (0.61; 2.16)

NA = Not applicable (either because measure not available at that time-point, or because the variable is part of the GP diagnosis variable; e.g., current anti-anxiety treatment is part of the 'current diagnosis or symptoms or treatment' definition of CMDs)

NC = Not calculable (due to collinearity in model as a result of lack of data). Variables where half or more of all analyses were not calculable due to collinearity have been removed from this table, as sample sizes are likely to be too low to be informative. These variables are: historical phobia diagnosis, current phobia diagnosis, historical anxiety symptoms, current anxiety symptoms, ADHD, conduct disorder, autism spectrum disorder, substance abuse, family history of mental illness, family history of depression, personal history of depression and other psychological illness.

For peer review only

Figure S1 (following page): Graphical summary of key results of the identification in primary care records analysis, based on whether individuals who were diagnosed have having depression or common mental disorders (CMDs) in ALSPAC were also diagnosed based on primary care record data (with primary care diagnosis defined as 'current diagnosis or treatment or symptoms'). Values for depression are displayed in black, and common mental disorders are in red. Odds ratios and 95% confidence intervals are displayed on the y-axis (on the log scale), with time point along the x-axis (going forwards in time, from the age 15/16 TF3 clinic to the age 22/23 YPB questionnaire. Due to the small sample sizes in some analyses these estimates are rather imprecise, especially regarding the 15/16 clinic as very few individuals were classified correctly/diagnosed as depressed/CMD in primary care records. Coefficients are odds ratios derived from univariable logistic regressions and denote the odds of identification relative to the baseline (e.g., for age 17/18 TF4 clinic depression, females have three times greater odds of being identified than males). Note also that when comparing against ALSPAC data (e.g., mother's marital status, parental education, etc.) the sample size for each analysis will vary as the variables come from different data sources, with different levels of completeness. For a full list of results, see table S11.

For peer review only

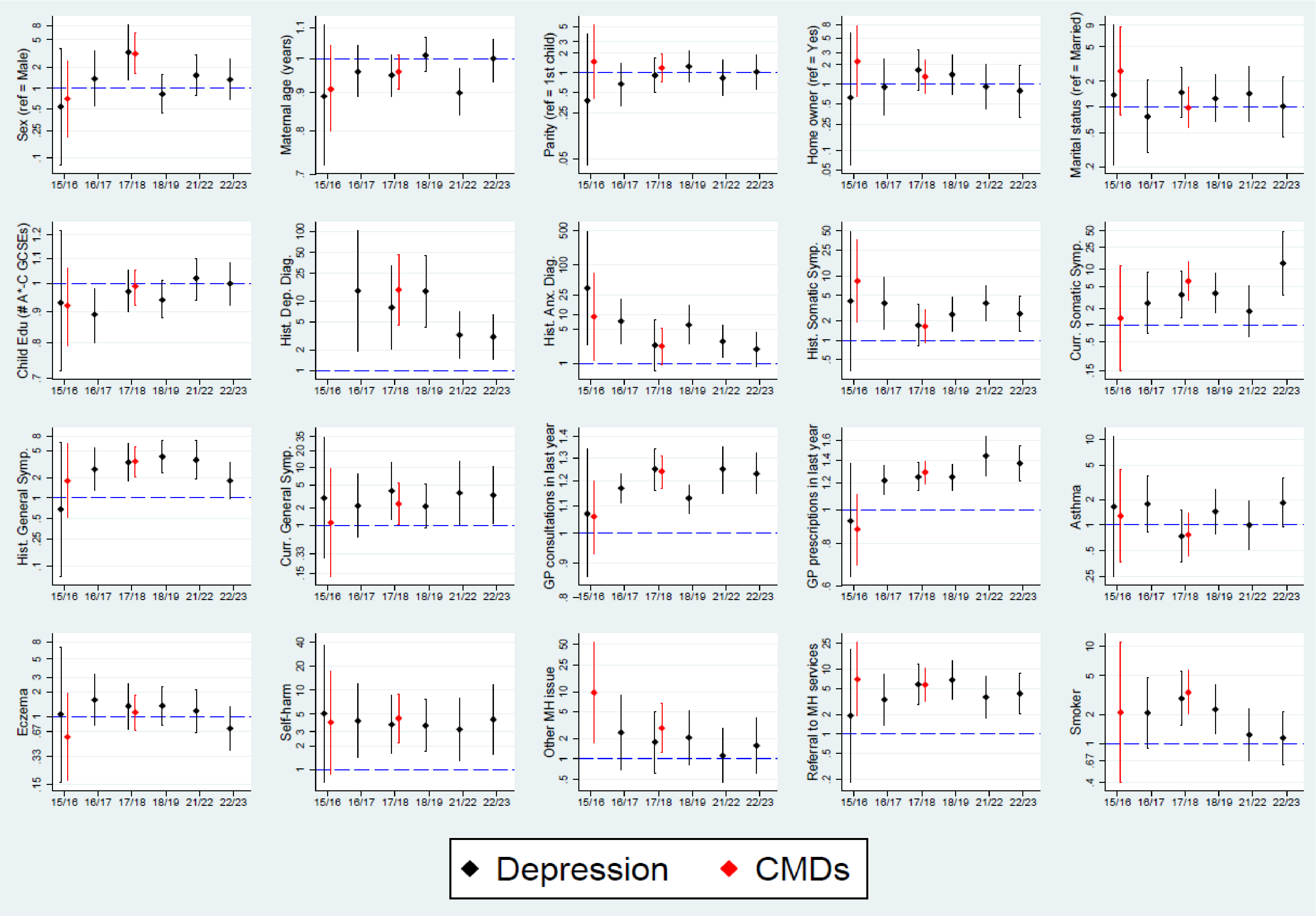
Sex (ref = Male)
Maternal age (years)
Parity (ref = 1st child)
Home owner (ref = Yes)
Marital status (ref = Married)
Child Edu (# A*-C GCSEs)
Hist. Dep. Diag.
Hist. Anx. Diag.
Hist. Somatic Symp.
Curr. Somatic Symp.
Hist. General Symp.
Curr. General Symp.
GP consultations in last year
GP prescriptions in last year
Asthma
Eczema
Self-harm
Other MH issue
Referral to MH services
Smoker
15/16
16/17
17/18
18/19
21/22
22/23
Depression
CMDs

Table S13: Penalised coefficients from the optimal lasso prediction models, predicting ALSPAC questionnaire-derived depression or common mental disorder (CMD) cases using only linked primary care record data. Coefficients are log-odds estimates. Note that as the estimates are derived from lasso models used for model prediction, standard errors are not calculated.

	**Age 15/16 TF3 clinic**		**Age 16/17 CCS quest**	**Age 17/18 TF4 clinic**		**Age 18/19 CCT quest**	**Age 21/22 YPA quest**	**Age 22/23 YPB quest**
	Dep.	CMD	Dep.	Dep.	CMD	Dep.	Dep.	Dep.
**Constant**	-4.307	-3.788	-2.602	-3.240	-5.966	-1.864	-2.036	-5.805
**Self-harm status (ref: no)**	2.297	1.225	0.476	1.469	1.301	1.536	-	0.538
**Historical general symptoms (ref: no)**	0.464	0.143	0.204	-	-	-	-	-
**Mean number of GP consultations**	0.020	0.036	0.030	0.046	0.019	0.004	-	-
**Historical depression diagnosis (ref: no)**	0.840	-	-	-	-	-	0.421	0.071
**Current depression diagnosis (ref: no)**	0.478	1.445	0.217	0.472	0.680	0.690	-	0.606
**Sex (ref: male)**	-	0.544	0.777	0.437	0.493	0.449	-	-
**Eating disorder (ref: no)**	-	0.748	-	0.791	-	-	-	-
**Referral to mental health services (ref: no)**	-	0.242	1.042	-	0.525	0.220	0.539	0.290
**Current phobia diagnosis (ref: no)**	-	1.434	-	-	-	2.281	-	-
**Current anxiety diagnosis (ref: no)**	-	0.349	1.035	-	0.016	1.057	-	-
**Current antidepressant use (ref: no)**	-	0.312	-	0.808	0.618	0.725	0.730	0.820
**Current depression symptoms (ref: no)**	-	-	0.218	1.156	0.760	-	0.033	0.502
**Smoking status (ref: no)**	-	-	0.466	0.058	-	-	-	-
**Historical depression symptoms (ref: no)**	-	-	0.469	-	0.388	0.457	0.180	0.540
**Historical somatic symptoms (ref: no)**	-	-	0.125	-	0.185	0.016	-	-
**Autism spectrum disorder (ref: no)**	-	-	2.131	-	-	-	-	0.702
**Family history of depression (ref: no)**	-	-	2.610	-	-	-	-	-
**Conduct disorder (ref: no)**	-	-	0.890	-	-	-	-	-
**ADHD (ref: no)**	-	-	-0.622	-	-	-	-	-
**Substance abuse (ref: no)**	-	-	0.881	-	-	-	-	-
**Historical phobia diagnosis (ref: no)**	-	-	0.373	-	-	-	-	0.447
**Historical anxiety symptoms (ref: no)**	-	-	0.292	-	-	-	-	-0.634
**Historical anti-anxiety meds (ref: no)**	-	-	-	0.630	0.170	0.426	-	-
**Current anti-anxiety meds (ref: no)**	-	-	-	0.251	-	-	0.122	-
**Eczema (ref: no)**	-	-	-	0.027	-	-	-	-

**Historical antidepressant use (ref: no)**	-	-	-	0.058	0.135	-0.066	0.613	0.688
**Historical anxiety diagnosis (ref: no)**	-	-	-	-	0.524	-	-	-
**Age (months)**	-	-	-	-	0.017	-	-	0.014
**Current general symptoms (ref: no)**	-	-	-	-	0.189	-	-	-
**Current somatic symptoms (ref: no)**	-	-	-	-	-	0.613	-	-
**History of depression (ref: no)**	-	-	-	-	-	0.808	-	-
**History of mental health issues (ref: no)**	-	-	-	-	-	-0.198	-	-
**Other psychological illness (ref: no)**	-	-	-	-	-	-0.013	-	-

For peer review only

Table S14: Full models to estimate the predicted probability of ALSPAC questionnaire-derived depression or common mental disorders (CMDs) at each time-point, based on the best-fitting lasso model using only linked primary care record data as predictors. Code is provided in Stata syntax.

**Time-point (*n*)**	**Diagnosis**	**Model to estimate predicted probability of diagnosis**
**Age 15/16 TF3 clinic (*n* = 3,663)**	Depression	gen TF3_dep_DAWBA = (exp(-4.306898 + (selfharm_TF3 * 2.297141) + (hist_gensym_TF3 * 0.4635765) + (consult_mean_TF3 * 0.0201705) + (hist_depress_TF3 * 0.8404444) + (curr_depress_TF3 * 0.4780882))) / (1 (exp(-4.306898 + (selfharm_TF3 * 2.297141) + (hist_gensym_TF3 * 0.4635765) + (consult_mean_TF3 * 0.0201705) + (hist_depress_TF3 * 0.8404444) + (curr_depress_TF3 * 0.4780882))))
	CMD	gen TF3_CMD_DAWBA = (exp(-3.78843 + (sex * 0.5437601) + (selfharm_TF3 * 1.224612) + (consult_mean_TF3 * 0.0356085) + (curr_depress_TF3 * 1.444507) + (eatdisorder_TF3 * 0.7475241) + (hist_gensym_TF3 * 0.1431262) + (referral_TF3 * 0.2418507) + (curr_phob_TF3 * 1.434107) + (curr_anx_TF3 * 0.3485559) + (curr_depdrugs_TF3 * 0.3121066))) / (1 + (exp(-3.78843 + (sex * 0.5437601) + (selfharm_TF3 * 1.224612) + (consult_mean_TF3 * 0.0356085) + (curr_depress_TF3 * 1.444507) + (eatdisorder_TF3 * 0.7475241) + (hist_gensym_TF3 * 0.1431262) + (referral_TF3 * 0.2418507) + (curr_phob_TF3 * 1.434107) + (curr_anx_TF3 * 0.3485559) + (curr_depdrugs_TF3 * 0.3121066))))
**Age 16/17 CCS quest (*n* = 3,213)**	Depression	gen CCS_dep_SMFQ = (exp(-2.602106 + (sex * 0.7768598) + (referral_CCS * 1.042151) + (asd_CCS * 2.131247) + (smoke_CCS * 0.4664435) + (consult_mean_CCS * 0.0301841) + (curr_anx_CCS * 1.03486) + (fhdep_CCS * 2.610094) + (hist_gensym_CCS * 0.203526) + (selfharm_CCS * 0.4762654) + (conduct_CCS * 0.8898849) + (hist_depsymp_CCS * 0.4685372) + (adhd_CCS * -0.6223789) + (hist_somsym_CCS * 0.1245751) + (alcdrug_CCS * 0.8809454) + (hist_phob_CCS * 0.3725191) + (curr_depsymp_CCS * 0.2181478) + (curr_depress_CCS * 0.2167674) + (hist_anxsymp_CCS * 0.2916632))) / (1 + (exp(-2.602106 + (sex * 0.7768598) + (referral_CCS * 1.042151) + (asd_CCS * 2.131247) + (smoke_CCS * 0.4664435) + (consult_mean_CCS * 0.0301841) + (curr_anx_CCS * 1.03486) + (fhdep_CCS * 2.610094) + (hist_gensym_CCS * 0.203526) + (selfharm_CCS * 0.4762654) + (conduct_CCS * 0.8898849) + (hist_depsymp_CCS * 0.4685372) + (adhd_CCS * -0.6223789) + (hist_somsym_CCS * 0.1245751) + (alcdrug_CCS * 0.8809454) + (hist_phob_CCS * 0.3725191) + (curr_depsymp_CCS * 0.2181478) + (curr_depress_CCS * 0.2167674) + (hist_anxsymp_CCS * 0.2916632))))
**Age 17/18 TF4 clinic (*n* = 3,084)**	Depression	gen TF4_dep_CISR = (exp(-3.239794 + (sex * 0.436772) + (selfharm_TF4 * 1.469416) + (consult_mean_TF4 * 0.0459718) + (referral_TF4 * 0.7906288) + (curr_depsymp_TF4 * 1.155598) + (curr_depdrugs_TF4 * 0.8078427) + (hist_anxdrugs_TF4 * 0.6298965) + (curr_depress_TF4 * 0.4717727) + (curr_anxdrugs_TF4 * 0.2511227) + (smoke_TF4 * 0.0582065) + (eczema * 0.0274914) + (hist_depdrugs_TF4 * 0.0581372))) / (1 + (exp(-3.239794 + (sex * 0.436772) + (selfharm_TF4 * 1.469416) + (consult_mean_TF4 * 0.0459718) + (referral_TF4 * 0.7906288) + (curr_depsymp_TF4 * 1.155598) + (curr_depdrugs_TF4 * 0.8078427) + (hist_anxdrugs_TF4 * 0.6298965) + (curr_depress_TF4 * 0.4717727) + (curr_anxdrugs_TF4 * 0.2511227) + (smoke_TF4 * 0.0582065) + (eczema * 0.0274914) + (hist_depdrugs_TF4 * 0.0581372))))
	CMD	gen TF4_CMD_CISR = (exp(-5.96604 + (sex * 0.4931608) + (selfharm_TF4 * 1.301296) + (referral_TF4 * 0.5245662) + (curr_depsymp_TF4 * 0.7602904) + (curr_depdrugs_TF4 * 0.6179576) + (consult_mean_TF4 * 0.0194573) + (hist_anx_TF4 *

		0.5237928) + (TF4_agemths * 0.0168863) + + (curr_depress_TF4 * 0.6795285) + (hist_depsymp_TF4 * 0.3876382) + (hist_somsym_TF4 * 0.184908) + (curr_gensym_TF4 * 0.189229) + (hist_anxdrugs_TF4 * 0.1699545) + (hist_depdrugs_TF4 * 0.1345062) + (curr_anx_TF4 * 0.0156222))) / (1 + (exp(-5.96604 + (sex * 0.4931608) + (selfharm_TF4 * 1.301296) + (referral_TF4 * 0.5245662) + (curr_depsymp_TF4 * 0.7602904) + (curr_depdrugs_TF4 * 0.6179576) + (consult_mean_TF4 * 0.0194573) + (hist_anx_TF4 * 0.5237928) + (TF4_agemths * 0.0168863) + + (curr_depress_TF4 * 0.6795285) + (hist_depsymp_TF4 * 0.3876382) + (hist_somsym_TF4 * 0.184908) + (curr_gensym_TF4 * 0.189229) + (hist_anxdrugs_TF4 * 0.1699545) + (hist_depdrugs_TF4 * 0.1345062) + (curr_anx_TF4 * 0.0156222))))
**Age 18/19 CCT quest (*n* = 1,982)**	Depression	gen CCS_dep_SMFQ = (exp(-1.864214 + (selfharm_CCT * 1.536287) + (sex * 0.4488152) + (curr_depdrugs_CCT * 0.7249568) + (curr_anx_CCT * 1.056577) + (curr_somsym_CCT * 0.6129295) + (curr_phob_CCT * 2.280971) + (hist_depsymp_CCT * 0.4572833) + (hist_anxdrugs_CCT * 0.4263218) + (curr_depress_CCT * 0.6901121) + (referral_CCT * 0.220459) + (histdep_CCT * 0.807517) + (histmh_CCT * -0.197546) + (consult_mean_CCT * 0.0.0041373) + (hist_depdrugs_CCT * -0.0660863) + (hist_somsym_CCT * 0.015973) + (CCT_agemths * -0.0003042) + (psych_other_CCT * -0.0130987))) / (1 + (exp(-1.864214 + (selfharm_CCT * 1.536287) + (sex * 0.4488152) + (curr_depdrugs_CCT * 0.7249568) + (curr_anx_CCT * 1.056577) + (curr_somsym_CCT * 0.6129295) + (curr_phob_CCT * 2.280971) + (hist_depsymp_CCT * 0.4572833) + (hist_anxdrugs_CCT * 0.4263218) + (curr_depress_CCT * 0.6901121) + (referral_CCT * 0.220459) + (histdep_CCT * 0.807517) + (histmh_CCT * -0.197546) + (consult_mean_CCT * 0.0.0041373) + (hist_depdrugs_CCT * -0.0660863) + (hist_somsym_CCT * 0.015973) + (CCT_agemths * -0.0003042) + (psych_other_CCT * -0.0130987))))
**Age 21/22 YPA quest (*n* = 1,298)**	Depression	gen YPA_dep_SMFQ = (exp(-2.036359 + (curr_depdrugs_YPA * 0.7299155) + (hist_depdrugs_YPA * 0.6132707) + (referral_YPA * 0.5390764) + (hist_depress_YPA * 0.4207832) + (hist_depsymp_YPA * 0.1804129) + (curr_anxdrugs_YPA * 0.122329) + (curr_depsymp_YPA * 0.033265))) / (1 + (exp(-2.036359 + (curr_depdrugs_YPA * 0.7299155) + (hist_depdrugs_YPA * 0.6132707) + (referral_YPA * 0.5390764) + (hist_depress_YPA * 0.4207832) + (hist_depsymp_YPA * 0.1804129) + (curr_anxdrugs_YPA * 0.122329) + (curr_depsymp_YPA * 0.033265))))
**Age 22/23 YPB quest (*n* = 1,325)**	Depression	gen YPB_dep_SMFQ = (exp(-5.804938 + (hist_depdrugs_YPB * 0.6876923) + (curr_depdrugs_YPB * 0.8200612) + (hist_depsymp_YPB * 0.5401723) + (curr_depress_YPB * 0.6056239) + (hist_anxsymp_YPB * -0.6341232) + (selfharm_YPB * 0.5377375) + (YPB_agemths * 0.0136081) + (curr_depsymp_YPB * 0.502017) + (referral_YPB * 0.2895768) + (asd_YPB * 0.7015883) + (hist_phob_YPB * 0.4473284) + (hist_depress_YPB * 0.0714968))) / (1 + (exp(-5.804938 + (hist_depdrugs_YPB * 0.6876923) + (curr_depdrugs_YPB * 0.8200612) + (hist_depsymp_YPB * 0.5401723) + (curr_depress_YPB * 0.6056239) + (hist_anxsymp_YPB * -0.6341232) + (selfharm_YPB * 0.5377375) + (YPB_agemths * 0.0136081) + (curr_depsymp_YPB * 0.502017) + (referral_YPB * 0.2895768) + (asd_YPB * 0.7015883) + (hist_phob_YPB * 0.4473284) + (hist_depress_YPB * 0.0714968))))

Table S15: In-sample and out-of-sample deviance ratios for common mental disorders (CMDs) or depression at each time-point. Deviance ratios are taken from logistic cross-validation lasso models. The full models are based on the set of all the primary care variables described in table S3. The 'diagnosis only' models contain just the relevant 'current' diagnosis variables (for depression models, this is just current depression diagnosis; for CMD models, this is current depression, anxiety and phobia diagnosis). The 'diagnosis, symptoms and treatment' models contain the relevant 'current' diagnosis, symptoms or treatment variables (for depression models, this is current depression diagnosis, current depressive symptoms and current antidepressant use; for CMD models, this is current depression, anxiety and phobia diagnosis, current depressive and anxiety symptoms, and current antidepressant and anti-anxiety medications).

**Time-point (*n*)**	**Diagnosis**	**Lasso model**	**Sample**	**Deviance ratio**
**Age 15/16 TF3 clinic (*n* = 3,663)**	Depression	Full	Training	9.9%
			Validation	-1.3%
		Diagnosis only	Training	0.0%
			Validation	-0.9%
		Diagnosis, symptoms and treatment	Training	0.0%
			Validation	-0.9%
	CMD	Full	Training	8.4%
			Validation	2.9%
		Diagnosis only	Training	2.0%
			Validation	-1.2%
		Diagnosis, symptoms and treatment	Training	1.6%
			Validation	-1.5%
**Age 16/17 CCS questionnaire (*n* = 3,213)**	Depression	Full	Training	8.3%
			Validation	4.3%
		Diagnosis only	Training	0.4%
			Validation	0.3%
		Diagnosis, symptoms and treatment	Training	0.8%
			Validation	1.8%
**Age 17/18 TF4 clinic (*n* = 3,084)**	Depression	Full	Training	14.6%
			Validation	7.8%
		Diagnosis only	Training	2.4%
			Validation	1.1%
		Diagnosis, symptoms and treatment	Training	7.6%
			Validation	6.9%
	CMD	Full	Training	9.2%
			Validation	7.8%
		Diagnosis only	Training	3.8%
			Validation	4.9%
		Diagnosis, symptoms and treatment	Training	3.8%
			Validation	5.2%
**Age 18/19 CCT questionnaire (*n* = 1,982)**	Depression	Full	Training	9.0%
			Validation	8.0%
		Diagnosis only	Training	1.3%
			Validation	0.8%

		Diagnosis, symptoms and treatment	Training	3.2%
			Validation	5.3%
**Age 21/22 YPA questionnaire (*n* = 1,298)**	Depression	Full	Training	11.7%
			Validation	12.6%
		Diagnosis only	Training	3.3%
			Validation	1.6%
		Diagnosis, symptoms and treatment	Training	8.8%
			Validation	10.0%
**Age 22/23 YPB questionnaire (*n* = 1,325)**	Depression	Full	Training	13.4%
			Validation	9.1%
		Diagnosis only	Training	3.5%
			Validation	2.7%
		Diagnosis, symptoms and treatment	Training	7.9%
			Validation	5.0%

peer review only

Table S16: Sensitivities and specificities from the 40% validation sample, assessing how well the lasso model predicts common mental disorders (CMDs) or depression in ALSPAC across each of the time-points. Sensitivities and specificities are also given for the three ‘CMD/depression’ definitions using only primary care diagnosis, symptoms and/or treatment data (all using the same 40% validation sample). In these analyses we are treating the ALSPAC data as the reference standard. Note that for disclosure control purposes, statistics calculated where at least one cell in the cross-tabulation has a value <5 have been suppressed and replaced with a ‘<’ or ‘>’ summary statistic.

	**Sensitivity**	**Specificity**
**Age 15/16 TF3 clinic depression – DAWBA (*n*=1,465; *n* with ALSPAC depression = 19)**		
**Lasso prediction model**	0.0% (0.0; 17.6)[a]	100.0% (99.7; 100.0)[a]
**Current diagnosis**	0.0% (0.0; 17.6)[a]	>99.0%
**Current diagnosis, treated**	0.0% (0.0; 17.6)[a]	100.0% (99.7; 100.0)[a]
**Current diagnosis or symptoms or treatment**	<10.0%	>99.0%
**Age 15/16 TF3 clinic CMD – DAWBA (*n*=1,465; *n* with ALSPAC CMD = 40)**		
**Lasso prediction model**	0.0% (0.0; 8.8)[a]	100.0% (99.7; 100.0)[a]
**Current diagnosis**	<5.0%	>99.0%
**Current diagnosis, treated**	0.0% (0.0; 8.8)[a]	100.0% (99.7; 100.0)[a]
**Current diagnosis or symptoms or treatment**	<10.0%	>98.0%
**Age 16/17 CCS questionnaire depression – SMFQ (*n*=1,285; *n* with ALSPAC depression = 197)**		
**Lasso prediction model**	3.5% (1.4; 7.2)	99.0% (98.2; 99.5)
**Current diagnosis**	<3.0%	>99.0%
**Current diagnosis, treated**	<3.0%	>99.0%
**Current diagnosis or symptoms or treatment**	8.1% (4.7; 12.9)	99.0% (98.2; 99.5)
**Age 17/18 TF4 clinic depression – CIS-R (*n*=1,234; *n* with ALSPAC depression = 100)**		
**Lasso prediction model**	6.0% (2.2; 12.6)	99.1% (98.4; 99.6)
**Current diagnosis**	5.0% (1.6; 11.3)	99.3% (98.6; 99.7)
**Current diagnosis, treated**	<5.0%	>99.0%
**Current diagnosis or symptoms or treatment**	26.0% (17.7; 35.7)	97.3% (96.1; 98.1)
**Age 17/18 TF4 clinic CMD – CIS-R (*n*=1,234; *n* with ALSPAC CMD = 187)**		
**Lasso prediction model**	8.0% (4.6; 12.9)	99.1% (98.4; 99.6)
**Current diagnosis**	7.0% (3.8; 11.6)	98.5% (97.5; 99.1)
**Current diagnosis, treated**	3.7% (1.5; 7.6)	99.4% (98.8; 99.8)
**Current diagnosis or symptoms or treatment**	23.0% (17.2; 29.7)	96.4% (95.1; 97.4)
**Age 18/19 CCT questionnaire depression – SMFQ (*n*=793; *n* with ALSPAC depression = 152)**		
**Lasso prediction model**	13.2% (8.2; 19.6)	98.1% (96.8; 99.0)
**Current diagnosis**	3.9% (1.5; 8.4)	99.2% (98.2; 99.7)
**Current diagnosis, treated**	<5.0%	>99.0%
**Current diagnosis or symptoms or treatment**	22.4% (16.0; 29.8)	97.7% (96.2; 98.7)

**Age 21/22 YPA questionnaire depression – SMFQ (*n*=519; *n* with ALSPAC depression = 82)**		
**Lasso prediction model**	<10.0%	>99.0%
**Current diagnosis**	7.3% (2.7; 15.2)	98.6% (97.0; 99.5)
**Current diagnosis, treated**	6.1% (2.0; 13.7)	98.9% (97.4; 99.6)
**Current diagnosis or symptoms or treatment**	36.6% (26.2; 48.0)	93.6% (90.9; 95.7)
**Age 22/23 YPB questionnaire depression – SMFQ (*n*=530; *n* with ALSPAC depression = 92)**		
**Lasso prediction model**	16.3% (9.4; 25.5)	98.2% (96.4; 99.2)
**Current diagnosis**	<10.0%	>99.0%
**Current diagnosis, treated**	<10.0%	>99.0%
**Current diagnosis or symptoms or treatment**	25.0% (16.6; 35.1)	93.8% (91.2; 95.9)

[a] One-sided 97.5% confidence interval.
DAWBA: Development and Well-Being Assessment; *SMFQ*: Short Mood and Feelings Questionnaire; *CIS-R:* Clinical Interview Schedule – Revised.

For peer review only

Table S17: Examining the association between primary care depression and common mental disorder (CMD) diagnoses and having ALSPAC data at each time point. For each time point, we present three sets of analyses: i) unadjusted models exploring the univariable associations between having ALSPAC data and both depression and CMDs; ii) models adjusting for sex; and iii) models adjusting for both sex and maternal education (a proxy for SEP; operationalised as a binary variable with the categories 'CSE/Vocational/O level' vs 'A level/Degree'). All results are odds ratios with 95% confidence intervals in brackets.

	**Depression**	**CMDs**
**Age 15/16 TF3 clinic**		
Unadjusted model (n = 8,565)	1.17 (0.42; 3.23)	1.2 (0.71; 2.04)
Adjusted for sex (n = 8,565)	1.16 (0.42; 3.21)	1.14 (0.67; 1.93)
Adjusted for sex & maternal education (n = 7,342)	1.38 (0.41; 4.66)	1.05 (0.58; 1.89)
**Age 16/17 CCS questionnaire**		
Unadjusted model (n = 8,555)	1.31 (0.61; 2.10)	1.44 (0.92; 2.23)
Adjusted for sex (n = 8,555)	1.00 (0.53; 1.86)	1.28 (0.82; 2.01)
Adjusted for sex & maternal education (n = 7,335)	1.09 (0.55; 2.13)	1.18 (0.72; 1.93)
**Age 17/18 TF4 clinic**		
Unadjusted model (n = 8,045)	1.07 (0.67; 1.71)	1.21 (0.86; 1.71)
Adjusted for sex (n = 8,045)	0.93 (0.58; 1.48)	1.07 (0.75; 1.52)
Adjusted for sex & maternal education (n = 6,786)	0.99 (0.59; 1.66)	1.04 (0.71; 1.54)
**Age 18/19 CCT questionnaire**		
Unadjusted model (n = 8,384)	0.99 (0.63; 1.57)	1.32 (0.95; 1.82)
Adjusted for sex (n = 8,384)	0.76 (0.48; 1.21)	1.04 (0.75; 1.44)
Adjusted for sex & maternal education (n = 7,066)	0.92 (0.56; 1.50)	1.12 (0.79; 1.59)
**Age 21/22 YPA questionnaire**		
Unadjusted model (n = 6,353)	1.29 (0.88; 1.91)	1.59 (1.17; 2.14)
Adjusted for sex (n = 6,353)	1.06 (0.72; 1.57)	1.33 (0.98; 1.81)
Adjusted for sex & maternal education (n = 5,300)	1.12 (0.74; 1.70)	1.38 (1.00; 1.91)
**Age 22/23 YPB questionnaire**		
Unadjusted model (n = 5,660)	1.22 (0.80; 1.86)	1.83 (1.36; 2.47)
Adjusted for sex (n = 5,660)	0.94 (0.61; 1.45)	1.50 (1.10; 2.03)
Adjusted for sex & maternal education (n = 4,668)	1.10 (0.69; 1.75)	1.65 (1.82; 2.30)

STROBE 2007 (v4) Statement—Checklist of items that should be included in reports of *cohort studies*

Section/Topic	Item #	Recommendation	Reported on page #
**Title and abstract**	1	(*a*) Indicate the study's design with a commonly used term in the title or the abstract	1 and 2
		(*b*) Provide in the abstract an informative and balanced summary of what was done and what was found	1 and 2
**Introduction**			
Background/rationale	2	Explain the scientific background and rationale for the investigation being reported	4 and 5
Objectives	3	State specific objectives, including any prespecified hypotheses	5
**Methods**			
Study design	4	Present key elements of study design early in the paper	5 to 8
Setting	5	Describe the setting, locations, and relevant dates, including periods of recruitment, exposure, follow-up, and data collection	5 to 7
Participants	6	(*a*) Give the eligibility criteria, and the sources and methods of selection of participants. Describe methods of follow-up	5 to 8
		(*b*) For matched studies, give matching criteria and number of exposed and unexposed	N/A
Variables	7	Clearly define all outcomes, exposures, predictors, potential confounders, and effect modifiers. Give diagnostic criteria, if applicable	5 to 9 (plus supplementary tables S1 to S3)
Data sources/ measurement	8*	For each variable of interest, give sources of data and details of methods of assessment (measurement). Describe comparability of assessment methods if there is more than one group	5 to 9 (plus supplementary tables S1 to S3)
Bias	9	Describe any efforts to address potential sources of bias	See discussion (pages 15 to 17)
Study size	10	Explain how the study size was arrived at	5 and 6 (and Table 1)
Quantitative variables	11	Explain how quantitative variables were handled in the analyses. If applicable, describe which groupings were chosen and why	5 to 9 (plus supplementary tables S1 to S3)
Statistical methods	12	(*a*) Describe all statistical methods, including those used to control for confounding	8 and 9
		(*b*) Describe any methods used to examine subgroups and interactions	N/A

For peer review only

		(*c*) Explain how missing data were addressed	See discussion (page 14 to 17)
		(*d*) If applicable, explain how loss to follow-up was addressed	See discussion (page 14 to 17)
		(*e*) Describe any sensitivity analyses	N/A
**Results**			
Participants	13*	(a) Report numbers of individuals at each stage of study—eg numbers potentially eligible, examined for eligibility, confirmed eligible, included in the study, completing follow-up, and analysed	5 to 9 (Tables 1, 3, S4 and S5)
		(b) Give reasons for non-participation at each stage	Table S4
		(c) Consider use of a flow diagram	N/A
Descriptive data	14*	(a) Give characteristics of study participants (eg demographic, clinical, social) and information on exposures and potential confounders	9 to 12 (Table 3)
		(b) Indicate number of participants with missing data for each variable of interest	9 (Tables 1 and S4)
		(c) Summarise follow-up time (eg, average and total amount)	N/A
Outcome data	15*	Report numbers of outcome events or summary measures over time	13 (Tables 1, 4, S7 to S11)
Main results	16	(*a*) Give unadjusted estimates and, if applicable, confounder-adjusted estimates and their precision (eg, 95% confidence interval). Make clear which confounders were adjusted for and why they were included	9 to 15 (Table 4, S7 to S13, S16, S17)
		(*b*) Report category boundaries when continuous variables were categorized	6 and 7
		(*c*) If relevant, consider translating estimates of relative risk into absolute risk for a meaningful time period	N/A
Other analyses	17	Report other analyses done—eg analyses of subgroups and interactions, and sensitivity analyses	17 (Table S17)
**Discussion**			
Key results	18	Summarise key results with reference to study objectives	15 to 18
**Limitations**			
Interpretation	20	Give a cautious overall interpretation of results considering objectives, limitations, multiplicity of analyses, results from similar studies, and other relevant evidence	3, 15 to 18
Generalisability	21	Discuss the generalisability (external validity) of the study results	16 to 18
**Other information**			
Funding	22	Give the source of funding and the role of the funders for the present study and, if applicable, for the original study on which the present article is based	20

For peer review only

*Give information separately for cases and controls in case-control studies and, if applicable, for exposed and unexposed groups in cohort and cross-sectional studies.

Note: An Explanation and Elaboration article discusses each checklist item and gives methodological background and published examples of transparent reporting. The STROBE checklist is best used in conjunction with this article (freely available on the Web sites of PLoS Medicine at http://www.plosmedicine.org/, Annals of Internal Medicine at http://www.annals.org/, and Epidemiology at http://www.epidem.com/). Information on the STROBE Initiative is available at www.strobe-statement.org.

For peer review only
